# EpiTools: An Open-Source Image Analysis Toolkit for Quantifying Epithelial Growth Dynamics

**DOI:** 10.1016/j.devcel.2015.12.012

**Published:** 2016-01-11

**Authors:** Davide Heller, Andreas Hoppe, Simon Restrepo, Lorenzo Gatti, Alexander L. Tournier, Nicolas Tapon, Konrad Basler, Yanlan Mao

**Affiliations:** 1 Institute of Molecular Life Sciences, University of Zürich, Winterthurerstrasse 190, 8057 Zürich, Switzerland; 2 Digital Imaging Research Centre, Faculty of Science, Engineering and Computing, Kingston University, Kingston-upon-Thames KT1 2EE, UK; 3 Institute of Applied Simulations, Zürich University of Applied Sciences, Einsiedlerstrasse 31a, 8820 Wädenswil, Switzerland; 4 SIB Swiss Institute of Bioinformatics, Quartier Sorge - Batiment Genopode, 1015 Lausanne, Switzerland; 5 Apoptosis and Proliferation Control Laboratory, Lincoln's Inn Fields Laboratory, Francis Crick Institute, 44 Lincoln's Inn Fields, London WC2A 3LY, UK; 6 MRC Laboratory for Molecular Cell Biology, University College London, Gower Street, London WC1E 6BT, UK

**Keywords:** image analysis, epithelial dynamics, network description

## Abstract

Epithelia grow and undergo extensive rearrangements to achieve their final size and shape. Imaging the dynamics of tissue growth and morphogenesis is now possible with advances in time-lapse microscopy, but a true understanding of their complexities is limited by automated image analysis tools to extract quantitative data. To overcome such limitations, we have designed a new open-source image analysis toolkit called EpiTools. It provides user-friendly graphical user interfaces for accurately segmenting and tracking the contours of cell membrane signals obtained from 4D confocal imaging. It is designed for a broad audience, especially biologists with no computer-science background. Quantitative data extraction is integrated into a larger bioimaging platform, Icy, to increase the visibility and usability of our tools. We demonstrate the usefulness of EpiTools by analyzing *Drosophila* wing imaginal disc growth, revealing previously overlooked properties of this dynamic tissue, such as the patterns of cellular rearrangements.

## Highlights

• Increasing time-lapse data increases the demand for automated image quantification
• EpiTools, an open-source image analysis toolkit, addresses this need
• EpiTools segments, tracks, and quantifies cell contours from 4D imaging data
• EpiTools application provides insight into cell dynamics in the *Drosophila* wing disc

## Graphical Abstract

**Figure:**
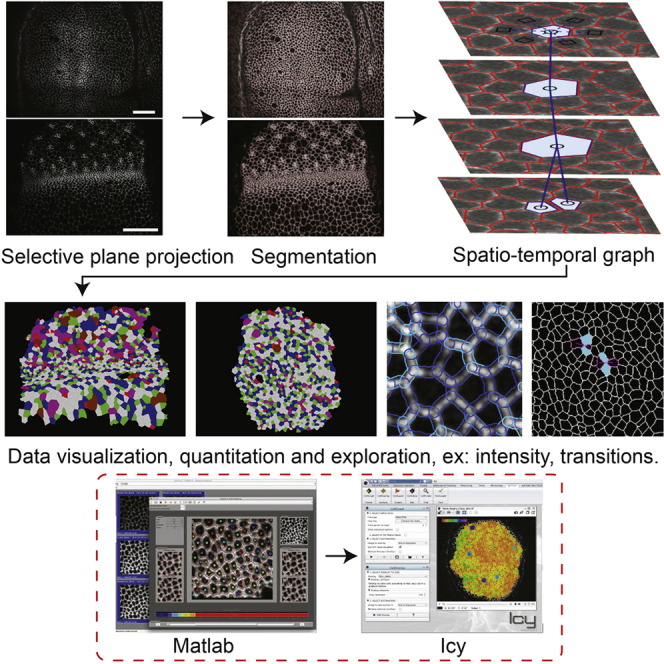

## Teaser

High-throughput analysis of time-lapse imaging data requires automated image quantification software. Heller et al. create a user-friendly image analysis toolkit, EpiTools, which segments, tracks, and quantifies cell contour data obtained from 4D imaging. They use this toolkit to obtain insight into cell dynamics in the developing *Drosophila* wing disc.

## Introduction

Multicellular tissues grow and develop in a complex and dynamic way. Final tissue size and architecture is determined by the coordination of cell divisions, cell death, cell shape changes, and cell rearrangements (Lecuit and Le Goff, 2007). Understanding the dynamic nature of how these processes are integrated in time and space is crucial to understanding tissue growth and morphogenesis. With recent advances in fluorescent light microscopy (Galland et al., 2015, Krzic et al., 2012), it has become increasingly possible to capture, at high temporal and cellular resolution, the dynamic processes of tissue growth and morphogenesis. This results in very large time-lapse datasets that are impossible to quantitatively analyze manually. The development of methods for automated image analysis, including cell segmentation and cell tracking, as well as analytical tools to quantitatively describe dynamic cellular behavior, is therefore the key to harnessing the power of in vivo imaging.

We have created EpiTools, a new image analysis toolkit for epithelial tissues. EpiTools is currently optimized for two-dimensional (2D) accurate cell contour segmenting and tracking of membrane labeled cells in epithelia, acquired originally as 4D (x, y, z, time) datasets using confocal microscopy. Aimed at a broad user audience, particularly biologists with little computer-science training, EpiTools has been designed to be easy to install and use, providing a guided analysis environment, yet being modular and extendable. We have ensured that the interfaces between the image segmentation and feature extraction modules are based on a simple standard format such that existing solutions, if preferred, can be readily used. The project Web site (http://tiny.uzh.ch/dm) delivers extensive support material and gives direct access to the software repositories, incentivizing modifications and extensions.

We have primarily used the *Drosophila* wing imaginal disc as an example tissue to demonstrate the functions of EpiTools. The *Drosophila* wing disc epithelium has been widely used as a model system to study the molecular and mechanical mechanisms of epithelial tissue growth (Aegerter-Wilmsen et al., 2012, Legoff et al., 2013, Mao et al., 2011, Mao et al., 2013, Shraiman, 2005). Until recent developments in ex vivo culturing of wing discs (Aldaz et al., 2010, Handke et al., 2014, Zartman et al., 2013), these studies had been limited to fixed tissue samples, masking the dynamic nature of the developmental process. Using EpiTools, we have now been able to fully exploit the power of the ex vivo culture and live imaging, to reveal new properties of this dynamically growing tissue which were previously overlooked. We have revealed new insights into how cell areas and cell shape (polygon) distributions change in different populations of cells as the epithelium develops, and how cell division orientations are regulated by cell shape. We have also systematically analyzed the spatial and temporal patterns of cell neighborhood relationships in the wing disc, and revealed patterns of cell intercalations and fluid-like junctional dynamics in a tissue previously thought to lack cell rearrangements (Bryant, 1970, Garcia-Bellido et al., 1973, Gibson et al., 2006, Resino et al., 2002).

## Design

Although several cell segmentation and tracking software suites have been developed (Table S1), including Packing Analyser (Aigouy et al., 2010), MorphographX (Barbier de Reuille et al., 2015, Kierzkowski et al., 2012), EDGE (Gelbart et al., 2012), Edge4D (Khan et al., 2014), (Blanchard et al., 2009), SeedWaterSegmenter (Mashburn et al., 2012), ilastik (Sommer et al., 2011), and TTT (Cilla et al., 2015), their adoption by the extended research community has often been slow. In particular, accessibility to biologists with limited computational experience has been a limiting factor. Moreover, due to the morphological diversity of biological systems, and therefore of acquired images, establishing a complete analysis pipeline for 3D time lapses presents many challenges. Several software packages often need to be combined and further extended by custom-written routines, which have to be adapted for each new biological question. The lack of user-friendly interfaces requires programming skills in various languages and handling of non-standardized file formats. Finally, connectivity to larger bioimaging platforms such as ImageJ or Icy, with which the user may already be familiar, is generally missing. Designed to overcome these limitations, EpiTools consists of a user-friendly image analysis framework with a graphical user interface (GUI) in MATLAB for processing of the raw images as well as a collection of software extension modules (plugins) for feature extraction, analyses, and visualization in Icy (de Chaumont et al., 2012). This modularity allows for processes to be replaced or extended with third-party techniques and tools.

## Results

### EpiTools Part 1a: An Image Segmentation Method for Epithelial Time-Lapse Data

**Figure 1:**
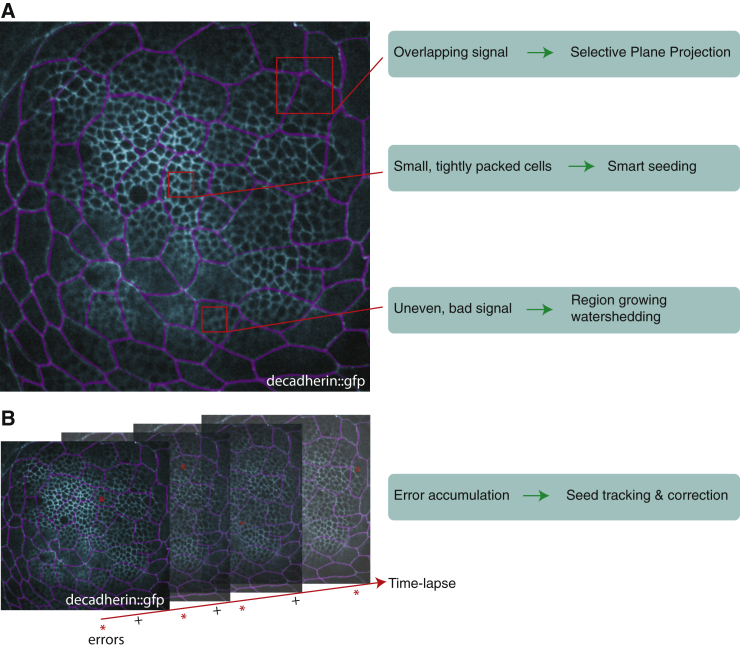
Image Segmentation Challenges (A) E-cadherin::GFP-expressing wing disc. A maximum-intensity projection of a 3D wing disc image taken from a 4D time lapse. The peripodial membrane cells (magenta) are directly above the disc proper cells (cyan). The two signals must be untangled for proper segmentation of disc proper cell shapes. This requires a selective projection approach. A second challenge is posed by the tightly packed nature of the wing disc proper epithelium. The small size of the cells makes it difficult to resolve individual membranes. For this reason, the seeding of the watershedding algorithm must be optimal. Finally, the E-cadherin::GFP signal varies largely through the wing disc, which complicates the watershedding approach. Here, a region-growing watershedding algorithm better suited to this task was developed. (B) The multiplication of time points in data series results in accumulation of errors (symbolized by asterisks). For this reason, the original segmentation must be as accurate as possible, but error correction steps must also be implemented.

**Figure 2:**
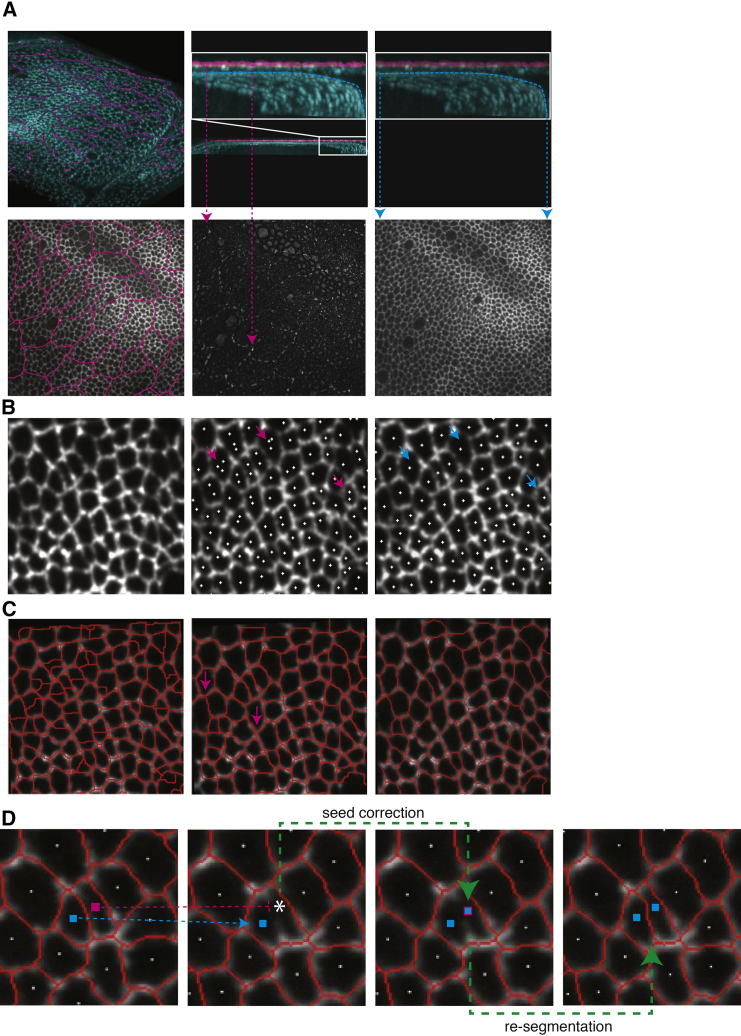
Image Segmentation Method (A) The *Drosophila* wing disc is a dome-shaped epithelial tissue consisting of two cellular layers: a dense mesh of disc proper cells (cyan) and a less dense mesh of peripodial membrane cells (magenta). Top center and right: a section through the image volume with the highlighted disc proper layer (cyan) and peripodial layer (magenta). The peripodial membrane needs to be separated from the wing disc, as these two layers can interfere with each other (bottom left: signal from both layers when maximally projected; bottom center: signal from just the peripodial layer). The selective plane projection accurately fits a surface to the disc proper layer (cyan line), taking into account its shape while excluding the peripodial layer. The result is a clean projected image (bottom right). (B) Automatic seed generation. Cut-out region from the selective plane projected image is shown after Gaussian smoothing has been applied (left). Wing disc projection with seeds generated from MATLAB watershed regions (center). Magenta arrows highlight some of the areas with multiple seeds per cell. Wing disc with seeds generated using our seed point generation algorithm (right). The number of duplicated seeds is greatly reduced, as highlighted by the cyan arrows. (C) Segmentation result of cellular regions overlaid on wing disc image. Cell boundaries generated from MATLAB watershed algorithm (left). Cell boundaries generated from MATLAB watershed algorithm with manually homogenized cell regions (middle). Magenta arrows highlight inaccuracies with the cell boundary segmentation. Our region-growing algorithm, using the same seed points as in the center image, generates more accurate cell boundaries (right). (D) Seed tracking allows identification of broken tracks due to segmentation errors, which are easily rectified and subsequently re-segmented. This process is depicted in the first three images while the last image shows the corrected segmentation. Cyan highlights correct seeds. The first two images highlight in magenta a broken track between two frames. The third image shows how the missing seed point can be added manually in the second image with a single mouse click. Re-segmentation then provides the correct boundary segmentation result.

Since many epithelial tissues consist of a cell monolayer, with cells growing, dividing, and moving in the plane of the tissue, a 2D planar projection of cell shapes is often a good approximation for understanding the dynamic behavior of the tissue. However, most epithelia are not flat sheets of cells, but can be considerably curved (Escudero et al., 2007, Osterfield et al., 2013, Sweeton et al., 1991), and may closely appose other cells or features outside of the plane of interest, which are inevitably captured during the imaging process. An example of such a challenging tissue is the *Drosophila* wing disc, which consists of two cellular layers on a dome-shaped surface: a dense mesh of columnar wing disc proper cells and a looser mesh of squamous peripodial cells on a different focal plane (Figures 1 and 2A). Large fluctuations in signal intensities, poor signal-to-noise ratios (to minimize tissue damage from long-term time-lapse imaging), and cells of varying sizes within the image volume make this a challenging segmentation problem. It is, however, critical to identify individual cells within the mesh so that the spatial and temporal relationships between neighboring cells can be quantified from the precise geometry of the membranes.

The following methods outline the image-processing procedures that EpiTools uses to achieve this task. We designed a selective plane projection that follows the curvature of the tissue in order to overcome the limitations of a simple maximum-intensity projection (Figure 2A). It consists of a two-step surface-fitting procedure and requires that voxels with high intensity mostly belong to the desired layer (e.g. the disc proper signal in the wing disc). We use the notion of stiffness to describe the flexibility of the fitting and interpolating method (D'Errico, 2006) to accommodate outliers lying far apart from estimated surface location (e.g. signal from peripodial membrane in the wing disc). In the first step of the method we choose an increased stiffness such that the fitted surface settles coarsely on the desired layer, ignoring points that lie far apart. This surface is used to exclude from the high-intensity signal points which have, with respect to the surface, a higher than threshold distance (e.g. peripodial membrane signal). In the second step a less stiff fitting is performed on the refined signal to follow the curvature of the desired layer accurately (Figure 2A, cyan line). Finally, pixel intensities along the fitted surface are used to form the projected image. The second 3D surface fit can be exported and used to correct subsequent geometric analyses, if necessary (Figure S1).

The projected images are aligned (registered) to compensate possible sample movement during the acquisition. The image registration step can also be performed through external software such as the StackReg plugin for ImageJ (Thevenaz et al., 1998) controlled via EpiTools using the MIJ interface (Sage, 2012).

On the registered images we apply region-growing segmentation. The aim of this step is to ideally first create a single seed point per cell from which to grow cellular regions (Figure 2B). Seed points conceptually represent cell centers and can be corrected with a simple mouse click to add, remove, or fuse cellular regions. Our seed point generation method was devised to include growing and merging of regions to reduce fragmentation: homogeneous regions of a certain size below a rising signal intensity threshold (cell boundary signal) are identified and allocated to become new cellular regions with a unique identification. Cellular regions are grown from these seed points by assimilating neighboring pixels below an increasing intensity threshold. The region growing is performed in parallel for each seed point and is guided by the local intensity flow, climbing up the intensity gradients that separate cells which create distinct boundaries between cells (Figure 2C). Finally, automatic temporal seed tracking has been implemented to identify seeding errors expressed as a discontinuity in temporal cell tracks, which leads to an efficient error correction (Figure 2D). For example, a missing seed point is identified by a broken track (Figure 2D, magenta track) often due to segmentation errors. The error can be easily rectified by adding a new seed (Figure 2D, seed correction) and re-segmentation can be applied, producing the final segmented frame (Figure 2D, re-segmentation). The final series of segmented frames can be exported as skeletons which accurately represent cell junction (or membrane) signals, as opposed to using linear approximations (Cilla et al., 2015). This is important because an accurate representation of the curvature of cell junctions is critical for understanding the mechanical properties of the cells (Brodland et al., 2014).

A detailed description of these processing steps can be found in Supplemental Experimental Procedures. All image-processing and analysis techniques were implemented in MATLAB (Mathworks). The region-growing and seed-tracking technique was implemented as compiled C extensions for MATLAB to reduce processing time.

### EpiTools Part 1b: Framework and User Interface, EpiTools-MATLAB GUI

**Figure 3:**
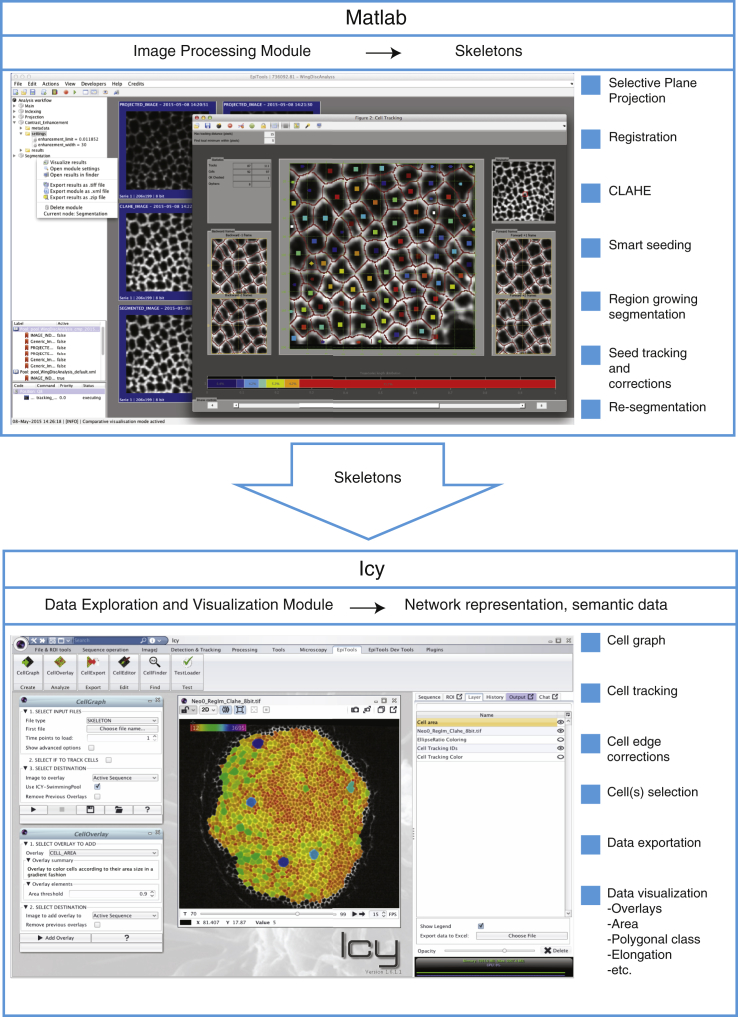
EpiTools Overview EpiTools consists of two complementary parts. First, an image-processing pipeline (Part 1, MATLAB) allows the user to preprocess the image prior to segmentation and to segment the image. The output of Part 1 consists of skeletons of the cell membranes. Part 2 uses Icy to provide a user-friendly interface for data visualization and analysis. An image-understanding module (CellGraph) enables the user to produce a cell graph, which gives a semantic description of the tissue of interest. The data exploration and visualization can be directly visualized as overlays in Icy (CellOverlay) or be exported as Excel sheets (xls) or graphML (xml) files (CellExport).

To optimize the ease of use, we split EpiTools into two parts (Figure 3). Part 1 is primarily a MATLAB (and C)-based analysis framework, with a bespoke GUI; Part 2 consists of EpiTools modules (plugins) for an existing image analysis platform, Icy (see below). Part 1 processes the images through the modular steps described above to eventually produce skeletons of the images that can be exported for further analysis in Part 2 (Figures 3 and S1). With the introduction of an EpiTools-MATLAB GUI, we wanted to expand the panorama of possibilities our end users have to complete their image analysis. The idea behind the current implementation was to separate the analysis workflow into single independent steps that can be called repeatedly for best parameterization as well as skipped if not needed. Moreover, we wanted this to be as easy and intuitive as possible, especially for users with little computer-science background.

The EpiTools-MATLAB GUI presents a series of menus where the user finds all the main components needed to run the analysis (see our video tutorials, which provide detailed step-by-step guides: http://tiny.uzh.ch/dN). Menus are divided according to function scope, and additional submenus guide the user to customize the analysis environment. We have also implemented context menus (right-click), which allow exporting and visualizing the connected files. The user can easily invoke them from the analysis workflow tree on the left side of the main window, clicking on the corresponding analysis module.

Each analysis module has a sub-window that collects all the procedures, inputs, and parameters required to execute it. We provide detailed explanations of every parameter (http://tiny.uzh.ch/dS) and recommended values to initially try. We designed a special independent GUI for the Seed Tracking module, highlighting the seeds that need corrections and offering various operations to assist the user in the manual corrections.

Parameter choices affect the output of many modules and can have lasting consequences on subsequent analysis steps. Therefore, we included a set of built-in visualization tools and comparison modes to help the parameterization. The comparative mode allows for easy comparison of different parameters to find the optimal parameter set for a given task. In addition, EpiTools offers connectivity to Icy, such that the user can make a more detailed analysis regarding the difference between the module executions (see Icy's Sequence comparator: http://icy.bioimageanalysis.org/plugin/Sequence_comparator).

The EpiTools Part 1 analysis files are created to achieve reproducible image analysis. User inputs, outputs, parameter sets, and all the associated metadata for each analysis step are stored in a clear xml file, which can be easily accessed from third-party applications.

### EpiTools Part 2: Network Analysis and Data Structure, EpiTools Icy

**Figure 4:**
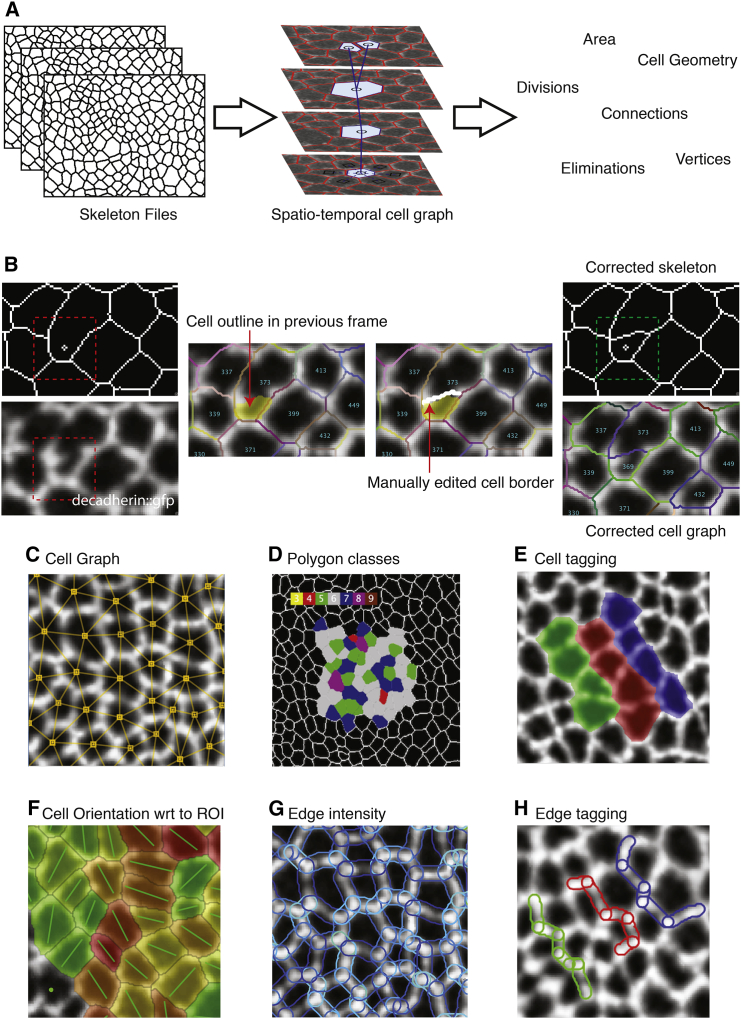
EpiTools Icy (A) The visualization and analysis module of EpiTools produces a cell graph based on the skeletons. The data structure provides a semantic understanding of the tissue and easy access to important values and events such as cell area, cell geometry, and cell divisions. (B) The cell editor plugin allows the user to employ abnormal changes in cell topologies to zoom into putative segmentation mistakes. If mistakes are identified, the user can use data from the unsegmented images to manually correct improperly segmented or missing cell borders. (C–H) The visualization and analysis module of EpiTools contains plugins that allow the user to generate and visualize data of interest. We use the overlay feature of Icy to superimpose the desired information onto skeletons or the original imaging time-lapse data. ROI, region of interest; wrt, with respect to. For a full list of overlays and explanations for each, please visit http://tiny.uzh.ch/dT.

The skeleton images produced by EpiTools Part 1 represent a common intermediary step (Aigouy et al., 2010, Mashburn et al., 2012), as yet unsuitable for manual analysis. It is therefore necessary to create a computational description of how the individual frames relate to each other and automatically capture changes. To this end, we developed EpiTools Part 2, a package that transforms the skeleton files into a computational graphic data structure (Figures 3 and 4A). This network-like data structure contains the neighborhood relationships between cells in the tissue in the form of nodes and edges. We use the term spatiotemporal graph to refer to this particular type of graph because we include both spatial (within the same frame) and temporal (between different frames) neighborhoods. Similar approaches can be found in the literature (Gelbart et al., 2012, Gunduz et al., 2004, Liu et al., 2010), and have been taken as inspiration for this approach.

We chose the bioimaging platform Icy (de Chaumont et al., 2012) as the framework for our package to provide rich visual feedback to the user. This software delivers remarkable user-friendliness and offers many image analysis tools for biological samples. In addition, the Icy platform facilitates sharing, in the form of plugins (i.e. software modules that extend the original capabilities) and analysis protocols. The main project Web site serves as a central hub to inform about available plugins and to allow user exchange. For more information about Icy, we recommend visiting the project homepage (http://icy.bioimageanalysis.org).

The EpiTools package for Icy consists of multiple plugins that address the subsequent steps of the analysis: CellGraph (Figure 4A), which generates the spatiotemporal graph starting from input skeleton files; CellEditor (Figure 4B), which enables the user to interactively modify the skeleton images manually in case of any remaining segmentation mistakes; CellOverlay (Figures 4C–4H, and S2), which interprets the data and outputs results in the form of graphical overlays (i.e. additional image layers) and tabular files; and CellExporter, which allows the user to export the complete numerical data in various formats, such as Excel and GraphML. Every plugin has a separate GUI and can be conveniently accessed through the EpiTools toolbar (see video tutorials at http://tiny.uzh.ch/dO). To facilitate data query, we have developed many commonly required analysis features (in the form of overlays, Figures 4C–4H), including, for example, cell areas, cell elongation ratios, cell intercalations, edge intensities, and more interactive features, such as how cell orientation changes with respect to a defined point of interest that can be interactively changed by the user (Figure 4F). For a full list of the overlays available (Figure S2), please visit http://tiny.uzh.ch/dT.

The spatiotemporal graph structure created by the CellGraph plugin is built in three main steps. First, the cell geometries are extracted from the supplied skeletons. Second, the geometry objects are inserted into a graph representing the spatial neighborhood based on intersection. Finally, temporal linkage is added by matching the spatial graphs representing each frame. To achieve this, we employ graph-matching algorithms and apply heuristics to analyze the unmatched cells. The latter might correspond to divisions or eliminations, or suggest a segmentation mistake.

We emphasized the visual elaboration of our graph structure because we found that visual analysis is very helpful in formulating hypotheses before exporting the data for statistical analysis. The overlays created by the CellOverlay plugin use the layer feature of Icy's image viewer and adapt automatically to the position in space and time. For example, Figure 3 (bottom half) shows an overlay that highlights all cell geometries with a gradient color scheme according to the apical cell area. The user is thus given a natural interpretation of how the area sizes are distributed in the tissue.

To quantitatively analyze the data, the user can generate an Excel sheet from every overlay focusing on the visualized quantities, or access more general export options through the CellExport plugin. Among many, we highlight the XML-based graph format called GraphML (Brandes et al., 2002), which stores the neighborhood relationships of the cells. The format can be easily read by many scripting languages such as R or Python. An example analysis file can be downloaded from the project homepage (http://tiny.uzh.ch/dP).

The surface estimated by the selective plane projection (EpiTools Part 1a) can be rendered as 3D Mesh ROI (Figure S1B) with the CellSurface plugin (http://icy.bioimageanalysis.org/plugin/3D_Mesh_ROI). Moreover, cells can be colored according to their estimated surface normal with the Projection overlay (Figure S1B′). For detailed information and utility, we refer the reader to http://tiny.uzh.ch/s3.

We implemented the Icy plugins in Java using two main libraries: the Java topology suite (http://www.sourceforge.net/projects/jts-topo-suite/) to manage the geometries of cells and the jgraphT library (http://www.jgrapht.org) to store the graph structure. Icy's shared plugin memory (swimming pool) is used to allow communication across the plugins. Please see Supplemental Information for more details.

For specific help on how to install and use our plugins, please visit our project Web site where we provide tutorials for every component (http://tiny.uzh.ch/dQ). The source code is provided with open-source license at the public Git repository https://bitbucket.org/davideheller/epitools/ and is provided here as a zip file (Data S1).

### Analysis of Different Epithelia using EpiTools

**Figure 5:**
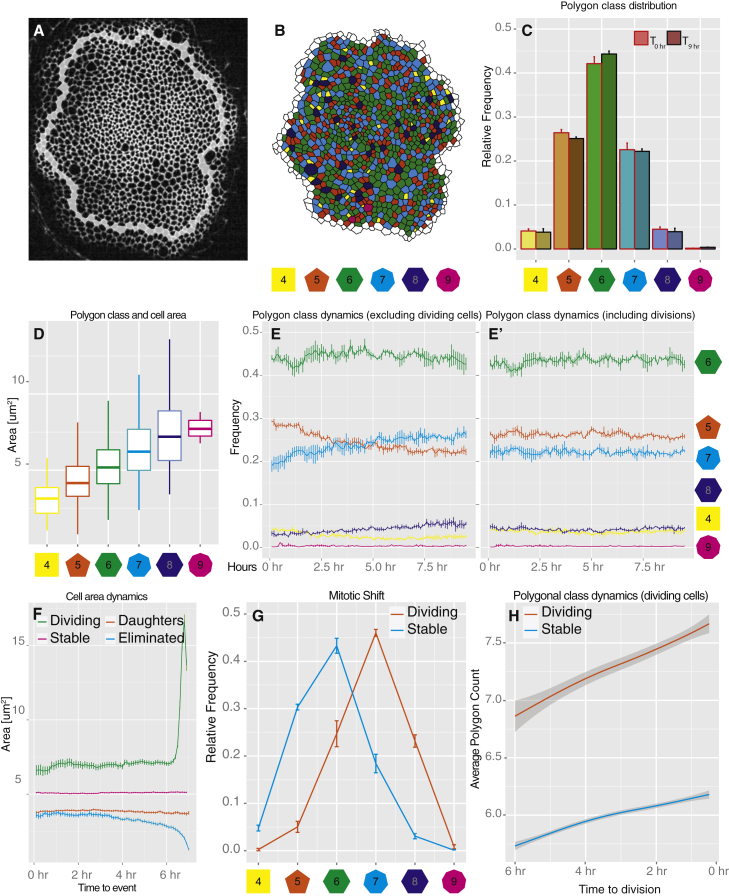
*Drosophila* Wing Disc: Polygon Distribution and Cell Area Dynamics If not stated otherwise, error bars indicate SEM in all figures. (A) Representative example of the wing pouch section of a wing disc (E-cadherin::GFP). Overlaid white cells represent the segmentation border. (B) Same frame as (A), segmented and processed with EpiTools. The polygon class of each cell is indicated by a color code. (C) The frequency of the polygon classes corresponding to cells with n number of neighbors remains constant over time. (D) On average, the area of cells correlates well with their polygon class. However, there is a large degree of variance. Boxplot whiskers indicate 1.5× interquartile range (IQR), hinges IQR, and inner lines the median. (E and E′) Among non-dividing cells the frequency of certain polygon classes increases over time (E). This effect is no longer visible when dividing cells (E′), and thus daughter cells are included in the analysis. (F) Area comparison between cell classes over time. First, cells that can be observed for at least 1 hr (10 frames) are selected from three samples (3,086 cells) and classified according to four classes: daughters (observed offspring in the movie, n = 686), dividing (observed dividing in the movie, n = 303), eliminated (n = 88), and stable (remainder, n = 2,009). Second, to compare area sizes across classes we transform the temporal axis with respect to the class: daughter's origin time (frame after division) is aligned to 0 hr, dividing cell's ending frame (frame of division) is aligned to 7 hr and equally so for eliminated cells (frame before elimination). For stable cells we used 7 hr of continuous observation beginning from the start of the movie. (G) EpiTools correctly detects the expected +1 shift in the frequency of polygon classes among dividing cells versus non-dividing cells. (H) Both dividing cells and non-dividing cells accumulate neighbors over time. However, the increase is twice as fast for dividing cells. Envelope indicates 0.95 confidence level.

To test the versatility of EpiTools, we processed different epithelia in *Drosophila* with varying cell areas and cellular heterogeneities (Figure S3). The *Drosophila* wing imaginal disc (Figures 5A and S3A) was our main tissue of focus (see sections below), but EpiTools was also able to segment, with high precision, membrane signals from time-lapse images of *Drosophila* eye imaginal discs (Figure S3B), histoblast nests (Figure S3C), and embryos (Figure S3D). We show here mainly the results of single frames for ease of representation. Although there are still some segmentation errors visible in Figures S3B–S3D, these were deliberately obtained without any manual corrections, showing the high accuracy of the automated segmentation process, provided that correct parameters are used (see Experimental Procedures, Table 1, and our guides to parameters on our Web site http://tiny.uzh.ch/dS). In the eye imaginal disc, we were able to track the rearrangements of cells as they exit the morphogenetic furrow (Figure S4, 0 min) through their formation into arcs (60 min), to their eventual recruitment into ommatidial preclusters (240 min).

### *Drosophila* Wing Disc Analysis I: Proof of Principle and Insights into Epithelial Geometry and Cell Division Dynamics

Epithelia assume cell-packing geometries characterized by different cell areas and neighbor-number distributions. Cells can be classified by their number of neighbors into sets of polygons. Interestingly this geometric order tends to remain apparently unperturbed by the changes introduced by cell divisions (Farhadifar et al., 2007, Gibson et al., 2006). We used EpiTools to study the geometric order of the *Drosophila* wing disc, dynamically, on growing discs. Of note, previous quantifications were mainly done on fixed samples, whereas in this study we examine live discs. In this way we can directly assay the interplay between epithelial dynamics and cell division. The quantitative data generated with EpiTools agrees well with previous reports and expectations, but also provides insights into the interplay between cell divisions and epithelial geometry.

First, we examined the frequency of n-sided cells in wing discs. We obtained polygon frequencies in good agreement with previous reports (Gibson et al., 2006; Figure 5C). A comparison of the polygon distribution 6 hr apart confirms that the frequencies remain constant (Figure 5C). Previous reports have indicated that in the wing disc, cell area correlates with polygon count, thus obeying Lewis's law (Farhadifar et al., 2007, Lewis, 1928). Our data confirm this, but show a large degree of variation (Figure 5D). Next, we looked at whether differences in cell geometry also correlated with different cell fates (such as dividing cells and dying cells). Since we are now able to track cells and have a semantic interpretation of the time lapse, we can select specific cell classes based on their behavior: dividing cells, new (daughter) cells, stable cells, and eliminated cells. The apical area of daughter cells is half that of dividing cells (Figure 5F). This indicates that, assuming cell height remains constant, wing disc cells double in volume prior to division. Our data also indicate that stable cells are larger than daughter cells (Figure 5F) and are likely a population of cells that is either in S or G1. Furthermore, we identified cells that are eliminated during the recordings (Figures 5F and S5). Interestingly, these cells can be identified as the smallest class of cells (Figure 5F). Further analyses of these cells revealed that they were eliminated by a process reminiscent of live cell delaminations. To better study this phenomenon, we employed the edge-tracking feature of EpiTools. By tracking the edges of the delaminated cells, we quantified the intensity of the E-cadherin signal over time and confirmed that E-cadherin signal intensity did not diminish prior to elimination (Figures S5A″ and S5D). The stability of E-cadherin is a hallmark of live cell delamination, where E-cadherin is not reduced on cell junctions, and can be used to differentiate this type of cell elimination from apoptosis, whereby E-cadherin is lost from junctions prior to cell elimination (Marinari et al., 2012). We then confirmed that delaminating cells seemed to, on average, lose edges prior to delamination (Figure S5E), a second characteristic of live cell delaminations (Marinari et al., 2012).

Next, we looked at how the polygon count of cell classes evolves over time. Interestingly, among the cells that were not observed to divide during our imaging window (Figure 5E) the frequency of n-sided neighbors does not remain constant. Specifically, the frequency of pentagons decreases while that of heptagons increases (Figure 5E). However, if one considers the population as a whole, this effect disappears (Figure 5E′). This supports the idea that the allocation of neighbors after cell division contributes to keeping the fraction of n-sided cells constant (Gibson et al., 2006).

We found that mitotic cells have on average one extra side compared with stable cells (Figure 5G), as expected (Gibson et al., 2006, Gibson et al., 2014). This can be observed several hours prior to division, confirming that dividing cells have been accumulating neighbors over time (Figure 5H) (Gibson et al., 2014). All cells tend to have an increase in number of neighbors over time, but this effect is stronger for dividing cells than for stable cells (Figure 5H).

**Figure 6:**
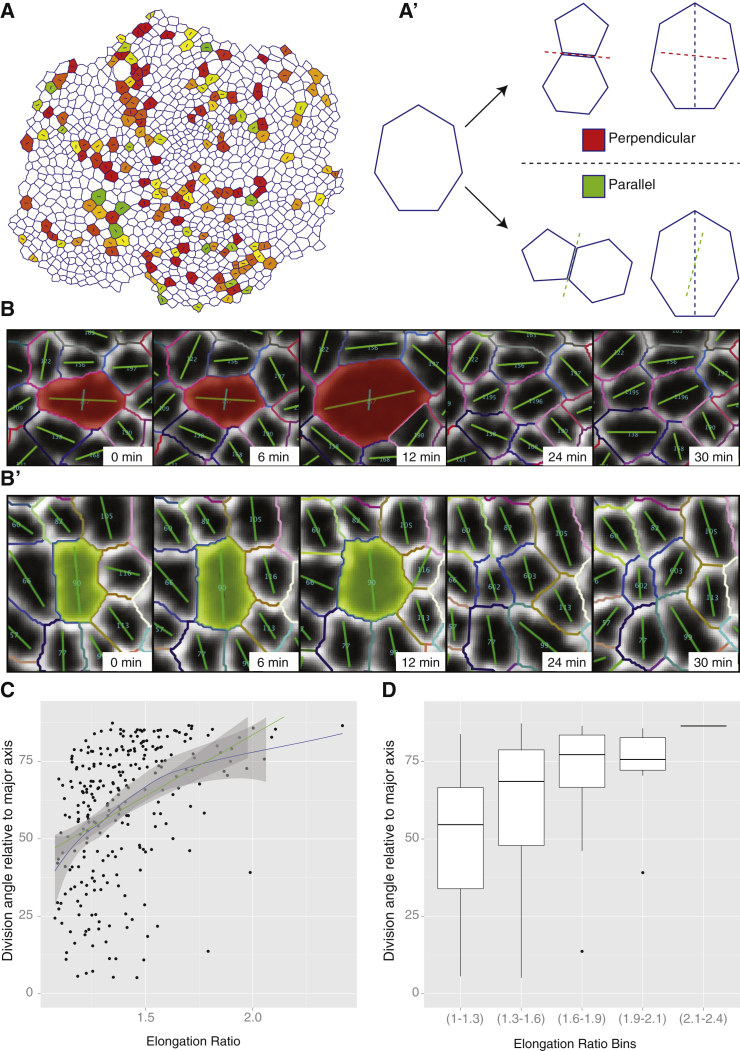
Control of Cell Division Orientation in the *Drosophila* Wing Disc If not stated otherwise, error bars indicate SEM in all figures. (A and A′) Overview of the orientation angle of the division axis relative to the long axis of the cells. Note that contrary to expectations, some cells divide parallel to the long axis (red equals fully perpendicular, green fully parallel). (B and B′) Representative montages of a perpendicular division and a parallel division, respectively. (C and D) The orientation of the division angle relative to the long axis of the cell depends partly on the aspect ratio of the dividing cells. Cells with a small aspect ratio show more variance, but there is a trend toward more perpendicular divisions as the aspect ratio increases. (C) Every black dot corresponds to one division statistic; green line is a simple linear model fit; blue line is an adaptive local polynomial regression; envelope indicates 0.95 confidence level. (D) Boxplot whiskers indicate 1.5× IQR, hinges IQR, and inner lines the median.

Another well-studied phenomenon that we analyzed with EpiTools is the link between cell geometry and cell division orientation. Consistent with previous studies in the wing disc (Gibson et al., 2011, Mao et al., 2011) we found that cells that are significantly elongated, with an elongation ratio (major/minor axis) greater than 1.3, tend to divide to bisect their long axis, i.e. the new junction is near perpendicular to the long axis of the cell (Figures 6A′, 6C, and 6D). However, some cells do not obey this rule and divide to form the new junction parallel to the long axis of the dividing cell (Figures 6B′, 6C, and 6D). Although most of these cases are for cells that are not significantly elongated (elongation ratio less than 1.3), whereby ellipse fittings could be introducing errors in the estimation of the long axis, occasionally even significantly elongated cells can divide to bisect their short axis (Figure 6B′). Without an automated segmentation and unbiased high-throughput analysis method, it would have been difficult to identify such outliers, which may uncover additional, previously overlooked factors that regulate cell division orientation.

### *Drosophila* Wing Disc Analysis II: Epithelial Junction Dynamics

**Figure 7:**
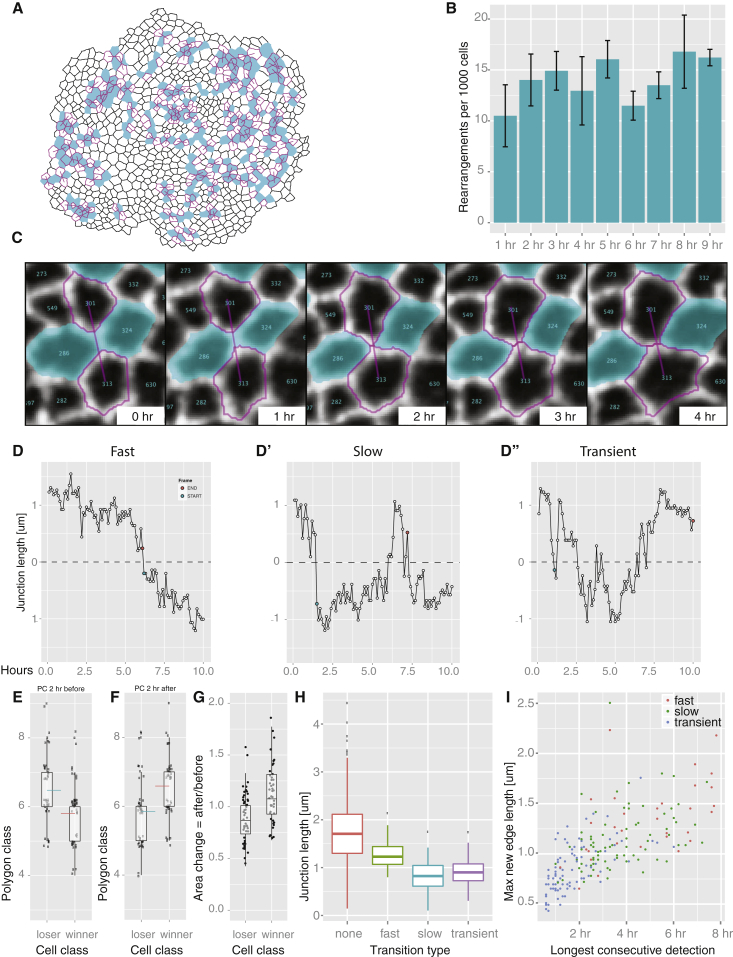
T1 Transitions in the *Drosophila* Wing Disc If not stated otherwise, error bars indicate SEM in all figures. All boxplot whiskers indicate 1.5× IQR, hinges IQR, and inner lines the median. (A) All T1 transitions detected over a 10-hr period traced back to the first time point. Linked magenta cells will intercalate in between cyan cells. (B) Transition frequency remains constant during the duration of the imaging session. (C) Representative montage of a T1 intercalation. (D) T1 transitions follow different dynamics. Here we classified them into fast (D), slow (D′), and transient (D″). See Figure S6 for classification rules. (E) Cells that will lose an edge during a transition are of a higher polygon class, on average 2 hr before the transition, than those that will gain an edge. Blue and red lines indicate the mean. (F) Cells that gain an edge during a transition are of a higher polygon class, on average 2 hr after the transition, relative to cells that lost an edge. Blue and red lines indicate the mean. (G) Transitions are associated with a change in cell area. On average, the area of cells that lose an edge decreases after the transition while it increases for the cells that gained an edge. (H) Cells that do not undergo transitions have longer junctions than transitioning cells. Furthermore, rapidly transitioning cells have longer junctions than slowly or transiently transitioning cells. (I) Rapidly transitioning junctions reach a greater length and are more stable than slowly or transiently transitioning junctions.

Apart from quantifying cell geometries, the network abstraction created by the CellGraph plugin of EpiTools also allowed us to analyze the evolution of cellular neighborhood relationships during tissue development and detect any neighbor-exchange events, such as intercalations (also known as T1 transitions; Bertet et al., 2004, Farhadifar et al., 2007). Historically, it has been assumed that very few T1 transitions occur in the proliferating wing imaginal disc, as cells from the same lineage (clones) remain as intact clusters and do not disperse, suggesting that cells adhere tightly to their neighbors (Bryant, 1970, Garcia-Bellido et al., 1973, Resino et al., 2002). Previous attempts at manually tracking a few cells in the proliferating wing disc have also failed to detect significant cell rearrangements (Gibson et al., 2006). With our automated and systematic high-throughput analysis methods, we were able to detect a significant number of T1 transitions (Figure 7C), averaging at 13 transitions per 1,000 cells per hour over a 10-hr imaging window (an average total of 129 transitions in 1,000 cells over 10 hr) (Figure 7B). There were no significant changes in the frequency of T1 transitions over the 10-hr imaging window, suggesting that these transitions are not an artifact of the ex vivo culture. Upon analysis of the spatial distribution of these transitions across the epithelia, we could not detect any clear patterns of transition clustering or directionality (Figure 7A). We did find that for the four cells involved in a T1 transition, the pair that would gain an edge (winners) frequently started the transition as hexagons or pentagons, and would finish the transition as heptagons or hexagons, whereas the pair that would lose an edge (losers) would generally start as heptagons or hexagons and finish as hexagons or pentagons (Figures 7E and 7F). In other words, the cells that have a larger number of sides would “lose” an edge to the cells that have a lower number of sides during a transition process. Consistent with Lewis' law (Lewis, 1928) the cells that gain an edge also increase their apical area after the transition, whereas cells that lose an edge are smaller after the transition (Figure 7G).

To gain a more quantitative understanding of the dynamics of these T1 transitions, we tracked the dynamic fluctuations of the length of each junction over 10 hr. As most junctions did not change their length significantly, we focused our analysis on the junctions that would shrink to a length of zero and then be substituted by a new growing junction (which we plot as negative values in Figure 7D). These are effectively T1 transitions. As a result of this analysis we noticed distinct “classes” of junctional dynamics. In an attempt to systematically classify these, we designed an algorithm to classify the transitions into three classes: fast, slow, and transient (Figures 7D, 7D″, and S6). In fast transitions (18% of total transitions), cells very efficiently exchange neighbors and the new neighborhood relationship remains stable. In slow transitions (37% of total transitions), the new neighborhood relationship eventually stabilizes, but takes longer to reach this stable state, while transient transitions (45% of total transitions) constantly fluctuate between the old and new neighborhood configurations. These definitions depend on the imaging window, but provide a method to quantify and classify the transition dynamics. In principle, if imaging windows were not limited, junctions would fluctuate between these dynamic states and show a continuum of behavior along this dynamic spectrum. On average, the longest junctions do not show any transitions (Figure 7H), but the junctions that undergo fast and decisive transitions are longer than the slow and transient transitions. Thus, it is not simply that longer junctions take longer to shrink to zero and grow again in the orthogonal direction, suggesting that the fast T1 transitions may have a separate mechanism of regulation. After the neighborhood exchange, the new junctions formed as a result of the fast and slow transitions grow to a longer length and remain stable for a much longer period of time than the transient transitions that fluctuate back and forth around the four-way vertex (Figure 7I). Whether these different classes of T1 transitions are fundamentally different in their regulation and function remains an interesting question for future research.

## Discussion

Advances in time-lapse imaging methods have resulted in very large datasets that are becoming impossible to analyze without robust quantitative tools. To address this pressing issue, we have created a new image analysis toolkit for epithelial tissues called EpiTools, which is aimed at biologists with little computer-science background, although the source code is also available should the user wish to extend or modify it for their own needs.

The main strength of EpiTools is its modularity. Splitting EpiTools into two parts gives our users more flexibility. The modular format of EpiTools Part 1 is designed for segmenting time-lapse images and outputting digitalized skeletons of cell outlines for further quantification, whereby users can use EpiTools Part 2 for, or their favorite existing software. Similarly, if users have already segmented their images with other software, they can use EpiTools Part 2 for further morphometric quantifications. The integration of EpiTools Part 2 into a larger bioimaging platform, Icy, that many users are already familiar with, makes it more accessible and user-friendly. Importantly, EpiTools allows for the easy manipulation of segmentation parameters, so that users can adapt the pipeline to the geometric idiosyncrasies of their biological system of choice. We believe these improved flexibility and user-friendly features will ensure that more users will adopt EpiTools for their image segmentation, tracking, and quantification, which is in increasing demand with the current rise of time-lapse microscopy.

There have been other image segmentation and analysis software available, each with its own strengths and weaknesses. We have tried to summarize the different features of each in Table S1. This will hopefully allow users to decide which one best suits them. Indeed there is no software that fits all criteria. Our decision to develop a new set of tools rather than to rely on previously published techniques was due to multiple reasons. Closed source code base (Packing Analyzer), and/or requirements for specific hardware (MorphoGraphX), excluded some solutions. Furthermore, the apical localization of the junctional marker E-cadherin and the limited tissue penetration also denied the use of volumetric-based methods such as EDGE or EDGE4D. SeedWaterSegmenter offered good segmentation performance but was problematic for projection and curation of long time series. A major drawback of all discovered solutions (Table S1) was also the lack of native interfaces to known imaging platforms such as FIJI (ImageJ) or Icy. We valued the latter because we think that exploratory analysis must be assisted by known, reliable, and easy interfaces. Powerful visualization features and easy image interaction result in much more intuitive data exploration for the scientist. In line with this argument we also concentrated our efforts on generalizing the EpiTools toolbox enough to allow widespread adoption. The image-processing part (MATLAB) does not require specific data dimensionality or format (e.g. 3D, 2D, time) through use of the bioformats library, ensuring that the user can start from multiple entry points. Parameter choice, which is usually not retained between iterations and leads to difficult decision processes, is aided by an easy GUI. Here we allow the user to review and choose among several runs of the same function. Finally, we simplified the setup procedure. Indeed we noticed that advanced installation procedures, while obvious to the creators, are a major deterrent for widespread adoption (e.g. in TTT, itk/vtk custom compilation, and EDGE3D). Thus, the setup procedure was simplified to allow a simple drag-and-drop procedure without compilation of additional libraries.

With the current version of EpiTools, we have primarily analyzed the epithelial growth dynamics of the *Drosophila* wing imaginal disc, and reproduced data in agreement with previous work, such as the polygonal packing patterns of the epithelia in different cell populations (Gibson et al., 2006, Gibson et al., 2014). We also noticed that although most cells divide to bisect the long axis of the dividing mother cell, as previously reported in the wing disc (Gibson et al., 2011, Mao et al., 2011) and other systems (Hertwig, 1893, Morin and Bellaïche, 2011, Ragkousi and Gibson, 2014), there was also a significant population of cells that did not obey this rule. Understanding the nature of such divisions, and attempting to distinguish whether it is cell geometry (shape) (Minc et al., 2011, Wyatt et al., 2015), the sensing of tension anisotropy of the cell (Campinho et al., 2013, Fink et al., 2011, Mao et al., 2013), or the effect of neighboring cell topologies (Gibson et al., 2011), will be easier to pursue with the dynamic quantitative tools now available in EpiTools and its combination with force-inference methods such as CellFIT (Brodland et al., 2014).

We also analyzed the dynamic patterns of cell rearrangements (T1 transitions) and junctional fluctuations in the wing disc, and revealed that the junctions are more mobile and the tissue more fluid-like than previously thought. As there appears to be no clear spatial patterns and orientations to these T1 transitions, it is unclear whether they have a functional significance or whether they are just passive consequences of tissue homeostasis. The fact that it is consistently the cells that have a larger number of sides that “lose” an edge to the cells that have a smaller number of sides does suggest that T1 transitions may have a role in maintaining the conserved polygonal packing geometry observed in many epithelia (Gibson et al., 2006) and, perhaps, buffer heterogeneities induced by cell divisions (Figures 5E and 5E′). Without regulated T1 transitions, cells would either always adhere tightly to their original neighbors or intercalate too freely, neither of which would allow the necessary mechanical tensions and cell geometries to emerge in the tissue to pattern cell divisions and tissue growth (Legoff et al., 2013, Mao et al., 2013).

Purely based on tissue dynamics, it is of course unclear how these T1 transitions are regulated. If this process were driven purely by a “passive” force equilibration process, one would expect the longest junctions to be the most stable, which is true to a certain extent (Figure 7). However, the fastest (and most stable/irreversible) T1 transitions actually occur in junctions that are normally longer than the slow/transient T1 transitions, suggesting that there may be a more active mechanism at play in regulating these T1 transitions. Extensive studies of T1 transitions in the *Drosophila* embryo have shown that a cell-autonomous accumulation of non-muscle Myosin II at the shrinking junctions during the first phase of the transition is required for the increase in cortical tension and shortening of that junction (Bertet et al., 2004, Rauzi et al., 2008, Zallen and Wieschaus, 2004). However, extrinsic forces can also induce cell rearrangements (Aigouy et al., 2010, Sugimura and Ishihara, 2013). Future studies of cortical tension and Myosin II dynamics may therefore be needed to assess the regulatory mechanism and possible function of T1 transitions in the wing imaginal disc.

### Limitations

We have designed EpiTools so that it supports most imaging file formats, but there are a few limitations. EpiTools Part 1 accepts 8- or 16-bit bioformat compatible images with two additional requirements: (1) information regarding one time point cannot be distributed across multiple files; (2) the used file extension has to be included in the user-settings file (for more information see Supplemental Experimental Procedures). The preferred format is single TIFF file for every time point. For EpiTools Part 2, skeleton files should be 8-bit binary images. Again, the preferred format is TIFF.

The major limitation of EpiTools is that in its present form our toolbox is not suited for volumetric 3D analysis. We accept that biological datasets are too heterogeneous to allow for a unique solution for data processing and analysis, hence different software is required (Table S1). As the data quality from volumetric 3D imaging improves, we aim to add full 3D volumetric analysis to our toolkit. The modular and open-source nature of EpiTools makes it an ideal platform to develop new features.

In summary, we have generated a series of accessible tools aimed at harnessing recent advances in optical microscopy to produce a quantitative description of epithelial tissue morphogenesis. We anticipate that these tools will greatly facilitate the study of tissue dynamics in development and disease.

## Experimental Procedures

### License Information

To encourage the sharing of resources, EpiTools is published under an open-source (GPLv3) license, which can be downloaded from http://tiny.uzh.ch/mM.

### Live Imaging

Wing discs were cultivated ex vivo and imaged as described by Zartman et al. (2013). However, the discs were not encapsulated in an alginate gel, as we have found that this step can be omitted without negatively affecting the imaging. A total of 3 E-cadherin:GFP-expressing wing discs (Huang et al., 2009) were imaged over 10 hr each, from around 100 hr after egg laying.

### Segmentation Parameters

The parameters used for segmenting the wing disc time lapses and other images shown in Figure S3 are shown in Table 1.

### Measurement of the Division Orientation

We define the division orientation as the angle between the longest axis of a mother cell before division and the new junction between the two daughter cells after division. To reliably measure the angle, we decided to average multiple temporal combinations such that individual frame differences would not affect our result. The longest elongation axis of a mother cell was retrieved using five time points from 72 min to 42 min before the division when the two daughter cells are first visible (the acquisition interval was 6 min). The reason to exclude the time points in the immediate vicinity of the division (i.e. 36 min to 6 min before) was to avoid including the apical rounding phase of mitosis whereby the increasingly circular cell shape makes the longest elongation estimation unreliable. The new junction was also measured in five time points after the division. Specifically for each frame the segment between the two centroids of the daughter cells was computed, and the angle of the new junction was computed as being perpendicular to this segment. The final average value for the division orientation is the mean of the 25 possible combinations.

## Author Contributions

D.H. designed and implemented EpiTools Part 2, analyzed the data, and wrote the article. A.H. designed and implemented the image-processing algorithms for EpiTools Part 1, and wrote the article. S.R. performed the biological experiments, designed EpiTools Part 2, analyzed the data, and wrote the article. L.G. implemented EpiTools Part 1 GUI, analyzed the data, and wrote the article. A.L.T. designed and implemented the image-processing algorithms for EpiTools Part 1. N.T. and K.B. designed the research. Y.M. designed the research, analyzed the data, and wrote the article.

## Figures and Tables

**Table 1:** Parameters Used for Segmenting Images

Web Site Name	XML Code	Wing	Eye	Histoblast	Embryo
Projection					
Smoothing Radius	SmoothingRadius	1	1	1	1
Surface Smoothness 1	SurfSmoothness1	30	30	30	30
Cutoff distance	ProjectionDepthThreshold	1.2	1.2	1.2	1.2
Surface Smoothness 2	SurfSmoothness2	20	20	20	20
CLAHE					
Enhancement limit	enhancement_limit	0.02	0.02	#	0.02
Enhancement width	enhancement_width	30	30	#	30
Segmentation					
Gaussian Blur Kernel	sigma1	2	1	1	1
Minimum cell area	mincellsize	25	15	25	25
Minimal membrane intensity	threshold	25	20	25	25
Boundary Low Intensity Ratio	MergeCriteria	0.15	0.45	0.35	0.35
Gaussian Blur Kernel	sigma3	0.5	2	2	2
Largest Cell Area	LargeCellSizeThres	3,000	4,300	3,000	3,000
Minimal mean intensity	IBoundMax	30	20	30	30

## Supplemental Information

Document S1. Supplemental Experimental Procedures, Figures S1–S6, and Table S1

Data S1. Source Code

Document S2. Article plus Supplemental Information
