# Supplementary material for: EpiTools: An Open-Source Image Analysis Toolkit for Quantifying Epithelial Growth Dynamics
Source: Dev Cell. 2016 Jan 11;36(1):103–16. doi: 10.1016/j.devcel.2015.12.012 (PMC4712040; doi:10.1016/j.devcel.2015.12.012)
Supplement: Document S1. Supplemental Experimental Procedures, Figures S1–S6, and Table S1 [file mmc1.pdf]

Developmental Cell, Volume *36*

Supplemental Information

EpiTools: An Open-Source Image Analysis Toolkit for Quantifying Epithelial Growth Dynamics

Davide Heller, Andreas Hoppe, Simon Restrepo, Lorenzo Gatti, Alexander L. Tournier, Nicolas Tapon, Konrad Basler, and Yanlan Mao

INVENTORY OF SUPPLEMENTAL INFORMATION

Supplemental Figures

Figure S1, related to Figure 3. EpiTools Complete workflow
Figure S2, related to Figure 4. EpiTools Icy CellOverlay examples
Figure S3, related to Figure 2. Different epithelia analyzed using EpiTools.
Figure S4, related to Figure 6. Ommatidial cell rearrangements in Drosophila eye imaginal disc
Figure S5, related to Figure 5. Cell delaminations in the Drosophila wing disc
Figure S6, related to Figure 7. Flowchart for the identification and classification of T1 transitions in the wing disc samples.

Supplemental Tables

Table S1, related to Figure 1. Comparison of existing image segmentation and tracking software

Supplemental Experimental Procedures

Source code
EpiTools Part 1a: Matlab and C++ modules
EpiTools Part 1b: EpiTools-Matlab-GUI
EpiTools plugins for Icy
Supported file formats
Quick Guides for EpiTools

Supplemental References

Figure S1

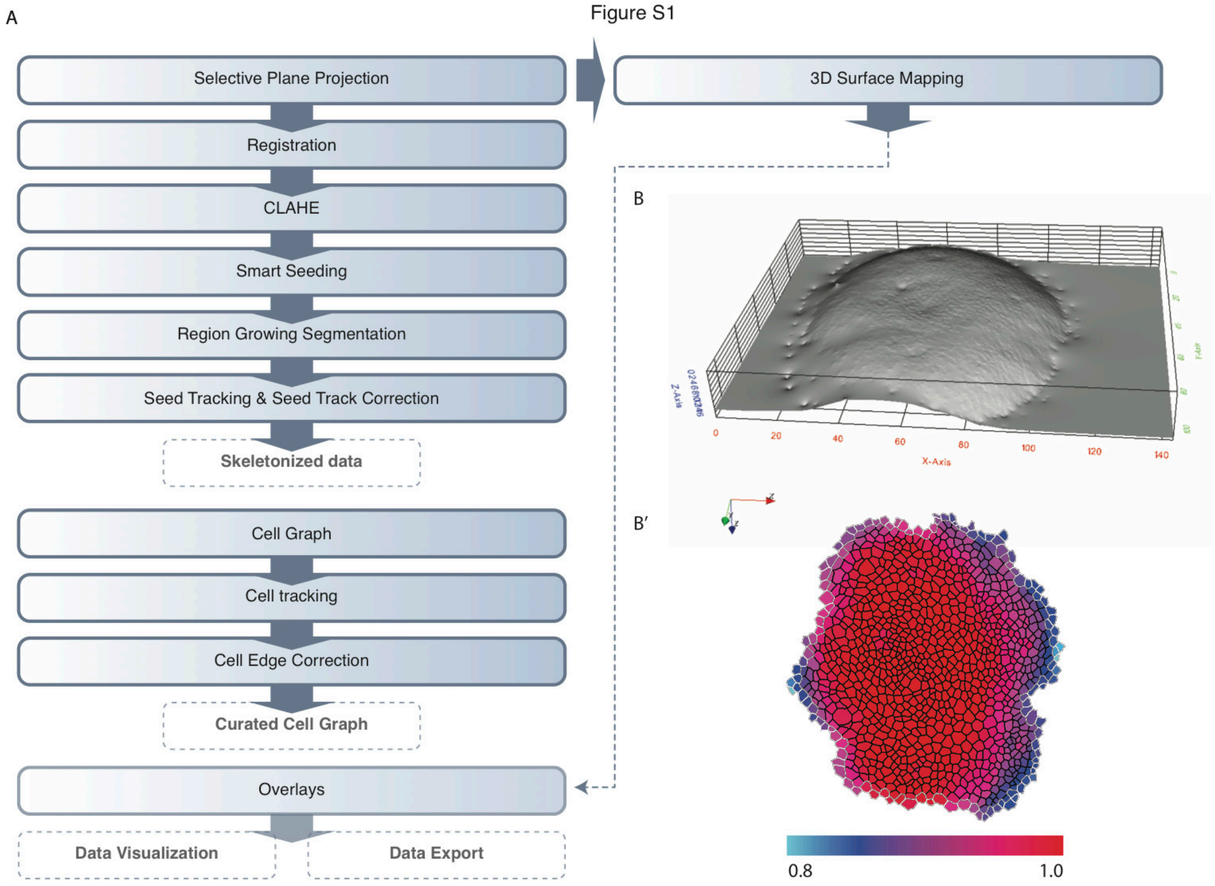
A
Selective Plane Projection
Registration
CLAHE
Smart Seeding
Region Growing Segmentation
Seed Tracking & Seed Track Correction
Skeletonized data
Cell Graph
Cell tracking
Cell Edge Correction
Curated Cell Graph
Overlays
Data Visualization
Data Export
3D Surface Mapping
B
X-Axis
Z-Axis
Y-Axis
B′
0.8
1.0

Figure S2

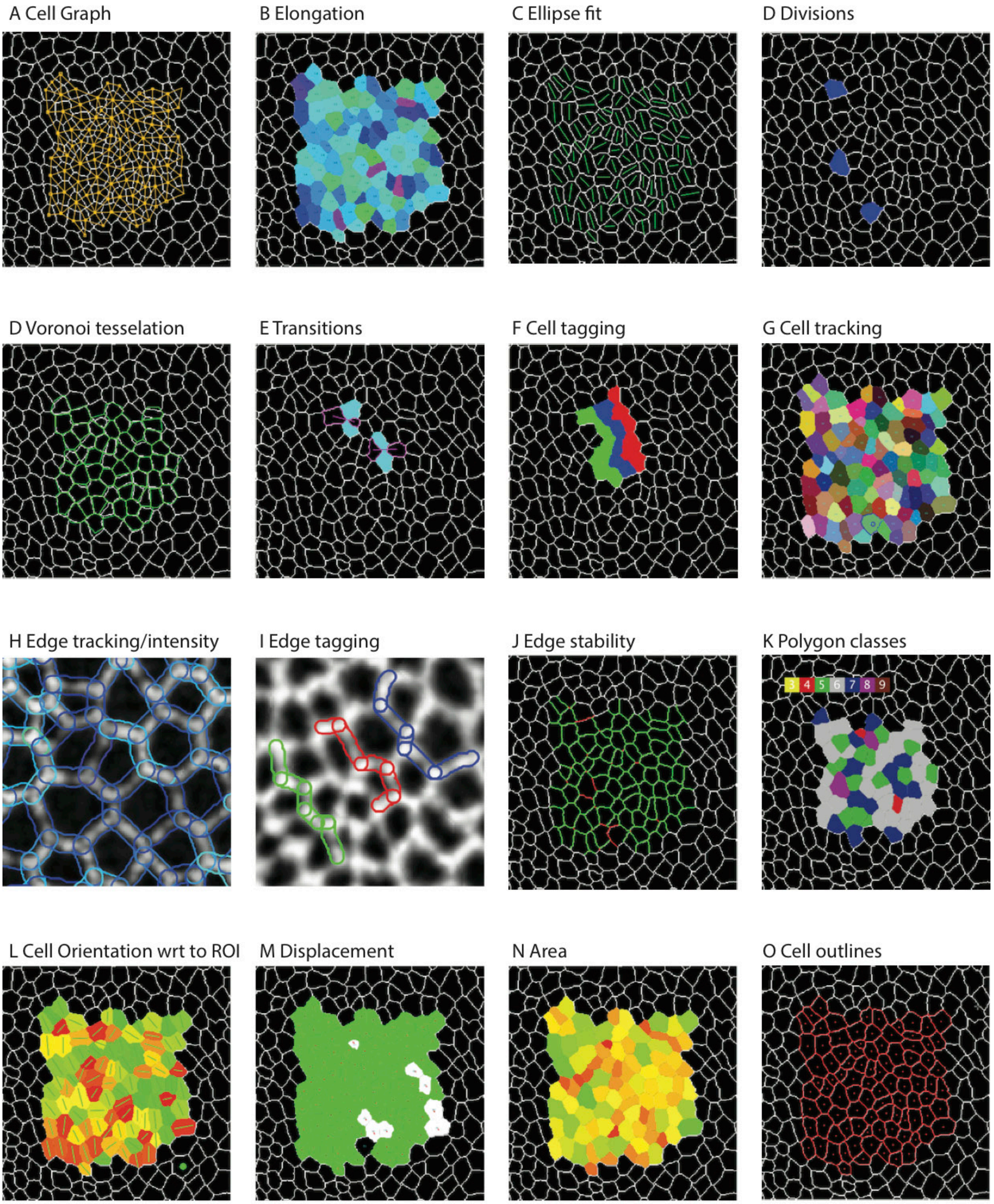

A Cell Graph
B Elongation
C Ellipse fit
D Divisions
D Voronoi tesselation
E Transitions
F Cell tagging
G Cell tracking
H Edge tracking/intensity
I Edge tagging
J Edge stability
K Polygon classes
3 4 5 6 7 8 9
L Cell Orientation wrt to ROI
M Displacement
N Area
O Cell outlines

Figure S3

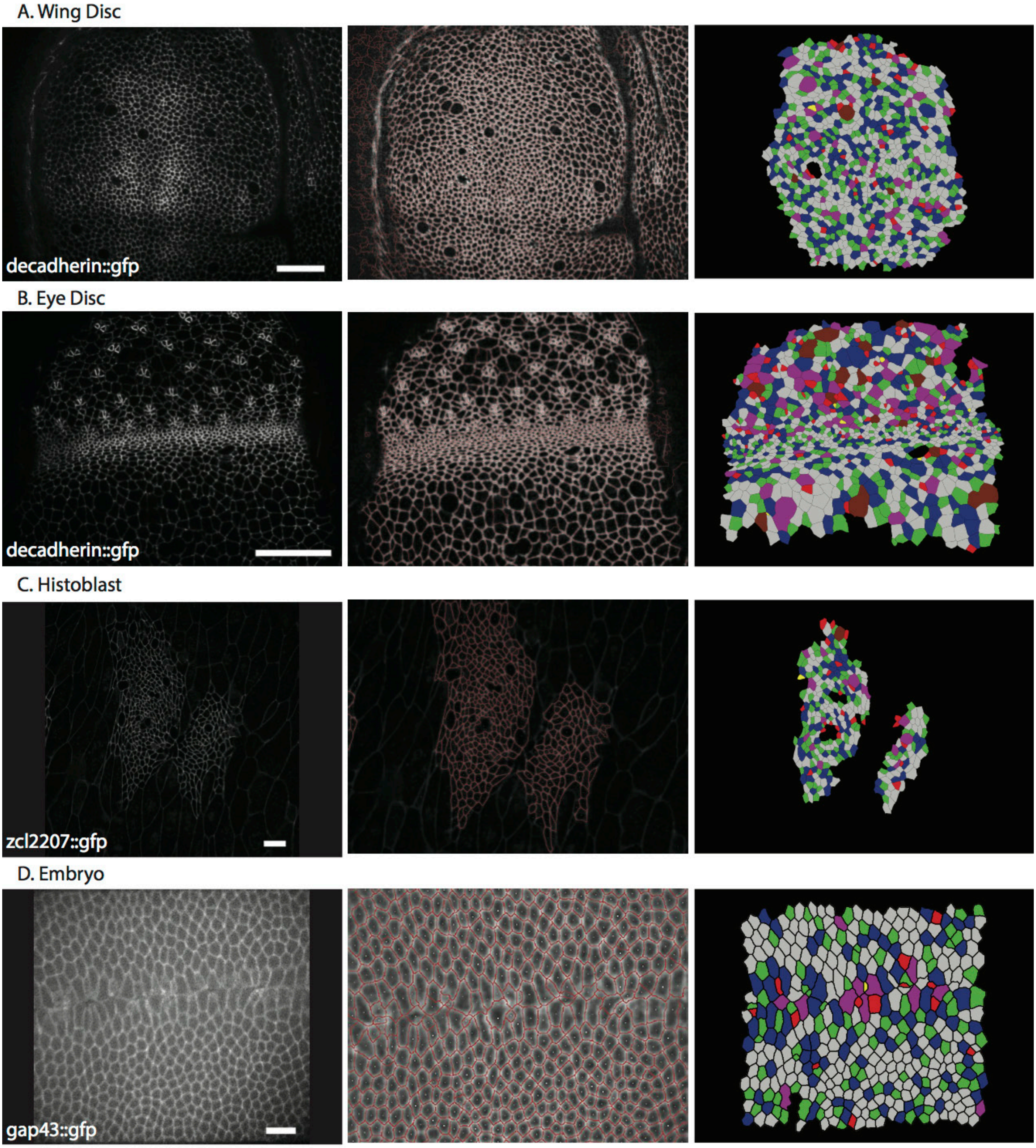

A. Wing Disc
decadherin::gfp
B. Eye Disc
decadherin::gfp
C. Histoblast
zcl2207::gfp
D. Embryo
gap43::gfp

Figure S4

A

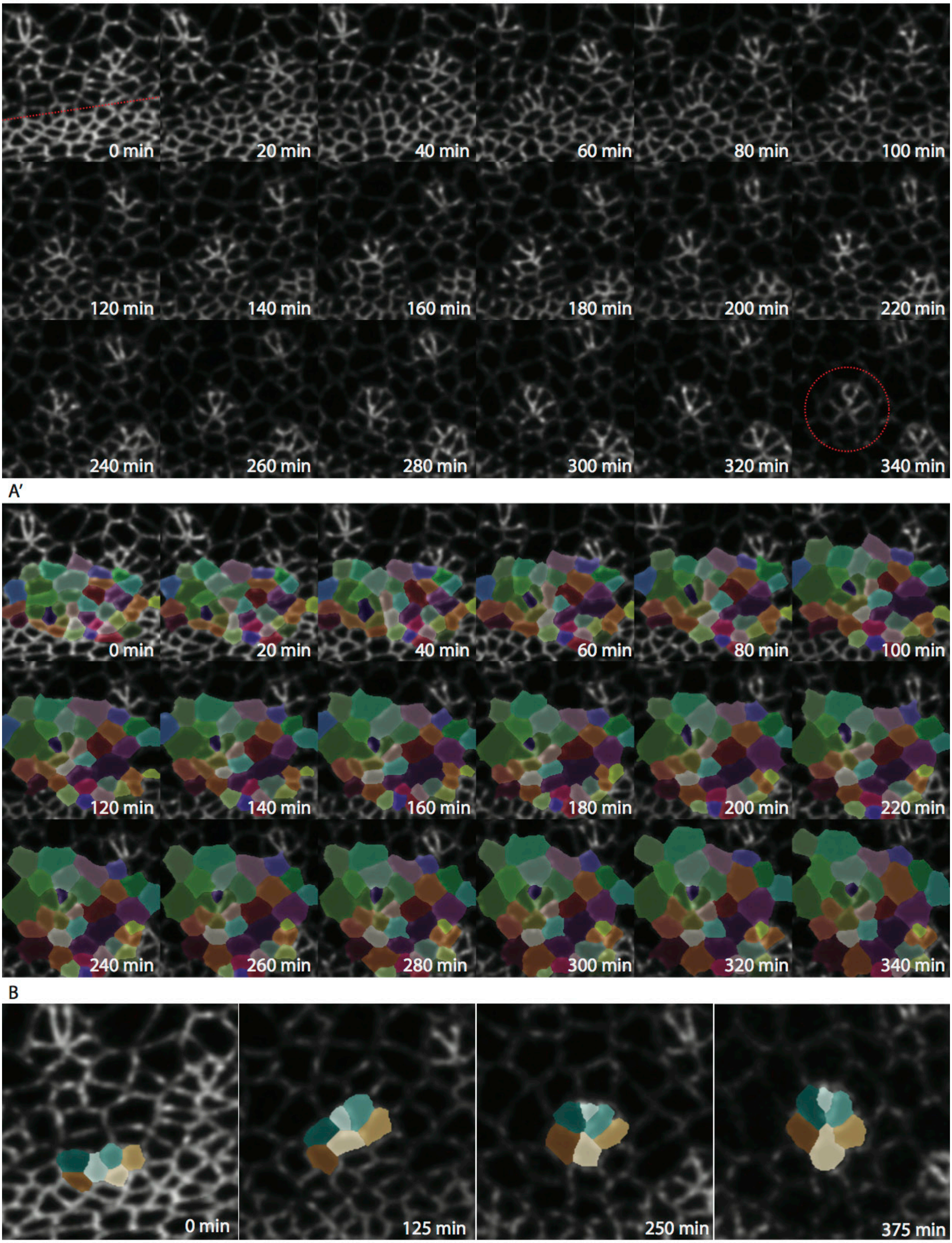
0 min
20 min
40 min
60 min
80 min
100 min
120 min
140 min
160 min
180 min
200 min
220 min
240 min
260 min
280 min
300 min
320 min
340 min
A'
0 min
20 min
40 min
60 min
80 min
100 min
120 min
140 min
160 min
180 min
200 min
220 min
240 min
260 min
280 min
300 min
320 min
340 min
B
0 min
125 min
250 min
375 min

Figure S5

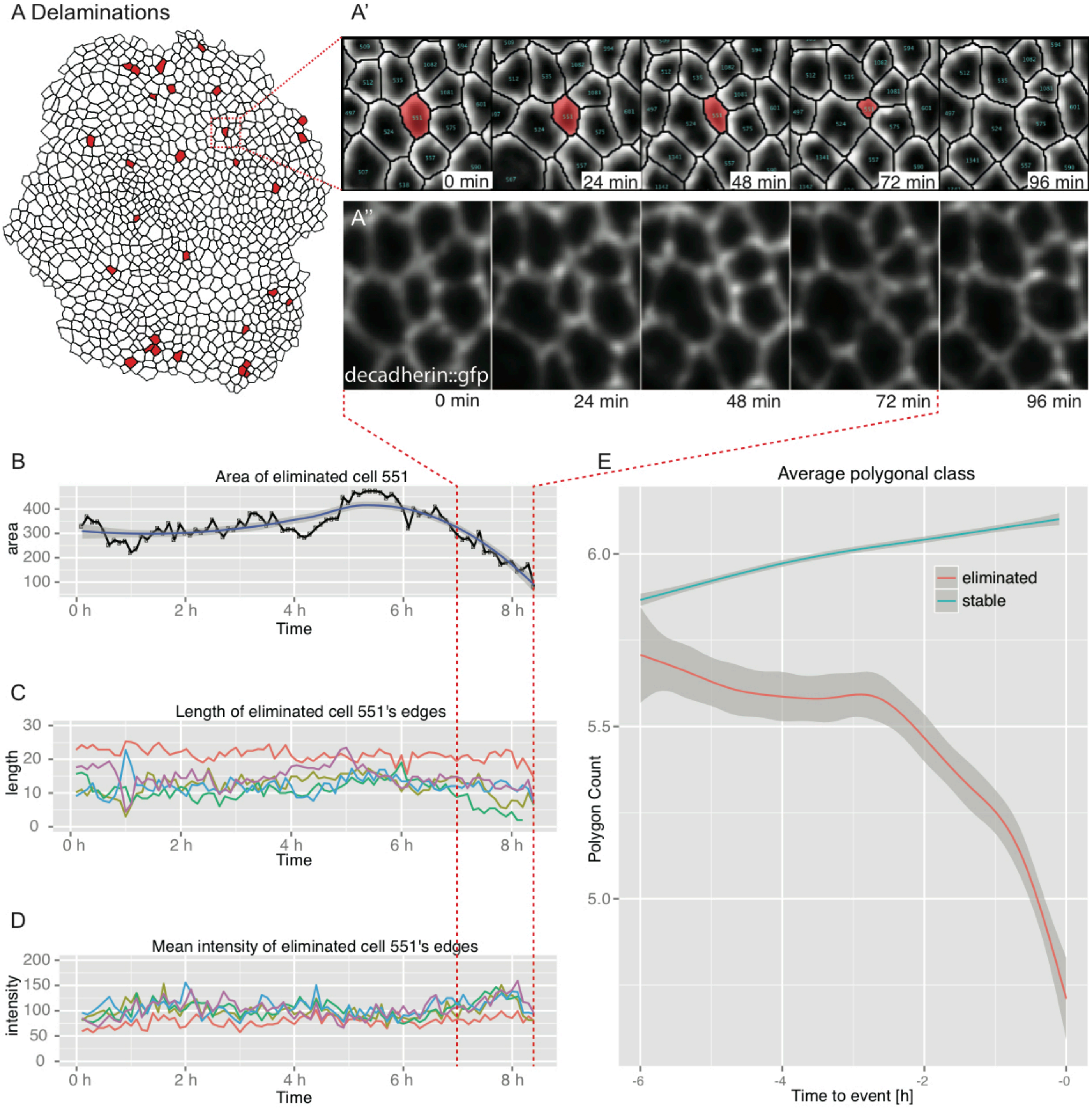
A Delaminations
A'
0 min
24 min
48 min
72 min
96 min
A''
decadherin::gfp
0 min
24 min
48 min
72 min
96 min
B
Area of eliminated cell 551
area
400
300
200
100
0 h
2 h
4 h
6 h
8 h
Time
C
Length of eliminated cell 551's edges
length
30
20
10
0
0 h
2 h
4 h
6 h
8 h
Time
D
Mean intensity of eliminated cell 551's edges
intensity
200
150
100
50
0
0 h
2 h
4 h
6 h
8 h
Time
E
Average polygonal class
Polygon Count
6.0
5.5
5.0
eliminated
stable
-6
-4
-2
-0
Time to event [h]

Figure S6

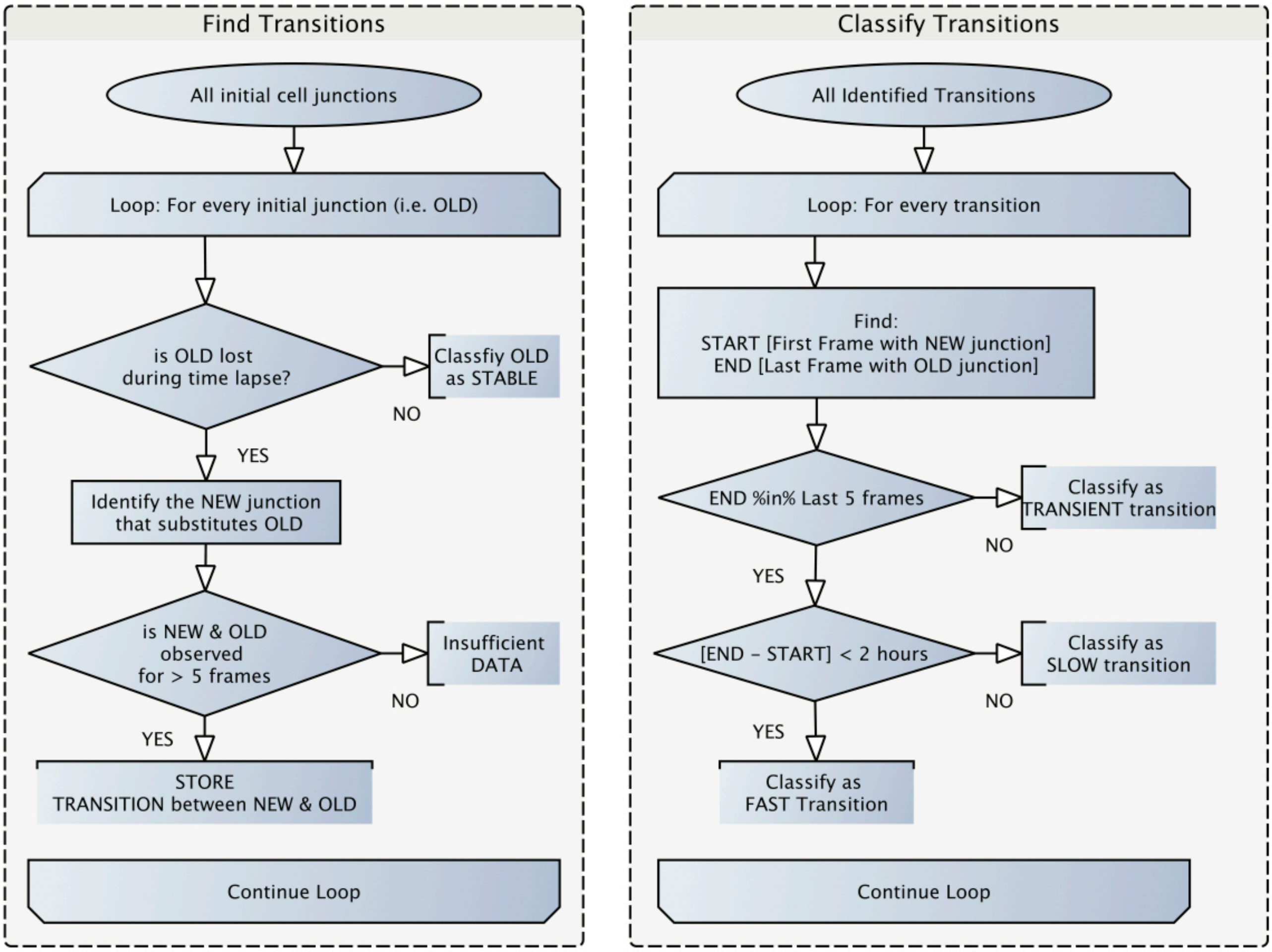

Find Transitions
All initial cell junctions
Loop: For every initial junction (i.e. OLD)
is OLD lost
during time lapse?
Classfiy OLD
as STABLE
NO
YES
Identify the NEW junction
that substitutes OLD
is NEW & OLD
observed
for > 5 frames
Insufficient
DATA
NO
YES
STORE
TRANSITION between NEW & OLD
Continue Loop
Classify Transitions
All Identified Transitions
Loop: For every transition
Find:
START [First Frame with NEW junction]
END [Last Frame with OLD junction]
END %in% Last 5 frames
Classify as
TRANSIENT transition
NO
YES
[END - START] < 2 hours
Classify as
SLOW transition
NO
YES
Classify as
FAST Transition
Continue Loop

Figure S1, related to Figure 3. EpiTools Complete workflow

(A) Flowchart describing the complete processing workflow for a 3D time lapse data set. First the Selective Plane Projection is applied to project individual time points on a 2D plane without interference from other membranes (e.g. the peripodial membrane in the imaginal wing discs). Second, the 2D image sequence is registered to correct for sample movement during the acquisition. Third, Contrast-Limited-Adaptive-Histogram-Equalization (CLAHE) is applied to even the intensity distribution across the image. Fourth, cells are localized through smart seed point identification to optimally initiate the segmentation step. Fifth, the segmentation procedure identifies the cell geometries through the region growing algorithm initiated from the seed locations. Six, the user can semi-automatically correct the segmentation result by placing or removing seed. The seeds are automatically connected through time to give feedback. If corrections were applied a Re-segmentation step (not shown) will integrate the modifications into the final segmentation. The results of the first part are the outlines of the cells, also called skeleton images.

The skeletonized time points are the input for the EpiTools plugins for the bioimaging platform Icy. First, the polygons representing each cell are identified and connected in space, i.e. a cell graph of neighborhood connectivity. Temporal linkage is added by graph based cell tracking, extending the previous data structure into a spatio-temporal graph. The user is still given possibility to correct the image skeletons by manually adding or removing cell edges through the CellEditor plugin. The curated Cell Graph is finally used to project informative overlays on the original image and/or derivatives, as well as exporting data for quantitative analysis.

(B) 3D Surface generated by the CellSurface plugin (part of the EpiTools plugins for Icy). EpiTools for Matlab saves a height map when the user applies the selective plane projection module. The map corresponds to the second, more detailed, surface estimation to locate the signal of interest. Files are saved within the analysis folder (/vtk). These exported files (.vtk) can be read by the CellSurface plugin, part of the EpiTools plugins for Icy. CellSurface generates a 3D Surface ROI leveraging on the 3D Mesh ROI plugin by Alexander Dufour (Dufour) and the VTK library http://www.vtk.org.

(B') Projection overlay, available through the CellOverlay plugin, visualizes as a color gradient the magnitude of the z-component from the surface normal of every cell. The overlay uses the estimated height map (see legend S1B) to assign z-coordinates to all coordinates of the cell's polygon. To compute the approximated 3D surface normal for every cell we use Newell's method (Ivan et al., 1974). The z-component of the surface normal is useful to estimate the area projection bias through the proportional dependence between 3D and projected 2D area (John and Alan, 1987). All values can be exported in an excel sheet through the layer options menu in Icy.

Figure S2, related to Figure 4. EpiTools Icy CellOverlay examples

Examples of the different types of data overlays available upon the release of EpiTools. More complete descriptions can be found on the EpiTools website.

Figure S3, related to Figure 2. Different epithelia analyzed using EpiTools.

Scale bar in all images are 20µm. (A) *Drosophila* wing imaginal disc labeled with Ecadherin, imaged with spinning disc microscopy. (B) *Drosophila* eye imaginal disc labeled with Ecadherin, imaged with spinning disc microscopy. (C) *Drosophila* histoblast nests labeled with ZCL2207 (ATPalpha subunit, septate junction marker), imaged with laser scanning confocal microscopy. (D) *Drosophila* embryo labeled with GAP43, imaged with spinning disc microscopy.

Figure S4, related to Figure 6. Ommatidial cell rearrangements in *Drosophila* eye imaginal disc

(A)Time-lapse of *ex vivo* imaging of ommatidial cell rearrangements during the development of the eye imaginal disc. After the morphogenetic furrow (red dashed line) passes, the ommatidial cells start differentiating into preclusters (inside the dashed red line).

(A') Epitools can be successfully employed to segment and track the differentiation of ommatidial cells.

(B) The tracking information can be used to follow cell rearrangements and trajectories during ommatidial differentiation.

Figure S5, related to Figure 5. Cell delaminations in the *Drosophila* wing disc

(A) Spatial distribution of live cell delaminations occurring over a 10 hour time period, *ex vivo*. A' Representative montage of a live cell delamination detected by Epitools. A'' The ecadherin:gfp signal remains constant throughout the process, a hallmark of live cell delamination.

(B) The area of cell 551 fluctuates randomly prior to the delamination.

(C) Epitools can track the evolution of the length of each edge of cell 551 prior to its delamination. There are no clear trends.
(D) The intensity of the ecadherin signal of each edge of cell 551 remains relatively constant until the delamination event.
(E) On average, delaminated cells see a decrease in their number of neighbors prior to being delaminated.

Figure S6, related to Figure 7. Flowchart for the identification and classification of T1 transitions in the wing disc samples.
The procedure is divided in two major parts. The first (Find Transitions) concerns the task of finding the transitions by analyzing the junctions of all cells. The second (Classify Transitions) classifies the identified transitions by analyzing the duration and persistence of the rearrangement.

Find Transitions – Step 1
Consider the set of all junctions in the first frame of the sample. For every edge the algorithm verifies whether the edge was lost at any point in time. If the edge is conserved throughout every time point it is classified as STABLE junction and not further analyzed. The remaining junctions are unstable and have thus at least 1 time point where they are not present. The algorithm identifies the edge that substituted the old edge by looking at the change in neighborhood relationships. If the change is preserved for more than 5 frames (threshold can be changed in the graphical user interface) the algorithm classifies the TRANSITION. A persistence with fewer than the set threshold is classified as INSUFFICENT to call a T1 transition.

Classify transitions – Step 2
Consider the set of all identified/found transitions by step 1. For every transition the algorithm identifies two variables: START, the first frame in which the new edge appeared, i.e. the edge that substituted the initial edge (old). END, the last frame in which the old edge is observed. Using these two variables we will classify the type of transition. If the frame END is one of the last 5 frames the transition is considered to be TRANSIENT. This decision is based on the idea that a stable transition should be observed for a sufficiently long time without reverting to the initial condition (old edge). The second condition is used to classify stable transitions according to how much time passed between the frame START and the frame END. The interpretation of this duration can be seen as the amount of time to stabilize the transition. We identified a threshold of 2 hours to define whether a transition should be defined FAST or SLOW.

Supplemental Tables

Table S1, related to Figure 1. Comparison of existing image segmentation and tracking software

Software	Language	First Tissue used	Dim	Open source	Setup	ImageJ Icy interface	Last update	Citation
Packing Analyzer	Java	DM Wing	2D	No	Easy	No	2012 /11	(Aigouy et al., 2010)
Seed-Water-Segmenter	Python	DM Amnio-serosa	2D	Yes	Medium	No	2015 /08	(Mashburn et al., 2012)
EDGE	Matlab Java	DM Embryo	3D	Yes	Medium	No	2012 /08	(Gelbart et al., 2012)
EDGE4D	C++	DM Embryo	3D	Yes	Advanced	No	2014 /03	(Khan et al., 2014)
TTT	C++	DM Notum	3D	Yes	Advanced	No	2015 (incom plete)	(Cilla et al., 2015)
Morpho-GraphX	C++ Cuda	AT Meristem	2.5D	Yes	Hardware required	No	2015 (contin uos)	(Kierzkowski et al., 2012)
Ilastik	Python	Machine Learning	2D/3 D	Yes	Easy	Cell Profiler	2015 (contin uos)	(Sommer et al., 2011)

Supplemental Experimental Procedures

Source Code

Source code for the current version of EpiTools is provided in SourceCode.zip. For updated versions please visit our website http://tiny.uzh.ch/dm and git repository https://bitbucket.org/davideheller/epitools/.

EpiTools Part 1a: Matlab and C++ modules

The image processing pipeline consists of a selective plane projection, image registration, automatic seed point generation, region growing segmentation from seed points (which conceptually represent cell centres), seed tracking and correction, followed by the final cell boundary segmentation. A detailed description of these steps can be found below.

Selective plane projection

Since many epithelial tissues grow as a curved surface, such as a dome in the case of the wing imaginal disc (Legoff et al., 2013), a simple maximum intensity projection along the z-axis of a confocal microscopy stack would capture any unwanted signals (such as the peripodial membrane in the wing disc) situated in a different focal plane (Fig. 2A). This is a typical problem with whole mount preparations. It was thus necessary to develop a projection method designed to follow the curvature of the tissue by fitting a surface to the epithelial topology. This was achieved through a two-stage projection approach. First a surface mesh was extracted from the image volume by fitting and interpolating scattered feature points (D'Errico, 2006), defined as voxels with strong image intensity. Although most feature points belonged to the desired layer's mesh, some of these points were 'outliers' and referred to signals in unwanted planes. Increasing the stiffness of the fitted surface would settle on the denser mesh of the desired layer, and thus remove most of the outliers. Only feature points with a strong intensity were used for this first step. In a second step, feature points that have a large deviation from the computed surface were removed. The surface fitting is repeated and can now be achieved with less stiffness and thus follow the curvature more accurately (Fig. 2A, green line). Finally, pixel intensities along the fitted surface were obtained which formed the projected image on which the subsequent image segmentation was performed. The 3D surface fit can be exported and used to correct subsequent geometric analyses, if necessary (Fig. S1).

Registration

Images are aligned (registered) at different time points using the image intensity distribution. Images were processed with a rigid transformation to find an optimal correlation between frames. The image registration step can also be performed through external software such as the StackReg plugin for ImageJ (Thevenaz et al., 1998) controlled via EpiTools using the MIJ interface (Sage, 2012).

Smart seeding

Using the selective plane projected image, the aim of this step is ideally to create a single seed point per cell from which to grow cellular regions (Fig. 2B). The use of seed points as a simple handle for each cell allows for very efficient adjustments. A single mouse click is enough to add, remove or fuse cellular regions. As long as the correct seeds are used, the final region-growing stage provides a sufficiently good segmentation result (Fig. 2C) and thus emphasizes the importance to generate accurate seed points during the initial stage (Fig. 2B). Seed points are often determined as the centroids of small homogeneous low signal regions. However, such an approach often leads to multiple seed points per cell (Fig. 2B, magenta arrows) as cells could contain several different homogeneous regions within its boundaries. Our seed point generation method was thus devised to include growing and merging of regions to reduce fragmentation: Homogeneous regions of a certain size below a rising signal intensity threshold (cell boundary signal) are identified and allocated to become new cellular regions with a unique identification (ID). Existing regions are grown by assimilating unallocated neighbouring pixels below a given intensity threshold. Each region is represented by a cluster of pixels with the same ID. While growing, neighbouring regions of small sizes and low intensities are merged to form larger regions. This approach ultimately provides images with, in most cases, just one seed point per cell (Fig. 2B, green arrows).

The initial seed finding could still produce multiple seed points especially in dividing cells due to the considerable increase in cell size in M phase. To reduce this duplication, an additional region-merging step was introduced. In this method, the intensities at the borders of adjacent regions were evaluated. Usually, the intensities of the borders of regions would be higher than the intensities in the centre of regions. In case of falsely identified borders, as often observed within large dividing cells, the border intensities of two adjacent regions would be similar or lower than the centre intensity of the region. Pixels are identified as similar or lower if their intensity is less than the intensity of the region's centre plus half its standard deviation. The percentage of such pixels within each border region is calculated and then used as a merging criterion. Finally, new seed points are calculated as centroid positions of merged regions (Fig. 2B).

Region growing segmentation

Once the seed points have been obtained, the cell region growing step is performed to provide accurate cell shape representations that precisely follow the contours of cell junctions (or membrane), as opposed to using linear approximations (Cilla et al., 2015). This is important because an accurate representation of the curvature of cell junctions is critical for understanding the mechanical properties of the cells (Brodland et al., 2014). The projection image is smoothed with a Gaussian kernel to reduce signal discontinuities at cell junctions. Cellular regions are grown from the seed points by assimilating neighbouring pixels below an increasing intensity threshold. The region growing is performed in parallel for each seed point and is guided by the local intensity flow, climbing up the intensity gradients that separate cells. This concurrent approach, whereby regions are grown at the same time in parallel, ensures that expanding regions meet at the ridge between cells where the cell junction is located. The growing is stopped locally once pixels from adjacent regions touch, which prevents spilling over or racing along the cell ridge resulting in discontinuities in the segmented cell boundaries. This combination of concurrent growing and local control results in an improved segmentation that more accurately follows the membrane contours, compared to the MATLAB watershed technique as shown in (Fig. 2C; (Meyer, 1994). This region growing approach can be re-applied after all seed corrections have been performed, at the final re-segmentation step, to provide an optimal segmentation.

Seed tracking and seed error correction

Seed tracking has been implemented primarily to facilitate the semi-automatic seed correction procedure. The tracking is performed on the cell seed positions obtained from the above segmentation steps. For each cellular position, a corresponding position is found in the vicinity in the previous frame (the vicinity parameter is expressed in pixel squared area around the seed). A missing seed point is identified by a broken track (Fig. 2D, magenta track) often due to segmentation errors, which are easily rectified and subsequently re-segmented. Each new track is allocated a track ID. The search space is initially small as the algorithm tries to find a corresponding position in the previous frame that has not moved far. The search space is increased at the next iteration to accommodate larger movements while excluding already allocated positions. The length of each track is recorded. The tracking has been combined with an innovative GUI to identify broken tracks and to add or delete seed points manually while continuing the tracking process. Cell division events can later be identified from broken tracks. A final re-segmentation can be applied on the corrected seed points to produce a final series of segmented frames, which can be exported as skeletons that accurately represent cell junction (or membrane) signals.

EpiTools Part 1b: EpiTools-Matlab-GUI

1. Component based software engineering

Component based software engineering. Component-based development EpiTools framework has been designed in compliance with component based software engineering (CBSE) standards. Therefore, we built a kernel of drivers which provide state communication layers, data retrieving methods, and dynamic result storage. Drivers are connected and assembled as modular stacks. Whether an element in the stack initiate a unilateral communication with a higher element in the stack configuration, an abstraction layer covers the element lower in the stack disposition. Layer abstraction increases flexibility in stack calls and data i/o. Analysis modules wrap multiple software components. Interaction between analysis modules and software framework is established via drivers which regulate, prioritise and manage connection to lower layers (i.e. data transportation between modules is realised via a spool list system which keeps track of the analysis modules successfully executed and the objects in output from the executed modules). Successful execution of an analysis module results both in objects creation (i.e. projected surface image files) and in tag exportation into the software instance

environment which is used as availability reference by successive analysis module execution. This system protects results retrieved from overwriting in case of multiple runs. Module execution is monitored by an executor driver which records both execution meta-data and parameter settings used for results retrieving. Parameter and meta-data storing enhances results reproducibility and error handling power.

2. Centralized computing

Client-Server Framework. Computational requests are elaborated through a first-in first-out (FIFO) en-queueing and de-queueing process. Every single command requested by the user or automatically generated by the software is first appended at the end of the execution list while submission to the process launcher will submit the first at the front of the execution list. This group of classes and methods composes the integrated queue & spool system which is responsible for the command execution, output object creation, tag exporting and environment releasing. Successfully executed analysis modules drive results objects (i.e. image files, text files or xml meta-data files) creation which is regulated by sandbox and storage protection drivers. These two subsystems link the physical location of created files with the program session in execution. File creation and tag exportation trigger a specific set of events which are listened by a class associated event manager. This events collecting system is linked to lower software layers through a communication channel initiated by the associated class which triggered the event (i.e. event-related procedure are required for synchronising and linking output objects with their availability status during EpiTools instance execution).

3. Extension interfaces

Input/Output drivers EpiTools framework interfaces other software platforms exporting output objects via standard and open file formats. This increases results portability and flexibility while it allows for integrating it in much bigger pipelines without intermediate file conversion steps. Importing and exporting processes are realised using OME Bio- Formats libraries (Linkert et al., 2010) linked to EpiTools through a Matlab wrapper provided by Open Microscopy Environment and custom adapted in our software implementation. User setting file, analysis descriptor files and meta-data files are written and read in clear-text XML format.

4. Graphical user interface

GUI Interface EpiTools comes with an elaborated GUI interfaces which enables the user to interact directly with the framework, modify the analysis execution and manage the import/export process of images, meta-data and results. Users without coding experience are then easily guided through the software through auto-explanatory forms with assisted and personalised help functions. EpiTools GUI is specifically designed to overcome definite processing requests - i.e. manual seed tracking or image cropping. Non-GUI functions benefit of EpiTools GUI interface since they gain interactivity and graphical display methods inherited from the generic graphical framework. Main forms are generated using Matlab GUI libraries which consists of code wrappers around Java Swing GUI libraries. Extensions of the built-in components have been realised via direct access to Java Swing libraries and JIDE classes (i.e. JTree, JTable, JTreeTable). Implementing the connection to these libraries required Matlab Java wrappers which have been adapted for a more fluid graphic user experience. EpiTools GUI interface is completely portable on all the operative systems where a Java Virtual Machine is deployable, albeit GUI components appearance may differs due to operative system rendering libraries.

EpiTools plugins for Icy

1. Graph creation: spatial and temporal linking

The graph generation algorithm included in CellGraph starts by analyzing the input skeletons to identify the individual polygons representing each cell. We use the java library jts (java topology suite (JTS, 2015) to extract and store the detailed polygonal shape of every cell. Through polygon intersection we discover spatial neighborhood relationships and subsequently store them in a graph structure using the jgraphT library (jGraphT, 2015). To add the temporal linkages we developed a graph-matching algorithm that connects cells from one frame to the next. First we compute a score between cell pairs in different frames according to the cell's polygon overlap and the distance between their centroids. Second, we determine the optimal match between cells with a stable marriage algorithm (Gale and Shapley, 1962). This algorithm determines the best reciprocal match based on the multiple candidates that every cell received from previous frames. Third, we analyze the unmatched cells by heuristics to identify divisions, eliminations or suggest a segmentation mistake. The completed data

structure is stored in memory (i.e. Icy's swimming pool) for subsequent use by one of the other plugins.

2. Exporting the data contained in the spatio-temporal Graph
As stated in the main text, we offer two main export methods. First, from every overlay excel sheets can be generated containing the visualized quantities. To do this it is sufficient that the user clicks on the corresponding overlay in the Icy Layer menu and selects 'Export data to excel' from the options panel below. Second, the use of the CellExport plugin. Here multiple options are available: (1) a larger and more exhaustive excel sheet that contains gathered information from many overlays; (2) an XML based graph format, called GraphML (Brandes et al., 2002), which stores the neighborhood relationships of the cells. GraphML files can be read by many scripting languages such as R or Python. An example analysis file can be downloaded from the project homepage (http://tiny.uzh.ch/dP); (3) a PDF export option is available to generate vector graphics from the overlay using. For this feature we use the gnujpdf library (Beard, 2001); (4) Skeleton export options for faster loading times using the Well-Known-Text format (WKT, https://en.wikipedia.org/wiki/Well-known_text) ; (5) Tracking (if applied) can be exported as csv reducing further loading times.

Supported file formats

1. EpiTools for Matlab – input file formats

We support 8 or 16 bit grey-scale images and use the OME bioformats library for Matlab (Linkert et al., 2010) to import files. While the latter guarantees access to almost all commonly employed microscopy image formats, we currently pose two further requirements: (1) individual files have to contain all information of a time point, i.e. it is not possible to combine multiple files into a single time point. We require this to efficiently concatenate multiple time points; (2) we have limited the possible image file extensions to the following subset to allow fast file inspection: czi, zvi, cxd, ome, ome.tiff, mrc, tif, tiff, lif, lei, ipl, raw, ics, ids, bmp, png, pic, mvd2. It is possible to extend the selection upon request. Preferred image format: Individual TIFF files for every time point. For a simple image format conversion we recommend icy and imageJ, both programs offer very intuitive reformatting options.

2. EpiTools plugins for icy – skeleton file format

Skeleton files are assumed to be 8-bit binary representations of membrane signal where higher values represent membrane signal and lower values background. To ensure 8-connectivity, the imagej function "skeletonize" is applied to every input image. Preferred image format: 8-bit Tiff.

Quick Guides for EpiTools

Disclaimer: This is a static version of the guides present on our website, please consider visiting the website for an up-to-date version which also features images and videos. Link: http://tiny.uzh.ch/dm

Quick-Guide to the EpiTools application for Matlab

EpiTools comes in a single software package for Matlab. We recommend Matlab version (2014a) since we developed and tested EpiTools on this version.

How to install it and receive our latest patches or updates

From the home page of EpiTools you can easily download the latest available version of EpiTools.

1. Extract the package
2. From the directory generated by exploding the package, double click on the launcher file correspondent to your operative system. Done!

In case the launcher does not start (or fails with "EpiTools_mac: Permission denied"), follow these instructions from a new console windows (in OSX, open a new Terminal session) to allow the program launcher to be executed:

1. *cd [download & extraction location]*
2. *chmod +x EpiTools_mac*

How to create a new analysis

Any new analysis generated from EpiTools interface is the result of a guided process where the user fills all the required information via the GUI interface provided.

1. From the main EpiTools interface, click on *File>New Analysis*.
2. Specify the path where the analysis file will be saved.
3. Specify the path where the images have been stored (e.g. use our example files included)
4. Give a name to the analysis you are currently building. (version and department can be useful if you plan to share the analysis)
5. Now turn to the table sub-window, which should list the available images in the selected folder (for the example this will a single file) and
    a. Include the images you would like to analyze by selecting the tick box in the column titled “include?”
    b. To modify more cells at the same time: select the first cell, hold caps, select the last cell, write the new value, press enter
6. Finalize the analysis creation by clicking “Confirm and Proceed”

A side bar should now appear on the left part of the screen. The latter displays all the actions executed so far and is useful for revisiting the analysis (context-menus are available on each node)

Run modules

1. Select a module to run from *Action* menu
2. In case the module requires any additional settings, you will be prompted with a windows where you will be able to specified the required settings.

On analysis module completion, you will be able to visualize the results (if the module generates them) and you will see a new node in the *analysis workflow* tree.

Simple example analysis with included sample (8_bit_sample)

Create a new analysis file for the sample data set. Walk through the following set to produce the skeleton files to be used in EpiTools plugins for ICY. Detailed descriptions of all the modules and parameters are available on our website.

1. Select the *projection module* from the Action menu
    a. This creates a 2D projection from a Z-stack by selectively choosing from which plane to extract each pixel based on a surface estimation.
    b. Run it with default parameters
2. Select the *registration module* from the Action menu
    a. This corrects movement during the acquisition of time series by aligning successive frames to the first.
    b. Choose “StackReg” and Run
3. Select the *Clahe module* from the Action menu
    a. This applies the matlab command adapthisteq® to reduce contrast differences throughout the image
    b. Run it with the default parameters
4. Select the Segmentation module
    a. This detects the individual cells in the image and finds the boundaries with a seed based region growing algorithm.
    b. Run it with the default parameters
5. Select the Skeleton module
    a. This transforms the segmentation output into binary skeletons for every frame. A skeleton depicts the boundaries between cells with a white 1 pixel line (255) over black background (0). File format is PNG.
    b. Run it with default parameters

Done! You can now find the skeleton files in the analysis folder in your chosen "save location"

Additional features

- Correct the segmentation results through our assisted *Tracking Correction module* which allows you modify, add or eliminate seeds from which the Segmentation module discovers cell regions. Re-apply the segmentation on your modifications with the *Re-Segmentation module*
- Test different parameter settings for the same modules by taking advantage of the comparative mode (3rd icon from the right in the menu bar)
- Connect Matlab to icy for best visualization capabilities (2nd icon from the right; this requires the Matlab communicator plugin by Y Montagner to be installed in icy)

Quick-Guide to the EpiTools plugins for ICY

Welcome to the EpiTool plugins for ICY. This collection of plugins for the bioimaging platform icy allows to transform skeleton images into interactive overlays to explore and analyze your data.

Warning: if your icy background appears black instead of grey, the plugins will most likely be affected an unsolved memory leak. Most reported cases appear in co-occurrence with Apple Retina® screens. Temporary Fix: Use an external monitor with the macbook lid closed

Installation: Download & Move the files in the right place

1. Download the package cellGraph_beta.zip from our website (here)
2. Extract the package
3. Place the plugin ***folder davhelle*** into the icy plugin folder (e.g. programs/icy/plugins)
4. Place the workspace file ***EpiTools.xml*** into the icy workspace folder (e.g. programs/icy/workspace)
5. The ***folder test*** does not require any particular location

To update just replace the cellGraph_v#.#.#.jar in the plugin folder davhelle
Release information about latest cellgraph versions: CHANGELOG.TXT
Enable the EpiTools Workspace in Icy

In order to add the **EpiTools toolbar** to your icy installation
1. Enter the preferences (icy logo > preferences)
2. Go to *local workspace* menu
3. Enable the EpiTools workspace by checking the tickbox

Confirm by clicking *Ok*, you will be asked to restart icy to apply the changes.

Install the required plugins for icy

In order to run the EpiTools plugins, only one additional icy plugin is required, EzPlug. This is a plugin which facilitates the building of graphical user interfaces and is used by all EpiTools Plugins. To install it simply write in the icy search field the following plugin name [network connection required]:
- EzPlug SDK (most likely already installed)
- 3D Mesh ROI (only for the CellSurface plugin)

Test if the installation was successful

To test the installation:
1. Run the **TestLoader** Plugin in the EpiTools Bar/or through the search bar
2. You will be asked to locate the **folder test** from the download package
3. Shortly after a new viewer should be visualized (image below)

Generating overlays using CellOverlay

After installing the plugins and loading the test file we are now ready to generate overlays. In fact two overlay are already present if we look at the ***Layer*** menu in the icy menu on the right. By clicking on the eye icon on the side of each overlay you can toggle the presence.

Create a new overlay

Let's add a new overlay to the test viewer (click again on TestLoader if you closed it). Here we will add a graph view overlay which visualizes the neighborhood connectivity of every cell.

1. Open the CellOverlay plugin from the toolbar
2. Select *GRAPH VIEW* from the Overlay list
3. Generate the overlay by clicking *Add Overlay* on the plugin bottom

A new overlay is now present on the image and a new entry in the Layer menu called GraphEdges.

Adjusting a gradient color overlay

When the overlay displays a gradient through a color scheme like in the Area example the user can adjust the scheme through the 4 parameters in the OptionPanel in the Layer menu (see image below). The *Gradient Maximum* and *Gradient Minimum* parameters set the extremes of the gradient (everything above and below has constant color). The *Gradient Scale* is a multiplier between 0 and 1 to scale the color gradient into a particular range of colors (see HSV color scheme for more information about the color space). The *Gradient Shift* is complementary to the scale, shifting the scaled color gradient into the desired region by adding a constant factor between 0 and 1.

As simple exercise let's add the Area gradient and change colors from [Blue<>Magenta] to [Red<> Blue].

1. Select CELL_AREA from the Overlay list
2. Add the overlay
3. Navigate to the Layer menu and click on the CellArea layer to select it
4. You should now see the Options panel right at the bottom of the layer list
5. Change Scale from 0.5 to 0.7 and Shift from

Export data from an overlay

While we offer a more complete export functionality with the separate CellExport, we can generate an excel sheet (XLS) from every generated overlay. This option can be reached from the layer option menu (see above) where you can also adapt the opacity or delete the overlay. For example to exprot the area of cells from the CELL_AREA overlay do the following:

1. Click on the layer name to make the export option appear
2. Click on the export button and choose where to save the spreadsheet
3. The spreadsheets contains a separate sheet for every frame in the movie

Import your own data with the CellGraph plugin

While the CellOverlay plugin generates the Overlays, it requires a graph structure in the shared plugin memory (also called *swimming pool* in icy). Learn here how to transform your skeleton images into a graph structure using the CellGraph plugin.

Current restrictions:
- Only one graph at the time is allowed in memory.
- Time series require the following pattern: [name]_000.[ext]
- Individual skeleton files, i.e. multidimensional tiffs are not yet supported

1. Choose your Input files

Most importantly CellGraph needs files from which to extract the graph structure:

1. If your files are images/bitmaps the default *File type* 'SKELETON' is correct
   Alternative files are WKT & VKT, both text based
2. Choose your input file

If you want to load a time series select the *First time point*
Please notice the file name convention mentioned in the warning above
Set the number of *Time points to load*, to start it is recommended to try with 1 or 2

2. Review the secondary input options [optional]

In order to correctly analyze the input files CellGraph can be fine tuned for specific file types. By default CellGraph assumes to receive files which were directly exported from the EpiTools App for Matlab.

1. Click the *Packing Analyzer files* if you used the software Packing Analyzer v.2-8 by (Aigouy et al. (2010)) for segmentation
    a. In this scenario use the image as first file. Cell_Graph will use the /handCorrection.png as skeleton
2. Choose *Cut on border line* to exclude the most outer cell layer from the analysis. This is advisable in case of poor performance at the segmentation boundaries
3. Choose *Remove very small cells* to exclude cells below an area threshold automatically, e.g. avoid small segmentation artifacts to be recognized as cells

3. Choose if to track your skeletons

Tracking is only possible if a time series is given as input. The standard tracking *Algorithm* is currently *STABLE_MARRIAGE* (Try the *HUNGARIAN* type for a more robust algorithm to movement but also time consuming!)

Depending on the time series frequency and the segmentation quality it can be helpful to change the default paramenters of the tracking algorithm:

1. Change the *Propagation limit* to amplify(+)/reduce(-) the number of previous frames that participate in the assignment of the track of a cell.
    a. This is useful when the sample is subject to strong motion, a shorter limit will prove more effective
2. Change the *Cut N border lines in 1st frame* to track only a core region of the sample. This is useful if the border region segmentation is poor.

4. Choose the Destination

The extracted graph structures are always used together with a background image. This image can be any image compatible (x, y,t) with the skeleton images, i.e. the original raw image / projected_image / second channel ecc

1. Choose *Image to overay* from you active Sequences
    a. The plugin requires an open image!
2. Deselect *Use ICY-SwimmingPool* only to avoid overriding another structure in memory. This will associate a single tracking view to the selected image.
3. Select *Remove Previous Overlays* if the destination image already overlays which are not needed anymore

6. Run the plugin

To generate the graph click on the triangle button on the lower-left of the plugin.

- During the Execution the progress can be followed both in the status bar as well as in *Output* tab. After execution the first overlay will be displayed on the active sequence.

Generated Result

A. In case of a *Single frame* the found cell polygons with the centroids will be visualized (i.e. the *CELL OUTLINE* overlay)

B. In case of a *Tracked time series* cell the automatically generated overlay will be the *TRACKING* one. Every cell is outlined by an individual color preserved through time and a filled with one of the following colors in case of a tracking event:
    a. [red] cell missing in previous frame
    b. [yellow] cell missing in next frame
    c. [green] cell missing in previous & next
    d. [blue] cell dividing in next frame
    e. [magenta] brother cell missing
    f. [cyan] cell eliminated in next frame
    g. [gray] brother cell was eliminated

Now you can add any overlay by executing the *CellOverlay* plugin, export data with the *CellExport* plugin or Modify the skeletons with *CellEditor*. You can find dedicated tutorials to each of these plugins on our website.

Supplemental References

Beard, E. (2001). gnujpdf, sourceforge.net/projects/gnujpdf/.
Brandes, U., Eiglsperger, M., Herman, I., Himsolt, M., and Marshall, M.S. (2002). GraphML Progress Report Structural Layer Proposal. In Graph Drawing, P. Mutzel, M. Jünger, and S. Leipert, eds. (Springer Berlin Heidelberg), pp. 501-512.
Brodland, G.W., Veldhuis, J.H., Kim, S., Perrone, M., Mashburn, D., and Hutson, M.S. (2014). CellFIT: a cellular force-inference toolkit using curvilinear cell boundaries. In PLoS ONE, pp. e99116.
Cilla, R., Mechery, V., Hernandez De Madrid, B., Del Signore, S., Dotu, I., and Hatini, V. (2015). Segmentation and Tracking of Adherens Junctions in 3D for the Analysis of Epithelial Tissue Morphogenesis. In PLoS Comput Biol, pp. e1004124.
D'Errico, J. (2006). http://ch.mathworks.com/matlabcentral/fileexchange/8998-surface-fitting-using-gridfit.
Dufour, A. http://icy.bioimageanalysis.org/plugin/3D_Mesh_ROI.
Gale, D., and Shapley, L.S. (1962). College Admissions and the Stability of Marriage. The American Mathematical Monthly *69*, 9-15.
Ivan, E.S., Robert, F.S., and Robert, A.S. (1974). A Characterization of Ten Hidden-Surface Algorithms. ACM Comput Surv *6*, 1-55.
jGraphT (2015). a free Java graph library, jgrapht.org.
John, M.S., and Alan, H.B. (1987). Ray tracing complex models containing surface tessellations. In Proceedings of the 14th annual conference on Computer graphics and interactive techniques (ACM).
JTS (2015). JTS Topology Suite sourceforge.net/projects/jts-topo-suite/.
Legoff, L., Rouault, H., and Lecuit, T. (2013). A global pattern of mechanical stress polarizes cell divisions and cell shape in the growing Drosophila wing disc. In Development, pp. 4051-4059.
Linkert, M., Rueden, C.T., Allan, C., Burel, J.-M., Moore, W., Patterson, A., Loranger, B., Moore, J., Neves, C., MacDonald, D., *et al.* (2010). Metadata matters: access to image data in the real world. The Journal of cell biology *189*, 777-782.
Meyer, F. (1994). Topographic distance and watershed lines. Signal Processing *38*, 113-125.
Sage, D. (2012). MIJ: Making Interoperability Between ImageJ and Matlab Possible http://imagejconf.tudor.lu/program/poster/makinginter.
Thevenaz, P., Ruttimann, U.E., and Unser, M. (1998). A pyramid approach to subpixel registration based on intensity. IEEE transactions on image processing : a publication of the IEEE Signal Processing Society *7*, 27-41.
