# Supplementary figures and images for: EpiTools: An Open-Source Image Analysis Toolkit for Quantifying Epithelial Growth Dynamics (part 2 of 2)
Source: Dev Cell. 2016 Jan 11;36(1):103–16. doi: 10.1016/j.devcel.2015.12.012 (PMC4712040; doi:10.1016/j.devcel.2015.12.012)

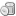

Supplement: Data S1. Source Code [file mmc2.zip › sourcecode/epitools_part1_matlab_v2.1.6/src/images/gif/bin_empty.gif]

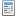

Supplement: Data S1. Source Code [file mmc2.zip › sourcecode/epitools_part1_matlab_v2.1.6/src/images/gif/blog.gif]

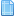

Supplement: Data S1. Source Code [file mmc2.zip › sourcecode/epitools_part1_matlab_v2.1.6/src/images/gif/blueprint.gif]

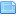

Supplement: Data S1. Source Code [file mmc2.zip › sourcecode/epitools_part1_matlab_v2.1.6/src/images/gif/blueprint_horizontal.gif]

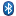

Supplement: Data S1. Source Code [file mmc2.zip › sourcecode/epitools_part1_matlab_v2.1.6/src/images/gif/bluetooth.gif]

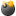

Supplement: Data S1. Source Code [file mmc2.zip › sourcecode/epitools_part1_matlab_v2.1.6/src/images/gif/bomb.gif]

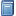

Supplement: Data S1. Source Code [file mmc2.zip › sourcecode/epitools_part1_matlab_v2.1.6/src/images/gif/book.gif]

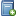

Supplement: Data S1. Source Code [file mmc2.zip › sourcecode/epitools_part1_matlab_v2.1.6/src/images/gif/book_add.gif]

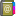

Supplement: Data S1. Source Code [file mmc2.zip › sourcecode/epitools_part1_matlab_v2.1.6/src/images/gif/book_addresses.gif]

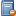

Supplement: Data S1. Source Code [file mmc2.zip › sourcecode/epitools_part1_matlab_v2.1.6/src/images/gif/book_delete.gif]

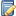

Supplement: Data S1. Source Code [file mmc2.zip › sourcecode/epitools_part1_matlab_v2.1.6/src/images/gif/book_edit.gif]

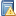

Supplement: Data S1. Source Code [file mmc2.zip › sourcecode/epitools_part1_matlab_v2.1.6/src/images/gif/book_error.gif]

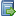

Supplement: Data S1. Source Code [file mmc2.zip › sourcecode/epitools_part1_matlab_v2.1.6/src/images/gif/book_go.gif]

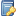

Supplement: Data S1. Source Code [file mmc2.zip › sourcecode/epitools_part1_matlab_v2.1.6/src/images/gif/book_key.gif]

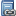

Supplement: Data S1. Source Code [file mmc2.zip › sourcecode/epitools_part1_matlab_v2.1.6/src/images/gif/book_link.gif]

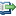

Supplement: Data S1. Source Code [file mmc2.zip › sourcecode/epitools_part1_matlab_v2.1.6/src/images/gif/book_next.gif]

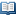

Supplement: Data S1. Source Code [file mmc2.zip › sourcecode/epitools_part1_matlab_v2.1.6/src/images/gif/book_open.gif]

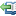

Supplement: Data S1. Source Code [file mmc2.zip › sourcecode/epitools_part1_matlab_v2.1.6/src/images/gif/book_previous.gif]

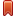

Supplement: Data S1. Source Code [file mmc2.zip › sourcecode/epitools_part1_matlab_v2.1.6/src/images/gif/bookmark.gif]

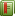

Supplement: Data S1. Source Code [file mmc2.zip › sourcecode/epitools_part1_matlab_v2.1.6/src/images/gif/bookmark_book.gif]

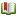

Supplement: Data S1. Source Code [file mmc2.zip › sourcecode/epitools_part1_matlab_v2.1.6/src/images/gif/bookmark_book_open.gif]

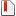

Supplement: Data S1. Source Code [file mmc2.zip › sourcecode/epitools_part1_matlab_v2.1.6/src/images/gif/bookmark_document.gif]

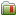

Supplement: Data S1. Source Code [file mmc2.zip › sourcecode/epitools_part1_matlab_v2.1.6/src/images/gif/bookmark_folder.gif]

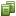

Supplement: Data S1. Source Code [file mmc2.zip › sourcecode/epitools_part1_matlab_v2.1.6/src/images/gif/books.gif]

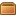

Supplement: Data S1. Source Code [file mmc2.zip › sourcecode/epitools_part1_matlab_v2.1.6/src/images/gif/box.gif]

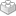

Supplement: Data S1. Source Code [file mmc2.zip › sourcecode/epitools_part1_matlab_v2.1.6/src/images/gif/brick.gif]

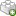

Supplement: Data S1. Source Code [file mmc2.zip › sourcecode/epitools_part1_matlab_v2.1.6/src/images/gif/brick_add.gif]

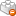

Supplement: Data S1. Source Code [file mmc2.zip › sourcecode/epitools_part1_matlab_v2.1.6/src/images/gif/brick_delete.gif]

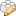

Supplement: Data S1. Source Code [file mmc2.zip › sourcecode/epitools_part1_matlab_v2.1.6/src/images/gif/brick_edit.gif]

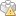

Supplement: Data S1. Source Code [file mmc2.zip › sourcecode/epitools_part1_matlab_v2.1.6/src/images/gif/brick_error.gif]

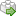

Supplement: Data S1. Source Code [file mmc2.zip › sourcecode/epitools_part1_matlab_v2.1.6/src/images/gif/brick_go.gif]

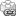

Supplement: Data S1. Source Code [file mmc2.zip › sourcecode/epitools_part1_matlab_v2.1.6/src/images/gif/brick_link.gif]

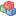

Supplement: Data S1. Source Code [file mmc2.zip › sourcecode/epitools_part1_matlab_v2.1.6/src/images/gif/bricks.gif]

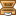

Supplement: Data S1. Source Code [file mmc2.zip › sourcecode/epitools_part1_matlab_v2.1.6/src/images/gif/briefcase.gif]

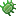

Supplement: Data S1. Source Code [file mmc2.zip › sourcecode/epitools_part1_matlab_v2.1.6/src/images/gif/bug.gif]

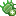

Supplement: Data S1. Source Code [file mmc2.zip › sourcecode/epitools_part1_matlab_v2.1.6/src/images/gif/bug_add.gif]

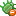

Supplement: Data S1. Source Code [file mmc2.zip › sourcecode/epitools_part1_matlab_v2.1.6/src/images/gif/bug_delete.gif]

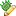

Supplement: Data S1. Source Code [file mmc2.zip › sourcecode/epitools_part1_matlab_v2.1.6/src/images/gif/bug_edit.gif]

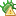

Supplement: Data S1. Source Code [file mmc2.zip › sourcecode/epitools_part1_matlab_v2.1.6/src/images/gif/bug_error.gif]

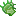

Supplement: Data S1. Source Code [file mmc2.zip › sourcecode/epitools_part1_matlab_v2.1.6/src/images/gif/bug_go.gif]

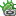

Supplement: Data S1. Source Code [file mmc2.zip › sourcecode/epitools_part1_matlab_v2.1.6/src/images/gif/bug_link.gif]

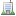

Supplement: Data S1. Source Code [file mmc2.zip › sourcecode/epitools_part1_matlab_v2.1.6/src/images/gif/building.gif]

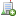

Supplement: Data S1. Source Code [file mmc2.zip › sourcecode/epitools_part1_matlab_v2.1.6/src/images/gif/building_add.gif]

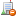

Supplement: Data S1. Source Code [file mmc2.zip › sourcecode/epitools_part1_matlab_v2.1.6/src/images/gif/building_delete.gif]

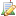

Supplement: Data S1. Source Code [file mmc2.zip › sourcecode/epitools_part1_matlab_v2.1.6/src/images/gif/building_edit.gif]

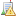

Supplement: Data S1. Source Code [file mmc2.zip › sourcecode/epitools_part1_matlab_v2.1.6/src/images/gif/building_error.gif]

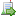

Supplement: Data S1. Source Code [file mmc2.zip › sourcecode/epitools_part1_matlab_v2.1.6/src/images/gif/building_go.gif]

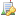

Supplement: Data S1. Source Code [file mmc2.zip › sourcecode/epitools_part1_matlab_v2.1.6/src/images/gif/building_key.gif]

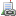

Supplement: Data S1. Source Code [file mmc2.zip › sourcecode/epitools_part1_matlab_v2.1.6/src/images/gif/building_link.gif]

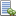

Supplement: Data S1. Source Code [file mmc2.zip › sourcecode/epitools_part1_matlab_v2.1.6/src/images/gif/buildings.gif]

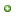

Supplement: Data S1. Source Code [file mmc2.zip › sourcecode/epitools_part1_matlab_v2.1.6/src/images/gif/bullet_add.gif]

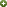

Supplement: Data S1. Source Code [file mmc2.zip › sourcecode/epitools_part1_matlab_v2.1.6/src/images/gif/bullet_add_1.gif]

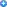

Supplement: Data S1. Source Code [file mmc2.zip › sourcecode/epitools_part1_matlab_v2.1.6/src/images/gif/bullet_add_2.gif]

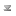

Supplement: Data S1. Source Code [file mmc2.zip › sourcecode/epitools_part1_matlab_v2.1.6/src/images/gif/bullet_arrow_bottom.gif]

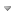

Supplement: Data S1. Source Code [file mmc2.zip › sourcecode/epitools_part1_matlab_v2.1.6/src/images/gif/bullet_arrow_down.gif]

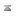

Supplement: Data S1. Source Code [file mmc2.zip › sourcecode/epitools_part1_matlab_v2.1.6/src/images/gif/bullet_arrow_top.gif]

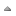

Supplement: Data S1. Source Code [file mmc2.zip › sourcecode/epitools_part1_matlab_v2.1.6/src/images/gif/bullet_arrow_up.gif]

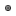

Supplement: Data S1. Source Code [file mmc2.zip › sourcecode/epitools_part1_matlab_v2.1.6/src/images/gif/bullet_black.gif]

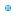

Supplement: Data S1. Source Code [file mmc2.zip › sourcecode/epitools_part1_matlab_v2.1.6/src/images/gif/bullet_blue.gif]

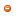

Supplement: Data S1. Source Code [file mmc2.zip › sourcecode/epitools_part1_matlab_v2.1.6/src/images/gif/bullet_delete.gif]

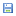

Supplement: Data S1. Source Code [file mmc2.zip › sourcecode/epitools_part1_matlab_v2.1.6/src/images/gif/bullet_disk.gif]

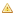

Supplement: Data S1. Source Code [file mmc2.zip › sourcecode/epitools_part1_matlab_v2.1.6/src/images/gif/bullet_error.gif]

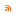

Supplement: Data S1. Source Code [file mmc2.zip › sourcecode/epitools_part1_matlab_v2.1.6/src/images/gif/bullet_feed.gif]

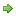

Supplement: Data S1. Source Code [file mmc2.zip › sourcecode/epitools_part1_matlab_v2.1.6/src/images/gif/bullet_go.gif]

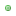

Supplement: Data S1. Source Code [file mmc2.zip › sourcecode/epitools_part1_matlab_v2.1.6/src/images/gif/bullet_green.gif]

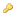

Supplement: Data S1. Source Code [file mmc2.zip › sourcecode/epitools_part1_matlab_v2.1.6/src/images/gif/bullet_key.gif]

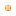

Supplement: Data S1. Source Code [file mmc2.zip › sourcecode/epitools_part1_matlab_v2.1.6/src/images/gif/bullet_orange.gif]

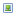

Supplement: Data S1. Source Code [file mmc2.zip › sourcecode/epitools_part1_matlab_v2.1.6/src/images/gif/bullet_picture.gif]

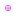

Supplement: Data S1. Source Code [file mmc2.zip › sourcecode/epitools_part1_matlab_v2.1.6/src/images/gif/bullet_pink.gif]

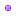

Supplement: Data S1. Source Code [file mmc2.zip › sourcecode/epitools_part1_matlab_v2.1.6/src/images/gif/bullet_purple.gif]

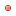

Supplement: Data S1. Source Code [file mmc2.zip › sourcecode/epitools_part1_matlab_v2.1.6/src/images/gif/bullet_red.gif]

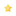

Supplement: Data S1. Source Code [file mmc2.zip › sourcecode/epitools_part1_matlab_v2.1.6/src/images/gif/bullet_star.gif]

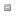

Supplement: Data S1. Source Code [file mmc2.zip › sourcecode/epitools_part1_matlab_v2.1.6/src/images/gif/bullet_toggle_minus.gif]

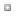

Supplement: Data S1. Source Code [file mmc2.zip › sourcecode/epitools_part1_matlab_v2.1.6/src/images/gif/bullet_toggle_plus.gif]

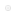

Supplement: Data S1. Source Code [file mmc2.zip › sourcecode/epitools_part1_matlab_v2.1.6/src/images/gif/bullet_white.gif]

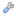

Supplement: Data S1. Source Code [file mmc2.zip › sourcecode/epitools_part1_matlab_v2.1.6/src/images/gif/bullet_wrench.gif]

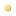

Supplement: Data S1. Source Code [file mmc2.zip › sourcecode/epitools_part1_matlab_v2.1.6/src/images/gif/bullet_yellow.gif]

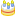

Supplement: Data S1. Source Code [file mmc2.zip › sourcecode/epitools_part1_matlab_v2.1.6/src/images/gif/cake.gif]

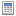

Supplement: Data S1. Source Code [file mmc2.zip › sourcecode/epitools_part1_matlab_v2.1.6/src/images/gif/calculator.gif]

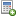

Supplement: Data S1. Source Code [file mmc2.zip › sourcecode/epitools_part1_matlab_v2.1.6/src/images/gif/calculator_add.gif]

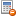

Supplement: Data S1. Source Code [file mmc2.zip › sourcecode/epitools_part1_matlab_v2.1.6/src/images/gif/calculator_delete.gif]

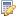

Supplement: Data S1. Source Code [file mmc2.zip › sourcecode/epitools_part1_matlab_v2.1.6/src/images/gif/calculator_edit.gif]

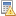

Supplement: Data S1. Source Code [file mmc2.zip › sourcecode/epitools_part1_matlab_v2.1.6/src/images/gif/calculator_error.gif]

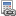

Supplement: Data S1. Source Code [file mmc2.zip › sourcecode/epitools_part1_matlab_v2.1.6/src/images/gif/calculator_link.gif]

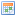

Supplement: Data S1. Source Code [file mmc2.zip › sourcecode/epitools_part1_matlab_v2.1.6/src/images/gif/calendar.gif]

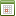

Supplement: Data S1. Source Code [file mmc2.zip › sourcecode/epitools_part1_matlab_v2.1.6/src/images/gif/calendar_1.gif]

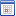

Supplement: Data S1. Source Code [file mmc2.zip › sourcecode/epitools_part1_matlab_v2.1.6/src/images/gif/calendar_2.gif]

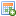

Supplement: Data S1. Source Code [file mmc2.zip › sourcecode/epitools_part1_matlab_v2.1.6/src/images/gif/calendar_add.gif]

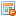

Supplement: Data S1. Source Code [file mmc2.zip › sourcecode/epitools_part1_matlab_v2.1.6/src/images/gif/calendar_delete.gif]

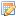

Supplement: Data S1. Source Code [file mmc2.zip › sourcecode/epitools_part1_matlab_v2.1.6/src/images/gif/calendar_edit.gif]

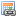

Supplement: Data S1. Source Code [file mmc2.zip › sourcecode/epitools_part1_matlab_v2.1.6/src/images/gif/calendar_link.gif]

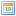

Supplement: Data S1. Source Code [file mmc2.zip › sourcecode/epitools_part1_matlab_v2.1.6/src/images/gif/calendar_view_day.gif]

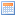

Supplement: Data S1. Source Code [file mmc2.zip › sourcecode/epitools_part1_matlab_v2.1.6/src/images/gif/calendar_view_month.gif]
