# Supplementary figures and images for: EpiTools: An Open-Source Image Analysis Toolkit for Quantifying Epithelial Growth Dynamics (part 1 of 2)
Source: Dev Cell. 2016 Jan 11;36(1):103–16. doi: 10.1016/j.devcel.2015.12.012 (PMC4712040; doi:10.1016/j.devcel.2015.12.012)

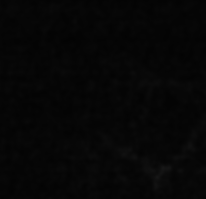

Supplement: Data S1. Source Code [file mmc2.zip › sourcecode/epitools_part1_matlab_v2.1.6/8_bit_sample/test_set.tif]

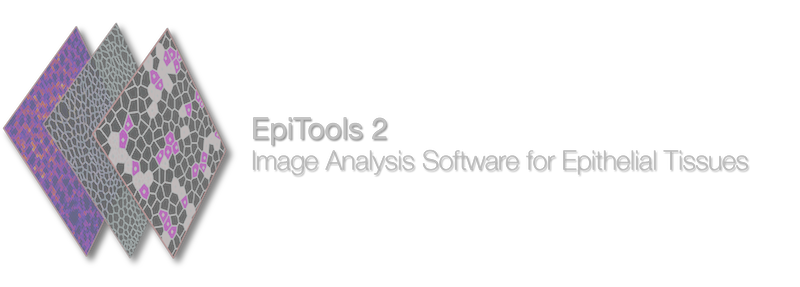

Supplement: Data S1. Source Code [file mmc2.zip › sourcecode/epitools_part1_matlab_v2.1.6/src/images/backgroundlogo.tif]

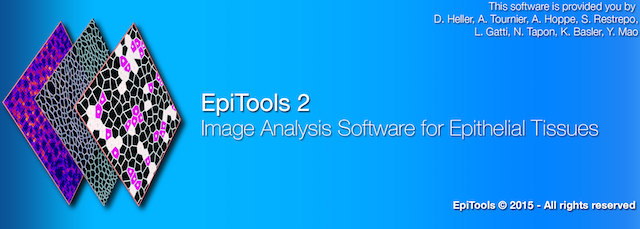

Supplement: Data S1. Source Code [file mmc2.zip › sourcecode/epitools_part1_matlab_v2.1.6/src/images/epitools_logo.png]

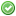

Supplement: Data S1. Source Code [file mmc2.zip › sourcecode/epitools_part1_matlab_v2.1.6/src/images/gif/accept.gif]

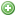

Supplement: Data S1. Source Code [file mmc2.zip › sourcecode/epitools_part1_matlab_v2.1.6/src/images/gif/add.gif]

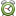

Supplement: Data S1. Source Code [file mmc2.zip › sourcecode/epitools_part1_matlab_v2.1.6/src/images/gif/alarm.gif]

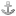

Supplement: Data S1. Source Code [file mmc2.zip › sourcecode/epitools_part1_matlab_v2.1.6/src/images/gif/anchor.gif]

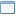

Supplement: Data S1. Source Code [file mmc2.zip › sourcecode/epitools_part1_matlab_v2.1.6/src/images/gif/application.gif]

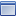

Supplement: Data S1. Source Code [file mmc2.zip › sourcecode/epitools_part1_matlab_v2.1.6/src/images/gif/application2.gif]

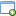

Supplement: Data S1. Source Code [file mmc2.zip › sourcecode/epitools_part1_matlab_v2.1.6/src/images/gif/application_add.gif]

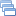

Supplement: Data S1. Source Code [file mmc2.zip › sourcecode/epitools_part1_matlab_v2.1.6/src/images/gif/application_cascade.gif]

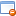

Supplement: Data S1. Source Code [file mmc2.zip › sourcecode/epitools_part1_matlab_v2.1.6/src/images/gif/application_delete.gif]

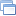

Supplement: Data S1. Source Code [file mmc2.zip › sourcecode/epitools_part1_matlab_v2.1.6/src/images/gif/application_double.gif]

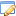

Supplement: Data S1. Source Code [file mmc2.zip › sourcecode/epitools_part1_matlab_v2.1.6/src/images/gif/application_edit.gif]

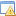

Supplement: Data S1. Source Code [file mmc2.zip › sourcecode/epitools_part1_matlab_v2.1.6/src/images/gif/application_error.gif]

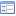

Supplement: Data S1. Source Code [file mmc2.zip › sourcecode/epitools_part1_matlab_v2.1.6/src/images/gif/application_form.gif]

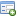

Supplement: Data S1. Source Code [file mmc2.zip › sourcecode/epitools_part1_matlab_v2.1.6/src/images/gif/application_form_add.gif]

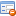

Supplement: Data S1. Source Code [file mmc2.zip › sourcecode/epitools_part1_matlab_v2.1.6/src/images/gif/application_form_delete.gif]

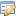

Supplement: Data S1. Source Code [file mmc2.zip › sourcecode/epitools_part1_matlab_v2.1.6/src/images/gif/application_form_edit.gif]

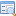

Supplement: Data S1. Source Code [file mmc2.zip › sourcecode/epitools_part1_matlab_v2.1.6/src/images/gif/application_form_magnify.gif]

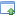

Supplement: Data S1. Source Code [file mmc2.zip › sourcecode/epitools_part1_matlab_v2.1.6/src/images/gif/application_get.gif]

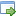

Supplement: Data S1. Source Code [file mmc2.zip › sourcecode/epitools_part1_matlab_v2.1.6/src/images/gif/application_go.gif]

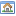

Supplement: Data S1. Source Code [file mmc2.zip › sourcecode/epitools_part1_matlab_v2.1.6/src/images/gif/application_home.gif]

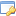

Supplement: Data S1. Source Code [file mmc2.zip › sourcecode/epitools_part1_matlab_v2.1.6/src/images/gif/application_key.gif]

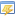

Supplement: Data S1. Source Code [file mmc2.zip › sourcecode/epitools_part1_matlab_v2.1.6/src/images/gif/application_lightning.gif]

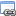

Supplement: Data S1. Source Code [file mmc2.zip › sourcecode/epitools_part1_matlab_v2.1.6/src/images/gif/application_link.gif]

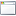

Supplement: Data S1. Source Code [file mmc2.zip › sourcecode/epitools_part1_matlab_v2.1.6/src/images/gif/application_osx.gif]

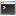

Supplement: Data S1. Source Code [file mmc2.zip › sourcecode/epitools_part1_matlab_v2.1.6/src/images/gif/application_osx_terminal.gif]

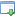

Supplement: Data S1. Source Code [file mmc2.zip › sourcecode/epitools_part1_matlab_v2.1.6/src/images/gif/application_put.gif]

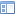

Supplement: Data S1. Source Code [file mmc2.zip › sourcecode/epitools_part1_matlab_v2.1.6/src/images/gif/application_side_boxes.gif]

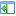

Supplement: Data S1. Source Code [file mmc2.zip › sourcecode/epitools_part1_matlab_v2.1.6/src/images/gif/application_side_contract.gif]

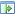

Supplement: Data S1. Source Code [file mmc2.zip › sourcecode/epitools_part1_matlab_v2.1.6/src/images/gif/application_side_expand.gif]

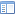

Supplement: Data S1. Source Code [file mmc2.zip › sourcecode/epitools_part1_matlab_v2.1.6/src/images/gif/application_side_list.gif]

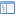

Supplement: Data S1. Source Code [file mmc2.zip › sourcecode/epitools_part1_matlab_v2.1.6/src/images/gif/application_side_tree.gif]

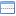

Supplement: Data S1. Source Code [file mmc2.zip › sourcecode/epitools_part1_matlab_v2.1.6/src/images/gif/application_split.gif]

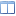

Supplement: Data S1. Source Code [file mmc2.zip › sourcecode/epitools_part1_matlab_v2.1.6/src/images/gif/application_tile_horizontal.gif]

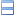

Supplement: Data S1. Source Code [file mmc2.zip › sourcecode/epitools_part1_matlab_v2.1.6/src/images/gif/application_tile_vertical.gif]

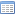

Supplement: Data S1. Source Code [file mmc2.zip › sourcecode/epitools_part1_matlab_v2.1.6/src/images/gif/application_view_columns.gif]

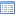

Supplement: Data S1. Source Code [file mmc2.zip › sourcecode/epitools_part1_matlab_v2.1.6/src/images/gif/application_view_detail.gif]

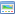

Supplement: Data S1. Source Code [file mmc2.zip › sourcecode/epitools_part1_matlab_v2.1.6/src/images/gif/application_view_gallery.gif]

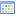

Supplement: Data S1. Source Code [file mmc2.zip › sourcecode/epitools_part1_matlab_v2.1.6/src/images/gif/application_view_icons.gif]

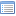

Supplement: Data S1. Source Code [file mmc2.zip › sourcecode/epitools_part1_matlab_v2.1.6/src/images/gif/application_view_list.gif]

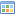

Supplement: Data S1. Source Code [file mmc2.zip › sourcecode/epitools_part1_matlab_v2.1.6/src/images/gif/application_view_tile.gif]

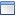

Supplement: Data S1. Source Code [file mmc2.zip › sourcecode/epitools_part1_matlab_v2.1.6/src/images/gif/application_view_xp.gif]

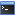

Supplement: Data S1. Source Code [file mmc2.zip › sourcecode/epitools_part1_matlab_v2.1.6/src/images/gif/application_view_xp_terminal.gif]

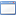

Supplement: Data S1. Source Code [file mmc2.zip › sourcecode/epitools_part1_matlab_v2.1.6/src/images/gif/application_xp.gif]

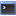

Supplement: Data S1. Source Code [file mmc2.zip › sourcecode/epitools_part1_matlab_v2.1.6/src/images/gif/application_xp_terminal.gif]

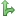

Supplement: Data S1. Source Code [file mmc2.zip › sourcecode/epitools_part1_matlab_v2.1.6/src/images/gif/arrow_branch.gif]

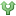

Supplement: Data S1. Source Code [file mmc2.zip › sourcecode/epitools_part1_matlab_v2.1.6/src/images/gif/arrow_divide.gif]

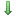

Supplement: Data S1. Source Code [file mmc2.zip › sourcecode/epitools_part1_matlab_v2.1.6/src/images/gif/arrow_down.gif]

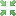

Supplement: Data S1. Source Code [file mmc2.zip › sourcecode/epitools_part1_matlab_v2.1.6/src/images/gif/arrow_in.gif]

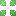

Supplement: Data S1. Source Code [file mmc2.zip › sourcecode/epitools_part1_matlab_v2.1.6/src/images/gif/arrow_inout.gif]

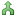

Supplement: Data S1. Source Code [file mmc2.zip › sourcecode/epitools_part1_matlab_v2.1.6/src/images/gif/arrow_join.gif]

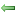

Supplement: Data S1. Source Code [file mmc2.zip › sourcecode/epitools_part1_matlab_v2.1.6/src/images/gif/arrow_left.gif]

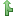

Supplement: Data S1. Source Code [file mmc2.zip › sourcecode/epitools_part1_matlab_v2.1.6/src/images/gif/arrow_merge.gif]

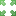

Supplement: Data S1. Source Code [file mmc2.zip › sourcecode/epitools_part1_matlab_v2.1.6/src/images/gif/arrow_out.gif]

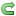

Supplement: Data S1. Source Code [file mmc2.zip › sourcecode/epitools_part1_matlab_v2.1.6/src/images/gif/arrow_redo.gif]

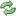

Supplement: Data S1. Source Code [file mmc2.zip › sourcecode/epitools_part1_matlab_v2.1.6/src/images/gif/arrow_refresh.gif]

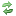

Supplement: Data S1. Source Code [file mmc2.zip › sourcecode/epitools_part1_matlab_v2.1.6/src/images/gif/arrow_refresh_small.gif]

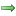

Supplement: Data S1. Source Code [file mmc2.zip › sourcecode/epitools_part1_matlab_v2.1.6/src/images/gif/arrow_right.gif]

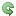

Supplement: Data S1. Source Code [file mmc2.zip › sourcecode/epitools_part1_matlab_v2.1.6/src/images/gif/arrow_rotate_anticlockwise.gif]

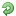

Supplement: Data S1. Source Code [file mmc2.zip › sourcecode/epitools_part1_matlab_v2.1.6/src/images/gif/arrow_rotate_clockwise.gif]

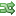

Supplement: Data S1. Source Code [file mmc2.zip › sourcecode/epitools_part1_matlab_v2.1.6/src/images/gif/arrow_switch.gif]

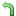

Supplement: Data S1. Source Code [file mmc2.zip › sourcecode/epitools_part1_matlab_v2.1.6/src/images/gif/arrow_turn_left.gif]

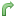

Supplement: Data S1. Source Code [file mmc2.zip › sourcecode/epitools_part1_matlab_v2.1.6/src/images/gif/arrow_turn_right.gif]

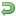

Supplement: Data S1. Source Code [file mmc2.zip › sourcecode/epitools_part1_matlab_v2.1.6/src/images/gif/arrow_undo.gif]

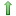

Supplement: Data S1. Source Code [file mmc2.zip › sourcecode/epitools_part1_matlab_v2.1.6/src/images/gif/arrow_up.gif]

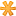

Supplement: Data S1. Source Code [file mmc2.zip › sourcecode/epitools_part1_matlab_v2.1.6/src/images/gif/asterisk_orange.gif]

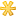

Supplement: Data S1. Source Code [file mmc2.zip › sourcecode/epitools_part1_matlab_v2.1.6/src/images/gif/asterisk_yellow.gif]

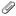

Supplement: Data S1. Source Code [file mmc2.zip › sourcecode/epitools_part1_matlab_v2.1.6/src/images/gif/attach.gif]

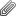

Supplement: Data S1. Source Code [file mmc2.zip › sourcecode/epitools_part1_matlab_v2.1.6/src/images/gif/attach_2.gif]

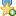

Supplement: Data S1. Source Code [file mmc2.zip › sourcecode/epitools_part1_matlab_v2.1.6/src/images/gif/award_star_add.gif]

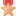

Supplement: Data S1. Source Code [file mmc2.zip › sourcecode/epitools_part1_matlab_v2.1.6/src/images/gif/award_star_bronze_1.gif]

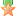

Supplement: Data S1. Source Code [file mmc2.zip › sourcecode/epitools_part1_matlab_v2.1.6/src/images/gif/award_star_bronze_2.gif]

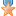

Supplement: Data S1. Source Code [file mmc2.zip › sourcecode/epitools_part1_matlab_v2.1.6/src/images/gif/award_star_bronze_3.gif]

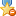

Supplement: Data S1. Source Code [file mmc2.zip › sourcecode/epitools_part1_matlab_v2.1.6/src/images/gif/award_star_delete.gif]

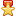

Supplement: Data S1. Source Code [file mmc2.zip › sourcecode/epitools_part1_matlab_v2.1.6/src/images/gif/award_star_gold.gif]

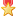

Supplement: Data S1. Source Code [file mmc2.zip › sourcecode/epitools_part1_matlab_v2.1.6/src/images/gif/award_star_gold_1.gif]

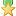

Supplement: Data S1. Source Code [file mmc2.zip › sourcecode/epitools_part1_matlab_v2.1.6/src/images/gif/award_star_gold_2.gif]

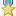

Supplement: Data S1. Source Code [file mmc2.zip › sourcecode/epitools_part1_matlab_v2.1.6/src/images/gif/award_star_gold_3.gif]

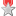

Supplement: Data S1. Source Code [file mmc2.zip › sourcecode/epitools_part1_matlab_v2.1.6/src/images/gif/award_star_silver_1.gif]

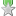

Supplement: Data S1. Source Code [file mmc2.zip › sourcecode/epitools_part1_matlab_v2.1.6/src/images/gif/award_star_silver_2.gif]

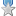

Supplement: Data S1. Source Code [file mmc2.zip › sourcecode/epitools_part1_matlab_v2.1.6/src/images/gif/award_star_silver_3.gif]

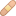

Supplement: Data S1. Source Code [file mmc2.zip › sourcecode/epitools_part1_matlab_v2.1.6/src/images/gif/bandaid.gif]

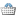

Supplement: Data S1. Source Code [file mmc2.zip › sourcecode/epitools_part1_matlab_v2.1.6/src/images/gif/basket.gif]

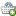

Supplement: Data S1. Source Code [file mmc2.zip › sourcecode/epitools_part1_matlab_v2.1.6/src/images/gif/basket_add.gif]

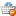

Supplement: Data S1. Source Code [file mmc2.zip › sourcecode/epitools_part1_matlab_v2.1.6/src/images/gif/basket_delete.gif]

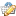

Supplement: Data S1. Source Code [file mmc2.zip › sourcecode/epitools_part1_matlab_v2.1.6/src/images/gif/basket_edit.gif]

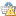

Supplement: Data S1. Source Code [file mmc2.zip › sourcecode/epitools_part1_matlab_v2.1.6/src/images/gif/basket_error.gif]

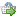

Supplement: Data S1. Source Code [file mmc2.zip › sourcecode/epitools_part1_matlab_v2.1.6/src/images/gif/basket_go.gif]

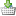

Supplement: Data S1. Source Code [file mmc2.zip › sourcecode/epitools_part1_matlab_v2.1.6/src/images/gif/basket_put.gif]

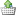

Supplement: Data S1. Source Code [file mmc2.zip › sourcecode/epitools_part1_matlab_v2.1.6/src/images/gif/basket_remove.gif]

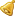

Supplement: Data S1. Source Code [file mmc2.zip › sourcecode/epitools_part1_matlab_v2.1.6/src/images/gif/bell.gif]

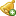

Supplement: Data S1. Source Code [file mmc2.zip › sourcecode/epitools_part1_matlab_v2.1.6/src/images/gif/bell_add.gif]

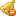

Supplement: Data S1. Source Code [file mmc2.zip › sourcecode/epitools_part1_matlab_v2.1.6/src/images/gif/bell_delete.gif]

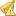

Supplement: Data S1. Source Code [file mmc2.zip › sourcecode/epitools_part1_matlab_v2.1.6/src/images/gif/bell_error.gif]

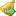

Supplement: Data S1. Source Code [file mmc2.zip › sourcecode/epitools_part1_matlab_v2.1.6/src/images/gif/bell_go.gif]

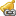

Supplement: Data S1. Source Code [file mmc2.zip › sourcecode/epitools_part1_matlab_v2.1.6/src/images/gif/bell_link.gif]

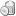

Supplement: Data S1. Source Code [file mmc2.zip › sourcecode/epitools_part1_matlab_v2.1.6/src/images/gif/bin.gif]

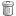

Supplement: Data S1. Source Code [file mmc2.zip › sourcecode/epitools_part1_matlab_v2.1.6/src/images/gif/bin_closed.gif]
