# Supplementary material for: EpiTools: An Open-Source Image Analysis Toolkit for Quantifying Epithelial Growth Dynamics
Source: Dev Cell. 2016 Jan 11;36(1):103–16. doi: 10.1016/j.devcel.2015.12.012 (PMC4712040; doi:10.1016/j.devcel.2015.12.012)
Supplement: Document S2. Article plus Supplemental Information [file mmc3.pdf]

Technology

Developmental Cell

EpiTools: An Open-Source Image Analysis Toolkit for Quantifying Epithelial Growth Dynamics

Graphical Abstract

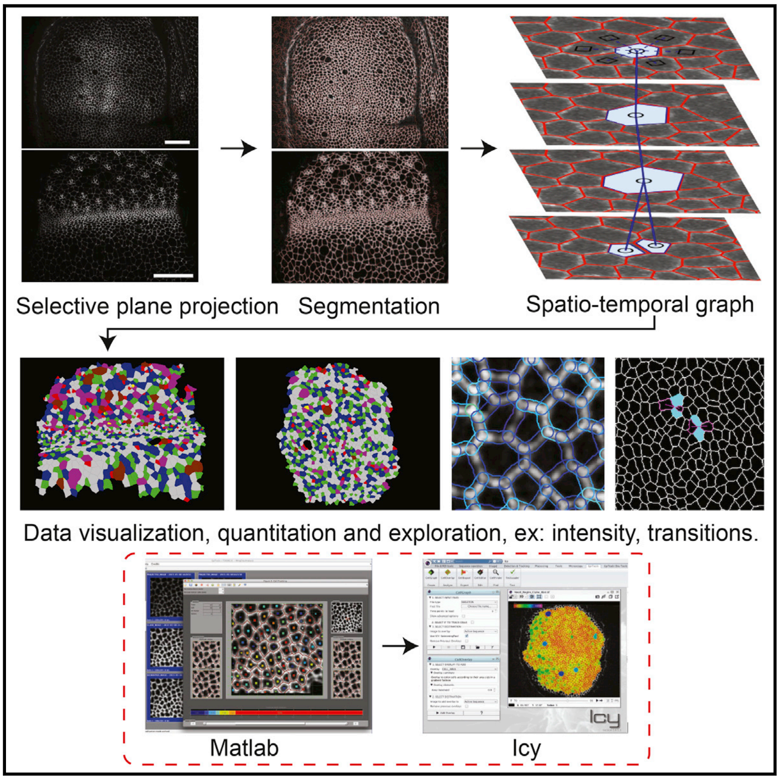

Authors

Davide Heller, Andreas Hoppe, Simon Restrepo, ..., Nicolas Tapon, Konrad Basler, Yanlan Mao

Correspondence

y.mao@ucl.ac.uk

In Brief

High-throughput analysis of time-lapse imaging data requires automated image quantification software. Heller et al. create a user-friendly image analysis toolkit, EpiTools, which segments, tracks, and quantifies cell contour data obtained from 4D imaging. They use this toolkit to obtain insight into cell dynamics in the developing *Drosophila* wing disc.

Highlights

- Increasing time-lapse data increases the demand for automated image quantification
- EpiTools, an open-source image analysis toolkit, addresses this need
- EpiTools segments, tracks, and quantifies cell contours from 4D imaging data
- EpiTools application provides insight into cell dynamics in the *Drosophila* wing disc

Heller et al., 2016, Developmental Cell *36*, 103–116
January 11, 2016 ©2016 The Authors
http://dx.doi.org/10.1016/j.devcel.2015.12.012

Developmental Cell
Technology

CellPress

EpiTools: An Open-Source Image Analysis Toolkit for Quantifying Epithelial Growth Dynamics

Davide Heller,[1,4,7] Andreas Hoppe,[2,7] Simon Restrepo,[1,7] Lorenzo Gatti,[1,3,4] Alexander L. Tournier,[5] Nicolas Tapon,[5] Konrad Basler,[1] and Yanlan Mao[6,*]
[1]Institute of Molecular Life Sciences, University of Zürich, Winterthurerstrasse 190, 8057 Zürich, Switzerland
[2]Digital Imaging Research Centre, Faculty of Science, Engineering and Computing, Kingston University, Kingston-upon-Thames KT1 2EE, UK
[3]Institute of Applied Simulations, Zürich University of Applied Sciences, Einsiedlerstrasse 31a, 8820 Wädenswil, Switzerland
[4]SIB Swiss Institute of Bioinformatics, Quartier Sorge - Batiment Genopode, 1015 Lausanne, Switzerland
[5]Apoptosis and Proliferation Control Laboratory, Lincoln's Inn Fields Laboratory, Francis Crick Institute, 44 Lincoln's Inn Fields, London WC2A 3LY, UK
[6]MRC Laboratory for Molecular Cell Biology, University College London, Gower Street, London WC1E 6BT, UK
[7]Co-first author
*Correspondence: y.mao@ucl.ac.uk
http://dx.doi.org/10.1016/j.devcel.2015.12.012
This is an open access article under the CC BY license (http://creativecommons.org/licenses/by/4.0/).

SUMMARY

Epithelia grow and undergo extensive rearrangements to achieve their final size and shape. Imaging the dynamics of tissue growth and morphogenesis is now possible with advances in time-lapse microscopy, but a true understanding of their complexities is limited by automated image analysis tools to extract quantitative data. To overcome such limitations, we have designed a new open-source image analysis toolkit called EpiTools. It provides user-friendly graphical user interfaces for accurately segmenting and tracking the contours of cell membrane signals obtained from 4D confocal imaging. It is designed for a broad audience, especially biologists with no computer-science background. Quantitative data extraction is integrated into a larger bioimaging platform, Icy, to increase the visibility and usability of our tools. We demonstrate the usefulness of EpiTools by analyzing *Drosophila* wing imaginal disc growth, revealing previously overlooked properties of this dynamic tissue, such as the patterns of cellular rearrangements.

INTRODUCTION

Multicellular tissues grow and develop in a complex and dynamic way. Final tissue size and architecture is determined by the coordination of cell divisions, cell death, cell shape changes, and cell rearrangements (Lecuit and Le Goff, 2007). Understanding the dynamic nature of how these processes are integrated in time and space is crucial to understanding tissue growth and morphogenesis. With recent advances in fluorescent light microscopy (Galland et al., 2015; Krzic et al., 2012), it has become increasingly possible to capture, at high temporal and cellular resolution, the dynamic processes of tissue growth and morphogenesis. This results in very large time-lapse datasets that are impossible to quantitatively analyze manually. The development of methods for automated image analysis, including cell segmentation and cell tracking, as well as analytical tools to quantitatively describe dynamic cellular behavior, is therefore the key to harnessing the power of in vivo imaging.

We have created EpiTools, a new image analysis toolkit for epithelial tissues. EpiTools is currently optimized for two-dimensional (2D) accurate cell contour segmenting and tracking of membrane labeled cells in epithelia, acquired originally as 4D (x, y, z, time) datasets using confocal microscopy. Aimed at a broad user audience, particularly biologists with little computer-science training, EpiTools has been designed to be easy to install and use, providing a guided analysis environment, yet being modular and extendable. We have ensured that the interfaces between the image segmentation and feature extraction modules are based on a simple standard format such that existing solutions, if preferred, can be readily used. The project Web site (http://tiny.uzh.ch/dm) delivers extensive support material and gives direct access to the software repositories, incentivizing modifications and extensions.

We have primarily used the *Drosophila* wing imaginal disc as an example tissue to demonstrate the functions of EpiTools. The *Drosophila* wing disc epithelium has been widely used as a model system to study the molecular and mechanical mechanisms of epithelial tissue growth (Aegerter-Wilmsen et al., 2012; Legoff et al., 2013; Mao et al., 2011, 2013; Shraiman, 2005). Until recent developments in ex vivo culturing of wing discs (Aldaz et al., 2010; Handke et al., 2014; Zartman et al., 2013), these studies had been limited to fixed tissue samples, masking the dynamic nature of the developmental process. Using EpiTools, we have now been able to fully exploit the power of the ex vivo culture and live imaging, to reveal new properties of this dynamically growing tissue which were previously overlooked. We have revealed new insights into how cell areas and cell shape (polygon) distributions change in different populations of cells as the epithelium develops, and how cell division orientations are regulated by cell shape. We have also

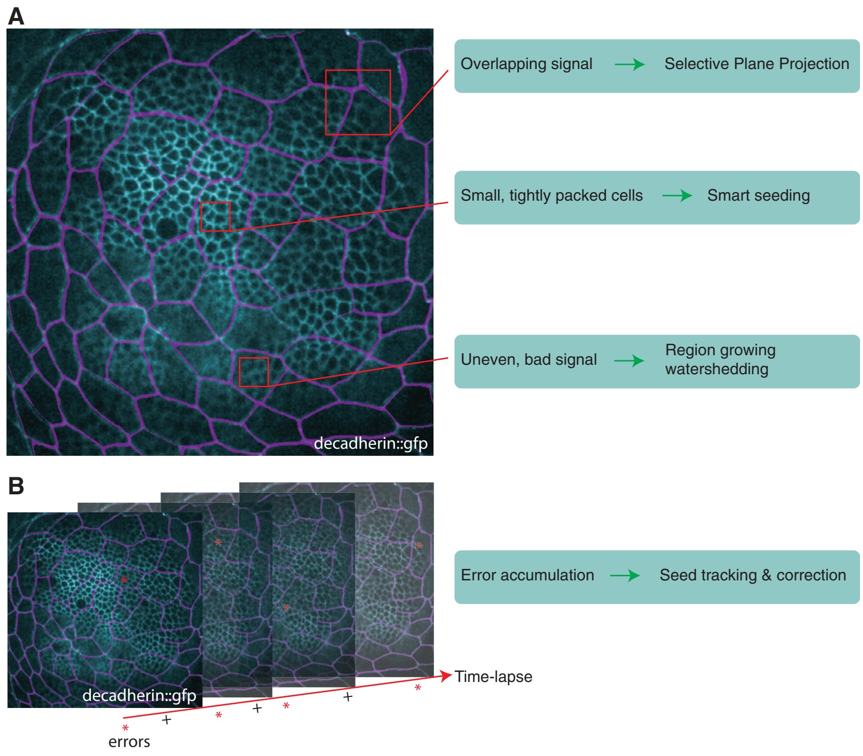

Figure 1. Image Segmentation Challenges
(A) E-cadherin::GFP-expressing wing disc. A maximum-intensity projection of a 3D wing disc image taken from a 4D time lapse. The peripodial membrane cells (magenta) are directly above the disc proper cells (cyan). The two signals must be untangled for proper segmentation of disc proper cell shapes. This requires a selective projection approach. A second challenge is posed by the tightly packed nature of the wing disc proper epithelium. The small size of the cells makes it difficult to resolve individual membranes. For this reason, the seeding of the watershedding algorithm must be optimal. Finally, the E-cadherin::GFP signal varies largely through the wing disc, which complicates the watershedding approach. Here, a region-growing watershedding algorithm better suited to this task was developed.
(B) The multiplication of time points in data series results in accumulation of errors (symbolized by asterisks). For this reason, the original segmentation must be as accurate as possible, but error correction steps must also be implemented.

systematically analyzed the spatial and temporal patterns of cell neighborhood relationships in the wing disc, and revealed patterns of cell intercalations and fluid-like junctional dynamics in a tissue previously thought to lack cell rearrangements (Bryant, 1970; Garcia-Bellido et al., 1973; Gibson et al., 2006; Resino et al., 2002).

DESIGN

Although several cell segmentation and tracking software suites have been developed (Table S1), including Packing Analyser (Aigouy et al., 2010), MorphographX (Barbier de Reuille et al., 2015; Kierzkowski et al., 2012), EDGE (Gelbart et al., 2012), Edge4D (Khan et al., 2014), (Blanchard et al., 2009), SeedWaterSegmenter (Mashburn et al., 2012), ilastik (Sommer et al., 2011), and TTT (Cilla et al., 2015), their adoption by the extended research community has often been slow. In particular, accessibility to biologists with limited computational experience has been a limiting factor. Moreover, due to the morphological diversity of biological systems, and therefore of acquired images, establishing a complete analysis pipeline for 3D time lapses presents many challenges. Several software packages often need to be combined and further extended by custom-written routines, which have to be adapted for each new biological question. The lack of user-friendly interfaces requires programming skills in various languages and handling of non-standardized file formats. Finally, connectivity to larger bioimaging platforms such as ImageJ or Icy, with which the user may already be familiar, is generally missing. Designed to overcome these limitations, EpiTools consists of a user-friendly image analysis framework with a graphical user interface (GUI) in MATLAB for processing of the raw images as well as a collection of software extension modules (plugins) for feature extraction, analyses, and visualization in Icy (de Chaumont et al., 2012). This modularity allows for processes to be replaced or extended with third-party techniques and tools.

RESULTS

EpiTools Part 1a: An Image Segmentation Method for Epithelial Time-Lapse Data

Since many epithelial tissues consist of a cell monolayer, with cells growing, dividing, and moving in the plane of the tissue, a 2D planar projection of cell shapes is often a good approximation for understanding the dynamic behavior of the tissue. However, most epithelia are not flat sheets of cells, but can be considerably curved (Escudero et al., 2007; Osterfield et al., 2013; Sweeton et al., 1991), and may closely appose other cells or features outside of the plane of interest, which are inevitably captured during the imaging process. An example of such a challenging tissue is the *Drosophila* wing disc, which consists of two cellular layers on a dome-shaped surface: a dense mesh of columnar wing disc proper cells and a looser mesh of squamous peripodial cells on a different focal plane (Figures 1 and 2A). Large fluctuations in signal intensities, poor signal-to-noise ratios (to minimize tissue damage from long-term time-lapse imaging), and cells of varying sizes within the image volume make this a challenging segmentation problem. It is, however, critical to identify individual cells within the mesh so that the spatial and temporal relationships between neighboring cells can be quantified from the precise geometry of the membranes.

The following methods outline the image-processing procedures that EpiTools uses to achieve this task. We designed a selective plane projection that follows the curvature of the tissue in order to overcome the limitations of a simple maximum-intensity

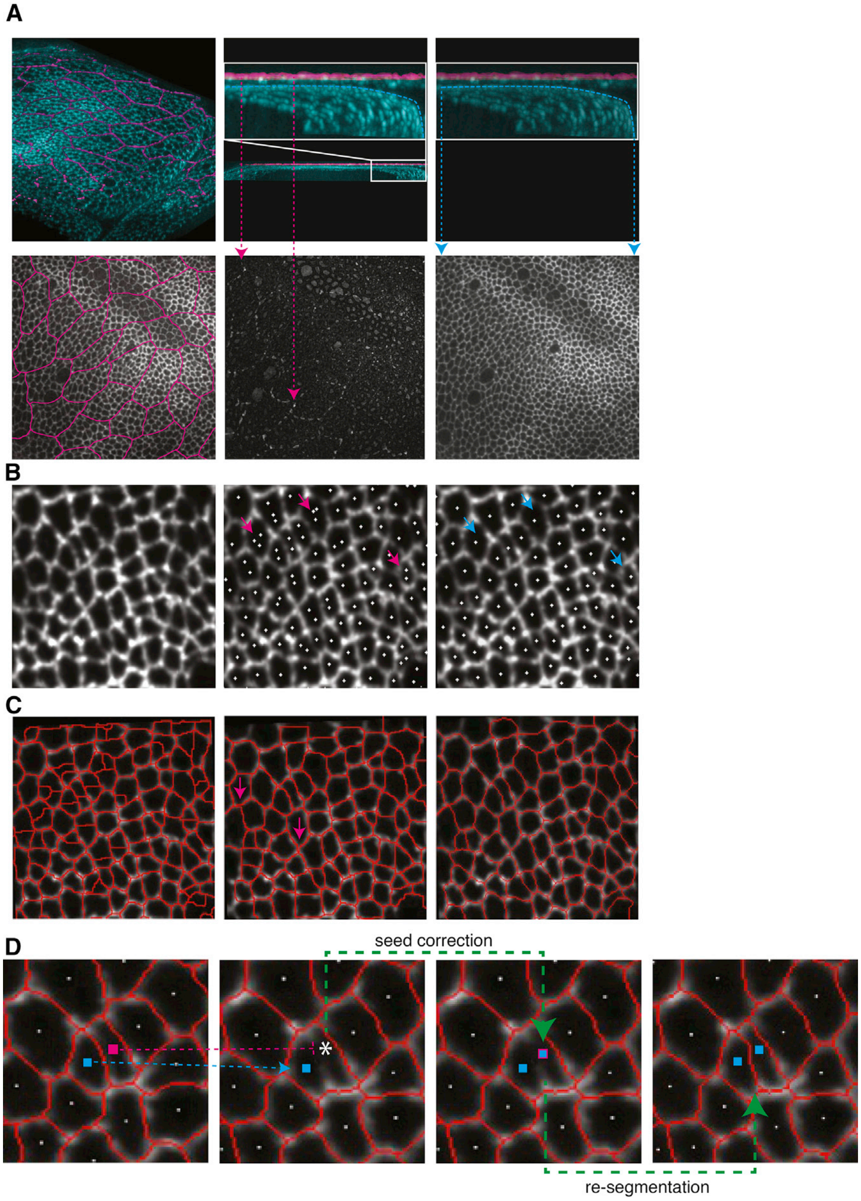

Figure 2. Image Segmentation Method

(A) The *Drosophila* wing disc is a dome-shaped epithelial tissue consisting of two cellular layers: a dense mesh of disc proper cells (cyan) and a less dense mesh of peripodial membrane cells (magenta). Top center and right: a section through the image volume with the highlighted disc proper layer (cyan) and peripodial layer (magenta). The peripodial membrane needs to be separated from the wing disc, as these two layers can interfere with each other (bottom left: signal from both layers when maximally projected; bottom center: signal from just the peripodial layer). The selective plane projection accurately fits a surface to the disc proper layer (cyan line), taking into account its shape while excluding the peripodial layer. The result is a clean projected image (bottom right).

(B) Automatic seed generation. Cut-out region from the selective plane projected image is shown after Gaussian smoothing has been applied (left). Wing disc projection with seeds generated from MATLAB watershed regions (center). Magenta arrows highlight some of the areas with multiple seeds per cell. Wing disc with seeds generated using our seed point generation algorithm (right). The number of duplicated seeds is greatly reduced, as highlighted by the cyan arrows.

(C) Segmentation result of cellular regions overlaid on wing disc image. Cell boundaries generated from MATLAB watershed algorithm (left). Cell boundaries generated from MATLAB watershed algorithm with manually homogenized cell regions (middle). Magenta arrows highlight inaccuracies with the cell boundary segmentation. Our region-growing algorithm, using the same seed points as in the center image, generates more accurate cell boundaries (right).

(D) Seed tracking allows identification of broken tracks due to segmentation errors, which are easily rectified and subsequently re-segmented. This process is depicted in the first three images while the last image shows the corrected segmentation. Cyan highlights correct seeds. The first two images highlight in magenta a broken track between two frames. The third image shows how the missing seed point can be added manually in the second image with a single mouse click. Re-segmentation then provides the correct boundary segmentation result.

projection (Figure 2A). It consists of a two-step surface-fitting procedure and requires that voxels with high intensity mostly belong to the desired layer (e.g. the disc proper signal in the wing disc). We use the notion of stiffness to describe the flexibility of the fitting and interpolating method (D'Errico, 2006) to accommodate outliers lying far apart from estimated surface location (e.g. signal from peripodial membrane in the wing disc). In the first step of the method we choose an increased stiffness such that the fitted surface settles coarsely on the desired layer, ignoring points that lie far apart. This surface is used to exclude from the high-intensity signal points which have, with respect to the surface, a higher than threshold distance (e.g. peripodial membrane signal). In the second step a less stiff fitting is performed on the refined signal to follow the curvature of the desired layer accurately (Figure 2A, cyan line). Finally, pixel intensities along the fitted surface are used to form the projected image. The second 3D surface fit can be exported and used to correct subsequent geometric analyses, if necessary (Figure S1).

The projected images are aligned (registered) to compensate possible sample movement during the acquisition. The image registration step can also be performed through external software such as the StackReg plugin for ImageJ (Thevenaz et al., 1998) controlled via EpiTools using the MIJ interface (Sage, 2012).

On the registered images we apply region-growing segmentation. The aim of this step is to ideally first create a single seed point per cell from which to grow cellular regions (Figure 2B). Seed points conceptually represent cell centers and can be corrected with a simple mouse click to add, remove, or fuse cellular regions. Our seed point generation method was devised to include growing and merging of regions to reduce fragmentation:

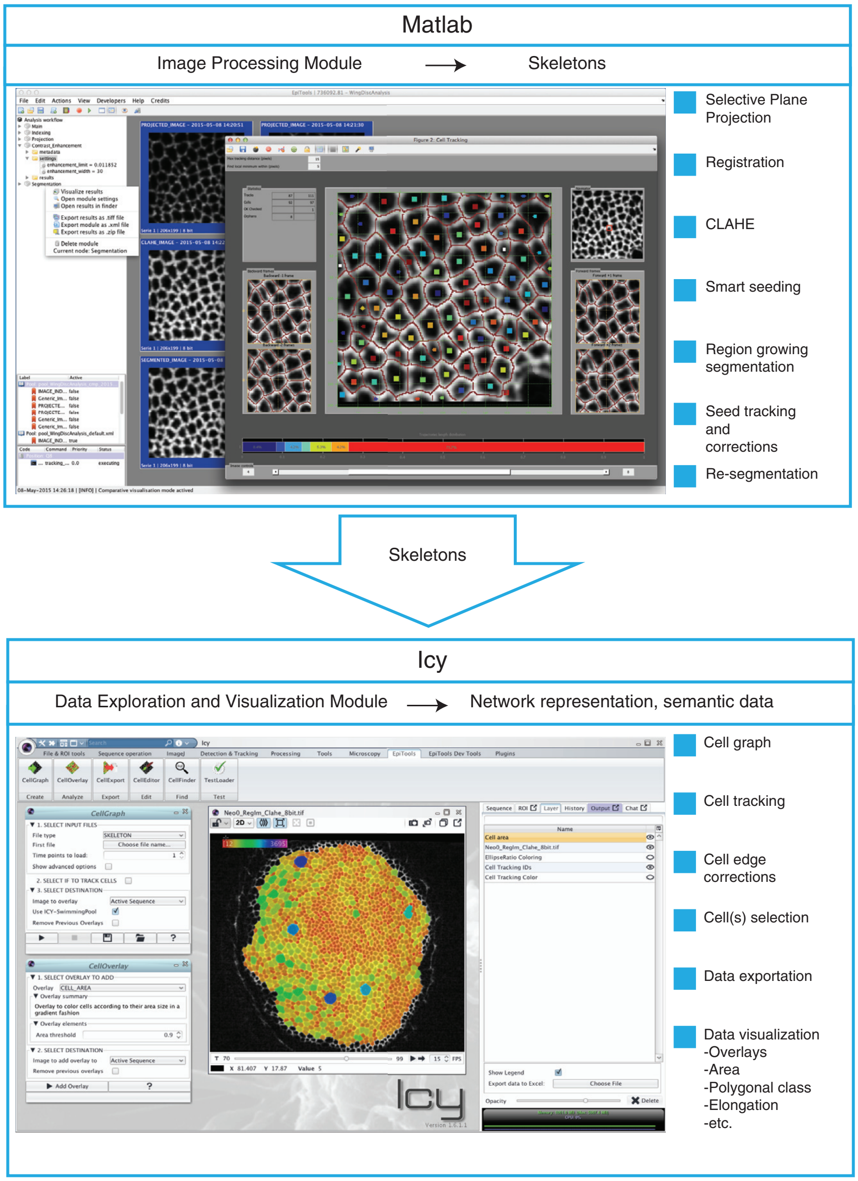

Figure 3. EpiTools Overview

EpiTools consists of two complementary parts. First, an image-processing pipeline (Part 1, MATLAB) allows the user to preprocess the image prior to segmentation and to segment the image. The output of Part 1 consists of skeletons of the cell membranes. Part 2 uses Icy to provide a user-friendly interface for data visualization and analysis. An image-understanding module (CellGraph) enables the user to produce a cell graph, which gives a semantic description of the tissue of interest. The data exploration and visualization can be directly visualized as overlays in Icy (CellOverlay) or be exported as Excel sheets (xls) or graphML (xml) files (CellExport).

homogeneous regions of a certain size below a rising signal intensity threshold (cell boundary signal) are identified and allocated to become new cellular regions with a unique identification. Cellular regions are grown from these seed points by assimilating neighboring pixels below an increasing intensity threshold. The region growing is performed in parallel for each seed point and is guided by the local intensity flow, climbing up the intensity gradients that separate cells which create distinct boundaries between cells (Figure 2C). Finally, automatic temporal seed tracking has been implemented to identify seeding errors expressed as a discontinuity in temporal cell tracks, which leads to an efficient error correction (Figure 2D). For example, a missing seed point is identified by a broken track (Figure 2D, magenta track) often due to segmentation errors. The error can be easily rectified by adding a new seed (Figure 2D, seed correction) and re-segmentation can be applied, producing the final segmented frame (Figure 2D, re-segmentation). The final series of segmented frames can be exported as skeletons which accurately represent cell junction (or membrane) signals, as opposed to using linear approximations (Cilla et al., 2015). This is important because an accurate representation of the curvature of cell junctions is critical for understanding the mechanical properties of the cells (Brodland et al., 2014).

A detailed description of these processing steps can be found in Supplemental Experimental Procedures. All image-processing and analysis techniques were implemented in MATLAB (Mathworks). The region-growing and seed-tracking technique was implemented as compiled C extensions for MATLAB to reduce processing time.

EpiTools Part 1b: Framework and User Interface, EpiTools-MATLAB GUI

To optimize the ease of use, we split EpiTools into two parts (Figure 3). Part 1 is primarily a MATLAB (and C)-based analysis framework, with a bespoke GUI; Part 2 consists of EpiTools modules (plugins) for an existing image analysis platform, Icy (see below). Part 1 processes the images through the modular steps described above to eventually produce skeletons of the images that can be exported for further analysis in Part 2 (Figures 3 and S1). With the introduction of an EpiTools-MATLAB GUI, we wanted to expand the panorama of possibilities our end users have to complete their image analysis. The idea behind the current implementation was to separate the analysis workflow into single independent steps that can be called repeatedly for best parameterization as well as skipped if not needed. Moreover, we wanted this to be as easy and intuitive as possible, especially for users with little computer-science background.

The EpiTools-MATLAB GUI presents a series of menus where the user finds all the main components needed to run the analysis (see our video tutorials, which provide detailed step-by-step guides: http://tiny.uzh.ch/dN). Menus are divided according to function scope, and additional submenus guide the user to

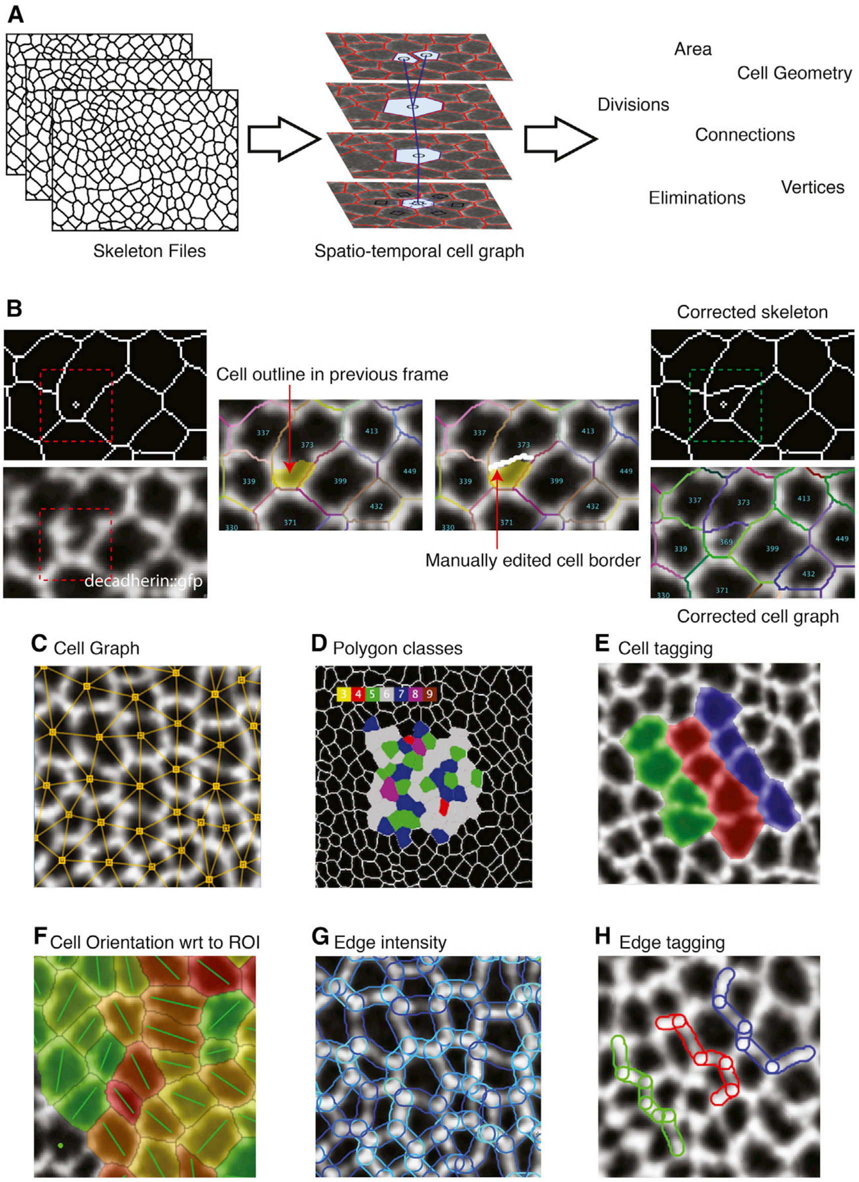

Figure 4. EpiTools Icy

(A) The visualization and analysis module of EpiTools produces a cell graph based on the skeletons. The data structure provides a semantic understanding of the tissue and easy access to important values and events such as cell area, cell geometry, and cell divisions.

(B) The cell editor plugin allows the user to employ abnormal changes in cell topologies to zoom into putative segmentation mistakes. If mistakes are identified, the user can use data from the unsegmented images to manually correct improperly segmented or missing cell borders.

(C–H) The visualization and analysis module of EpiTools contains plugins that allow the user to generate and visualize data of interest. We use the overlay feature of Icy to superimpose the desired information onto skeletons or the original imaging time-lapse data. ROI, region of interest; wrt, with respect to. For a full list of overlays and explanations for each, please visit http://tiny.uzh.ch/dT.

customize the analysis environment. We have also implemented context menus (right-click), which allow exporting and visualizing the connected files. The user can easily invoke them from the analysis workflow tree on the left side of the main window, clicking on the corresponding analysis module.

Each analysis module has a sub-window that collects all the procedures, inputs, and parameters required to execute it. We provide detailed explanations of every parameter (http://tiny.uzh.ch/dS) and recommended values to initially try. We designed a special independent GUI for the Seed Tracking module, highlighting the seeds that need corrections and offering various operations to assist the user in the manual corrections.

Parameter choices affect the output of many modules and can have lasting consequences on subsequent analysis steps. Therefore, we included a set of built-in visualization tools and comparison modes to help the parameterization. The comparative mode allows for easy comparison of different parameters to find the optimal parameter set for a given task. In addition, EpiTools offers connectivity to Icy, such that the user can make a more detailed analysis regarding the difference between the module executions (see Icy's Sequence comparator: http://icy.bioimageanalysis.org/plugin/Sequence_comparator).

The EpiTools Part 1 analysis files are created to achieve reproducible image analysis. User inputs, outputs, parameter sets, and all the associated metadata for each analysis step are stored in a clear xml file, which can be easily accessed from third-party applications.

EpiTools Part 2: Network Analysis and Data Structure, EpiTools Icy

The skeleton images produced by EpiTools Part 1 represent a common intermediary step (Aigouy et al., 2010; Mashburn et al., 2012), as yet unsuitable for manual analysis. It is therefore necessary to create a computational description of how the individual frames relate to each other and automatically capture changes. To this end, we developed EpiTools Part 2, a package that transforms the skeleton files into a computational graphic data structure (Figures 3 and 4A). This network-like data structure contains the neighborhood relationships between cells in the tissue in the form of nodes and edges. We use the term spatiotemporal graph to refer to this particular type of graph because we include both spatial (within the same frame) and temporal (between different frames) neighborhoods. Similar approaches can be found in the literature (Gelbart et al., 2012; Gunduz et al., 2004; Liu et al., 2010), and have been taken as inspiration for this approach.

We chose the bioimaging platform Icy (de Chaumont et al., 2012) as the framework for our package to provide rich visual feedback to the user. This software delivers remarkable

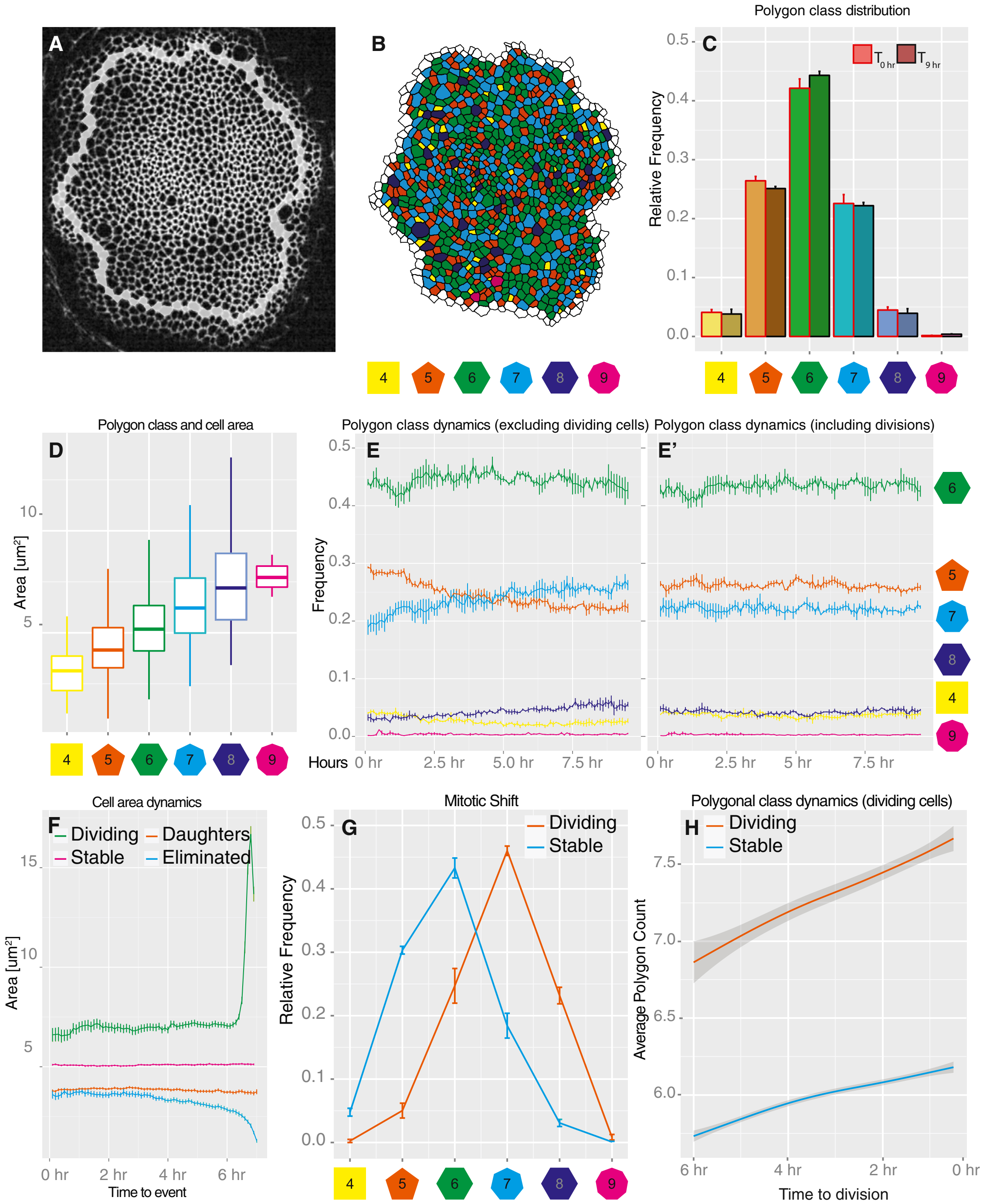

Figure 5. *Drosophila* Wing Disc: Polygon Distribution and Cell Area Dynamics

If not stated otherwise, error bars indicate SEM in all figures.

(A) Representative example of the wing pouch section of a wing disc (E-cadherin::GFP). Overlaid white cells represent the segmentation border.

(B) Same frame as (A), segmented and processed with EpiTools. The polygon class of each cell is indicated by a color code.

(C) The frequency of the polygon classes corresponding to cells with n number of neighbors remains constant over time.

(D) On average, the area of cells correlates well with their polygon class. However, there is a large degree of variance. Boxplot whiskers indicate 1.5× interquartile range (IQR), hinges IQR, and inner lines the median.

(E and E′) Among non-dividing cells the frequency of certain polygon classes increases over time (E). This effect is no longer visible when dividing cells (E′), and thus daughter cells are included in the analysis.

(F) Area comparison between cell classes over time. First, cells that can be observed for at least 1 hr (10 frames) are selected from three samples (3,086 cells) and classified according to four classes: daughters (observed offspring in the movie, n = 686), dividing (observed dividing in the movie, n = 303), eliminated (n = 88),

(legend continued on next page)

user-friendliness and offers many image analysis tools for biological samples. In addition, the Icy platform facilitates sharing, in the form of plugins (i.e. software modules that extend the original capabilities) and analysis protocols. The main project Web site serves as a central hub to inform about available plugins and to allow user exchange. For more information about Icy, we recommend visiting the project homepage (http://icy.bioimageanalysis.org).

The EpiTools package for Icy consists of multiple plugins that address the subsequent steps of the analysis: CellGraph (Figure 4A), which generates the spatiotemporal graph starting from input skeleton files; CellEditor (Figure 4B), which enables the user to interactively modify the skeleton images manually in case of any remaining segmentation mistakes; CellOverlay (Figures 4C–4H, and S2), which interprets the data and outputs results in the form of graphical overlays (i.e. additional image layers) and tabular files; and CellExporter, which allows the user to export the complete numerical data in various formats, such as Excel and GraphML. Every plugin has a separate GUI and can be conveniently accessed through the EpiTools toolbar (see video tutorials at http://tiny.uzh.ch/dO). To facilitate data query, we have developed many commonly required analysis features (in the form of overlays, Figures 4C–4H), including, for example, cell areas, cell elongation ratios, cell intercalations, edge intensities, and more interactive features, such as how cell orientation changes with respect to a defined point of interest that can be interactively changed by the user (Figure 4F). For a full list of the overlays available (Figure S2), please visit http://tiny.uzh.ch/dT.

The spatiotemporal graph structure created by the CellGraph plugin is built in three main steps. First, the cell geometries are extracted from the supplied skeletons. Second, the geometry objects are inserted into a graph representing the spatial neighborhood based on intersection. Finally, temporal linkage is added by matching the spatial graphs representing each frame. To achieve this, we employ graph-matching algorithms and apply heuristics to analyze the unmatched cells. The latter might correspond to divisions or eliminations, or suggest a segmentation mistake.

We emphasized the visual elaboration of our graph structure because we found that visual analysis is very helpful in formulating hypotheses before exporting the data for statistical analysis. The overlays created by the CellOverlay plugin use the layer feature of Icy's image viewer and adapt automatically to the position in space and time. For example, Figure 3 (bottom half) shows an overlay that highlights all cell geometries with a gradient color scheme according to the apical cell area. The user is thus given a natural interpretation of how the area sizes are distributed in the tissue.

To quantitatively analyze the data, the user can generate an Excel sheet from every overlay focusing on the visualized quantities, or access more general export options through the CellExport plugin. Among many, we highlight the XML-based graph format called GraphML (Brandes et al., 2002), which stores the neighborhood relationships of the cells. The format can be easily read by many scripting languages such as R or Python. An example analysis file can be downloaded from the project homepage (http://tiny.uzh.ch/dP).

The surface estimated by the selective plane projection (EpiTools Part 1a) can be rendered as 3D Mesh ROI (Figure S1B) with the CellSurface plugin (http://icy.bioimageanalysis.org/plugin/3D_Mesh_ROI). Moreover, cells can be colored according to their estimated surface normal with the Projection overlay (Figure S1B′). For detailed information and utility, we refer the reader to http://tiny.uzh.ch/s3.

We implemented the Icy plugins in Java using two main libraries: the Java topology suite (http://www.sourceforge.net/projects/jts-topo-suite/) to manage the geometries of cells and the jgraphT library (http://www.jgrapht.org) to store the graph structure. Icy's shared plugin memory (swimming pool) is used to allow communication across the plugins. Please see Supplemental Information for more details.

For specific help on how to install and use our plugins, please visit our project Web site where we provide tutorials for every component (http://tiny.uzh.ch/dQ). The source code is provided with open-source license at the public Git repository https://bitbucket.org/davideheller/epitools/ and is provided here as a zip file (Data S1).

Analysis of Different Epithelia using EpiTools

To test the versatility of EpiTools, we processed different epithelia in *Drosophila* with varying cell areas and cellular heterogeneities (Figure S3). The *Drosophila* wing imaginal disc (Figures 5A and S3A) was our main tissue of focus (see sections below), but EpiTools was also able to segment, with high precision, membrane signals from time-lapse images of *Drosophila* eye imaginal discs (Figure S3B), histoblast nests (Figure S3C), and embryos (Figure S3D). We show here mainly the results of single frames for ease of representation. Although there are still some segmentation errors visible in Figures S3B–S3D, these were deliberately obtained without any manual corrections, showing the high accuracy of the automated segmentation process, provided that correct parameters are used (see Experimental Procedures, Table 1, and our guides to parameters on our Web site http://tiny.uzh.ch/dS). In the eye imaginal disc, we were able to track the rearrangements of cells as they exit the morphogenetic furrow (Figure S4, 0 min) through their formation into arcs (60 min), to their eventual recruitment into ommatidial preclusters (240 min).

Drosophila Wing Disc Analysis I: Proof of Principle and Insights into Epithelial Geometry and Cell Division Dynamics

Epithelia assume cell-packing geometries characterized by different cell areas and neighbor-number distributions. Cells can be classified by their number of neighbors into sets of

and stable (remainder, n = 2,009). Second, to compare area sizes across classes we transform the temporal axis with respect to the class: daughter's origin time (frame after division) is aligned to 0 hr, dividing cell's ending frame (frame of division) is aligned to 7 hr and equally so for eliminated cells (frame before elimination). For stable cells we used 7 hr of continuous observation beginning from the start of the movie.

(G) EpiTools correctly detects the expected +1 shift in the frequency of polygon classes among dividing cells versus non-dividing cells.

(H) Both dividing cells and non-dividing cells accumulate neighbors over time. However, the increase is twice as fast for dividing cells. Envelope indicates 0.95 confidence level.

Table 1. Parameters Used for Segmenting Images

Web Site Name	XML Code	Wing	Eye	Histoblast	Embryo
Projection					
Smoothing Radius	SmoothingRadius	1	1	1	1
Surface Smoothness 1	SurfSmoothness1	30	30	30	30
Cutoff distance	ProjectionDepthThreshold	1.2	1.2	1.2	1.2
Surface Smoothness 2	SurfSmoothness2	20	20	20	20
CLAHE					
Enhancement limit	enhancement_limit	0.02	0.02	#	0.02
Enhancement width	enhancement_width	30	30	#	30
Segmentation					
Gaussian Blur Kernel	sigma1	2	1	1	1
Minimum cell area	mincellsize	25	15	25	25
Minimal membrane intensity	threshold	25	20	25	25
Boundary Low Intensity Ratio	MergeCriteria	0.15	0.45	0.35	0.35
Gaussian Blur Kernel	sigma3	0.5	2	2	2
Largest Cell Area	LargeCellSizeThres	3,000	4,300	3,000	3,000
Minimal mean intensity	IBoundMax	30	20	30	30

polygons. Interestingly this geometric order tends to remain apparently unperturbed by the changes introduced by cell divisions (Farhadifar et al., 2007; Gibson et al., 2006). We used EpiTools to study the geometric order of the *Drosophila* wing disc, dynamically, on growing discs. Of note, previous quantifications were mainly done on fixed samples, whereas in this study we examine live discs. In this way we can directly assay the interplay between epithelial dynamics and cell division. The quantitative data generated with EpiTools agrees well with previous reports and expectations, but also provides insights into the interplay between cell divisions and epithelial geometry.

First, we examined the frequency of n-sided cells in wing discs. We obtained polygon frequencies in good agreement with previous reports (Gibson et al., 2006; Figure 5C). A comparison of the polygon distribution 6 hr apart confirms that the frequencies remain constant (Figure 5C). Previous reports have indicated that in the wing disc, cell area correlates with polygon count, thus obeying Lewis's law (Farhadifar et al., 2007; Lewis, 1928). Our data confirm this, but show a large degree of variation (Figure 5D). Next, we looked at whether differences in cell geometry also correlated with different cell fates (such as dividing cells and dying cells). Since we are now able to track cells and have a semantic interpretation of the time lapse, we can select specific cell classes based on their behavior: dividing cells, new (daughter) cells, stable cells, and eliminated cells. The apical area of daughter cells is half that of dividing cells (Figure 5F). This indicates that, assuming cell height remains constant, wing disc cells double in volume prior to division. Our data also indicate that stable cells are larger than daughter cells (Figure 5F) and are likely a population of cells that is either in S or G1. Furthermore, we identified cells that are eliminated during the recordings (Figures 5F and S5). Interestingly, these cells can be identified as the smallest class of cells (Figure 5F). Further analyses of these cells revealed that they were eliminated by a process reminiscent of live cell delaminations. To better study this phenomenon, we employed the edge-tracking feature of EpiTools. By tracking the edges of the delaminated cells, we quantified the intensity of the E-cadherin signal over time and confirmed that E-cadherin signal intensity did not diminish prior to elimination (Figures S5A″ and S5D). The stability of E-cadherin is a hallmark of live cell delamination, where E-cadherin is not reduced on cell junctions, and can be used to differentiate this type of cell elimination from apoptosis, whereby E-cadherin is lost from junctions prior to cell elimination (Marinari et al., 2012). We then confirmed that delaminating cells seemed to, on average, lose edges prior to delamination (Figure S5E), a second characteristic of live cell delaminations (Marinari et al., 2012).

Next, we looked at how the polygon count of cell classes evolves over time. Interestingly, among the cells that were not observed to divide during our imaging window (Figure 5E) the frequency of n-sided neighbors does not remain constant. Specifically, the frequency of pentagons decreases while that of heptagons increases (Figure 5E). However, if one considers the population as a whole, this effect disappears (Figure 5E′). This supports the idea that the allocation of neighbors after cell division contributes to keeping the fraction of n-sided cells constant (Gibson et al., 2006).

We found that mitotic cells have on average one extra side compared with stable cells (Figure 5G), as expected (Gibson et al., 2006, 2014). This can be observed several hours prior to division, confirming that dividing cells have been accumulating neighbors over time (Figure 5H) (Gibson et al., 2014). All cells tend to have an increase in number of neighbors over time, but this effect is stronger for dividing cells than for stable cells (Figure 5H).

Another well-studied phenomenon that we analyzed with EpiTools is the link between cell geometry and cell division orientation. Consistent with previous studies in the wing disc (Gibson et al., 2011; Mao et al., 2011) we found that cells that are significantly elongated, with an elongation ratio (major/minor axis) greater than 1.3, tend to divide to bisect their long axis, i.e. the new junction is near perpendicular to the long axis of the cell

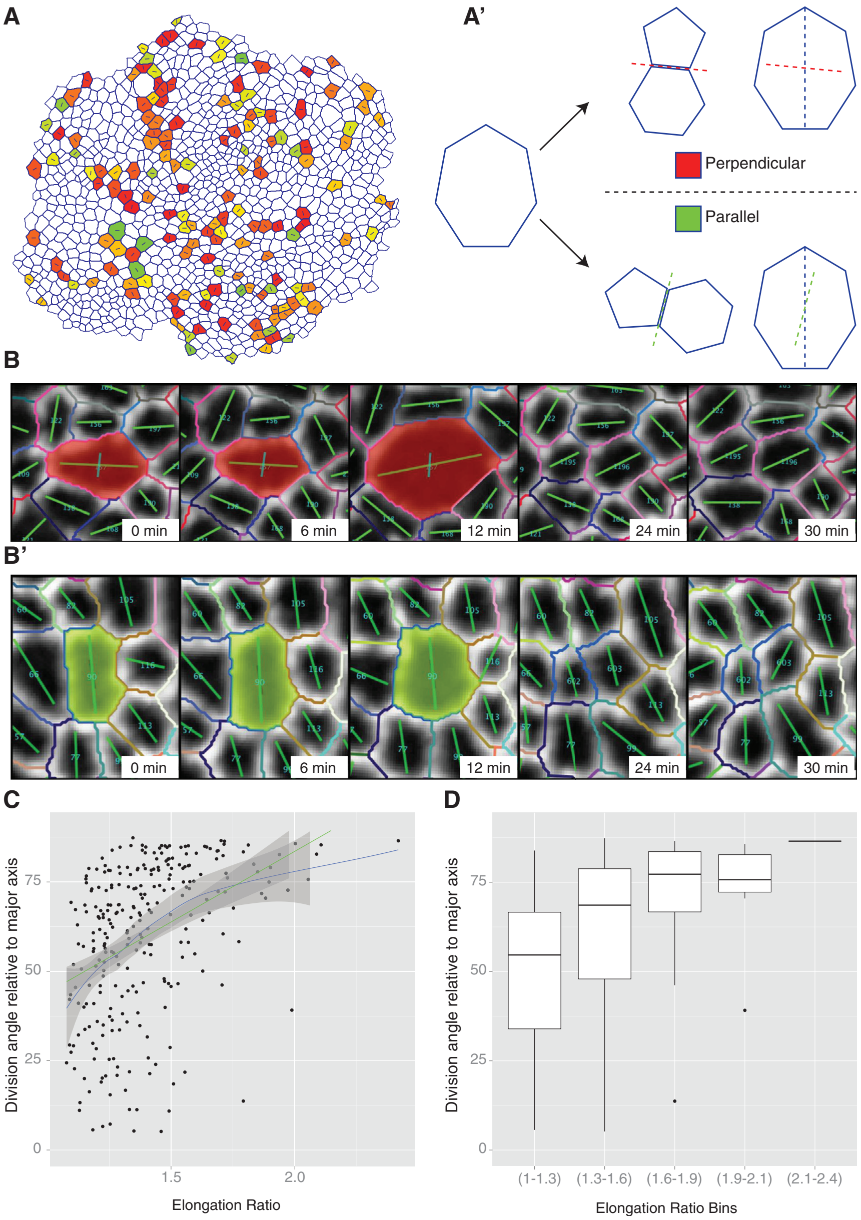

Figure 6. Control of Cell Division Orientation in the *Drosophila* Wing Disc

If not stated otherwise, error bars indicate SEM in all figures.

(A and A′) Overview of the orientation angle of the division axis relative to the long axis of the cells. Note that contrary to expectations, some cells divide parallel to the long axis (red equals fully perpendicular, green fully parallel).

(B and B′) Representative montages of a perpendicular division and a parallel division, respectively.

(C and D) The orientation of the division angle relative to the long axis of the cell depends partly on the aspect ratio of the dividing cells. Cells with a small aspect ratio show more variance, but there is a trend toward more perpendicular divisions as the aspect ratio increases. (C) Every black dot corresponds to one division statistic; green line is a simple linear model fit; blue line is an adaptive local polynomial regression; envelope indicates 0.95 confidence level. (D) Boxplot whiskers indicate 1.5× IQR, hinges IQR, and inner lines the median.

(Figures 6A′, 6C, and 6D). However, some cells do not obey this rule and divide to form the new junction parallel to the long axis of the dividing cell (Figures 6B′, 6C, and 6D). Although most of these cases are for cells that are not significantly elongated (elongation ratio less than 1.3), whereby ellipse fittings could be introducing errors in the estimation of the long axis, occasionally even significantly elongated cells can divide to bisect their short axis (Figure 6B′). Without an automated segmentation and unbiased high-throughput analysis method, it would have been difficult to identify such outliers, which may uncover additional, previously overlooked factors that regulate cell division orientation.

Drosophila Wing Disc Analysis II: Epithelial Junction Dynamics

Apart from quantifying cell geometries, the network abstraction created by the CellGraph plugin of EpiTools also allowed us to analyze the evolution of cellular neighborhood relationships during tissue development and detect any neighbor-exchange events, such as intercalations (also known as T1 transitions; Bertet et al., 2004; Farhadifar et al., 2007). Historically, it has been assumed that very few T1 transitions occur in the proliferating wing imaginal disc, as cells from the same lineage (clones) remain as intact clusters and do not disperse, suggesting that cells adhere tightly to their neighbors (Bryant, 1970; Garcia-Bellido et al., 1973; Resino et al., 2002). Previous attempts at manually tracking a few cells in the proliferating wing disc have also failed to detect significant cell rearrangements (Gibson et al., 2006). With our automated and systematic high-throughput analysis methods, we were able to detect a significant number of T1 transitions (Figure 7C), averaging at 13 transitions per 1,000 cells per hour over a 10-hr imaging window (an average total of 129 transitions in 1,000 cells over 10 hr) (Figure 7B). There were no significant changes in the frequency of T1 transitions over the 10-hr imaging window, suggesting that these transitions are not an artifact of the ex vivo culture. Upon analysis of the spatial distribution of these transitions across the epithelia, we could not detect any clear patterns of transition clustering or directionality (Figure 7A). We did find that for the four cells involved in a T1 transition, the pair that would gain an edge (winners) frequently started the transition as hexagons or pentagons, and would finish the transition as heptagons or hexagons, whereas the pair that would lose an edge (losers) would generally start as heptagons or hexagons and finish as hexagons or pentagons (Figures 7E and 7F). In other words, the cells that have a larger number of sides would "lose" an edge to the cells that have a lower number of sides

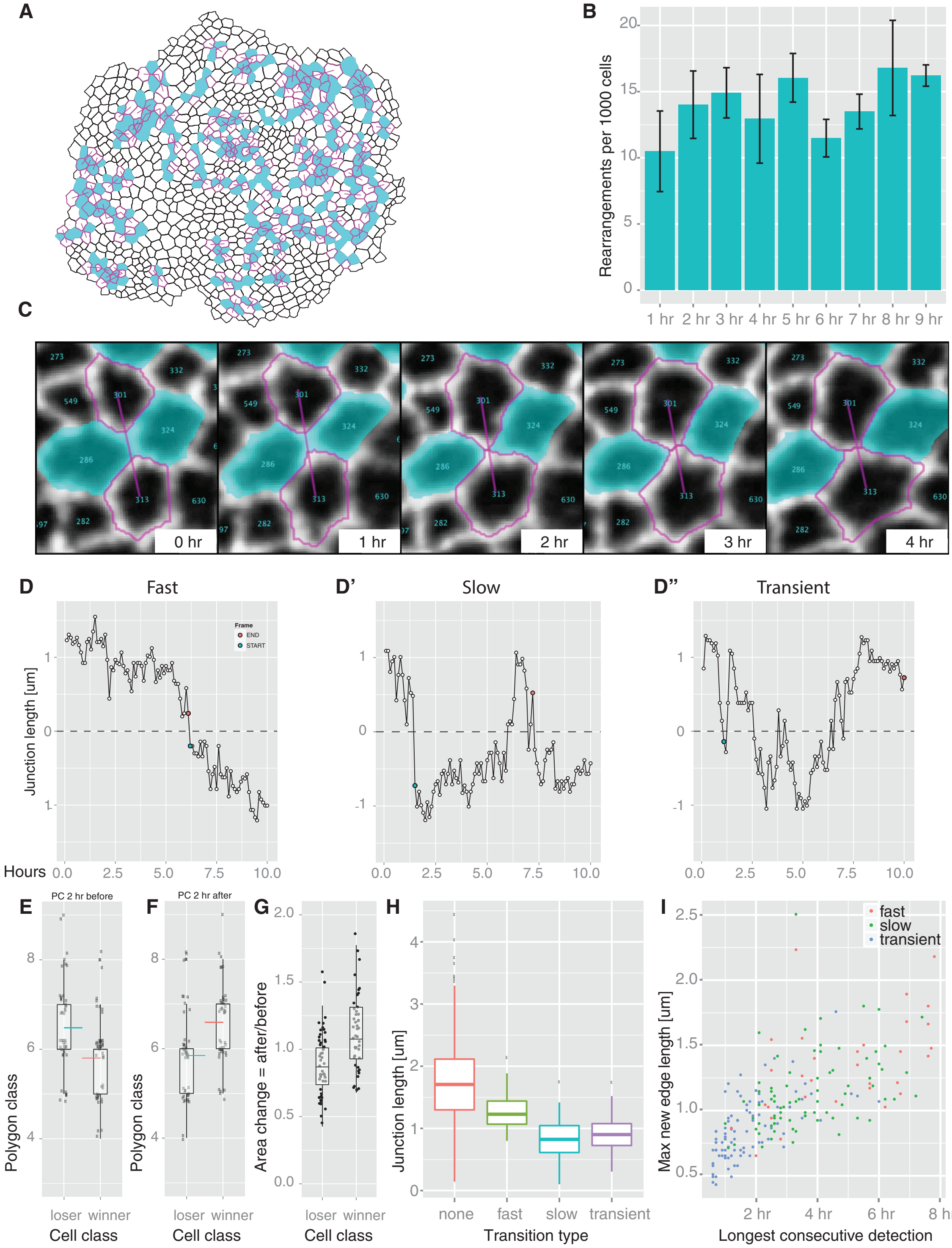

Figure 7. T1 Transitions in the *Drosophila* Wing Disc

If not stated otherwise, error bars indicate SEM in all figures. All boxplot whiskers indicate 1.5× IQR, hinges IQR, and inner lines the median.

(A) All T1 transitions detected over a 10-hr period traced back to the first time point. Linked magenta cells will intercalate in between cyan cells.

(B) Transition frequency remains constant during the duration of the imaging session.

(C) Representative montage of a T1 intercalation.

(D) T1 transitions follow different dynamics. Here we classified them into fast (D), slow (D′), and transient (D″). See Figure S6 for classification rules.

(E) Cells that will lose an edge during a transition are of a higher polygon class, on average 2 hr before the transition, than those that will gain an edge. Blue and red lines indicate the mean.

(legend continued on next page)

during a transition process. Consistent with Lewis' law (Lewis, 1928) the cells that gain an edge also increase their apical area after the transition, whereas cells that lose an edge are smaller after the transition (Figure 7G).

To gain a more quantitative understanding of the dynamics of these T1 transitions, we tracked the dynamic fluctuations of the length of each junction over 10 hr. As most junctions did not change their length significantly, we focused our analysis on the junctions that would shrink to a length of zero and then be substituted by a new growing junction (which we plot as negative values in Figure 7D). These are effectively T1 transitions. As a result of this analysis we noticed distinct "classes" of junctional dynamics. In an attempt to systematically classify these, we designed an algorithm to classify the transitions into three classes: fast, slow, and transient (Figures 7D, 7D″, and S6). In fast transitions (18% of total transitions), cells very efficiently exchange neighbors and the new neighborhood relationship remains stable. In slow transitions (37% of total transitions), the new neighborhood relationship eventually stabilizes, but takes longer to reach this stable state, while transient transitions (45% of total transitions) constantly fluctuate between the old and new neighborhood configurations. These definitions depend on the imaging window, but provide a method to quantify and classify the transition dynamics. In principle, if imaging windows were not limited, junctions would fluctuate between these dynamic states and show a continuum of behavior along this dynamic spectrum. On average, the longest junctions do not show any transitions (Figure 7H), but the junctions that undergo fast and decisive transitions are longer than the slow and transient transitions. Thus, it is not simply that longer junctions take longer to shrink to zero and grow again in the orthogonal direction, suggesting that the fast T1 transitions may have a separate mechanism of regulation. After the neighborhood exchange, the new junctions formed as a result of the fast and slow transitions grow to a longer length and remain stable for a much longer period of time than the transient transitions that fluctuate back and forth around the four-way vertex (Figure 7I). Whether these different classes of T1 transitions are fundamentally different in their regulation and function remains an interesting question for future research.

DISCUSSION

Advances in time-lapse imaging methods have resulted in very large datasets that are becoming impossible to analyze without robust quantitative tools. To address this pressing issue, we have created a new image analysis toolkit for epithelial tissues called EpiTools, which is aimed at biologists with little computer-science background, although the source code is also available should the user wish to extend or modify it for their own needs.

The main strength of EpiTools is its modularity. Splitting EpiTools into two parts gives our users more flexibility. The modular format of EpiTools Part 1 is designed for segmenting time-lapse images and outputting digitalized skeletons of cell outlines for further quantification, whereby users can use EpiTools Part 2 for, or their favorite existing software. Similarly, if users have already segmented their images with other software, they can use EpiTools Part 2 for further morphometric quantifications. The integration of EpiTools Part 2 into a larger bioimaging platform, Icy, that many users are already familiar with, makes it more accessible and user-friendly. Importantly, EpiTools allows for the easy manipulation of segmentation parameters, so that users can adapt the pipeline to the geometric idiosyncrasies of their biological system of choice. We believe these improved flexibility and user-friendly features will ensure that more users will adopt EpiTools for their image segmentation, tracking, and quantification, which is in increasing demand with the current rise of time-lapse microscopy.

There have been other image segmentation and analysis software available, each with its own strengths and weaknesses. We have tried to summarize the different features of each in Table S1. This will hopefully allow users to decide which one best suits them. Indeed there is no software that fits all criteria. Our decision to develop a new set of tools rather than to rely on previously published techniques was due to multiple reasons. Closed source code base (Packing Analyzer), and/or requirements for specific hardware (MorphoGraphX), excluded some solutions. Furthermore, the apical localization of the junctional marker E-cadherin and the limited tissue penetration also denied the use of volumetric-based methods such as EDGE or EDGE4D. SeedWaterSegmenter offered good segmentation performance but was problematic for projection and curation of long time series. A major drawback of all discovered solutions (Table S1) was also the lack of native interfaces to known imaging platforms such as FIJI (ImageJ) or Icy. We valued the latter because we think that exploratory analysis must be assisted by known, reliable, and easy interfaces. Powerful visualization features and easy image interaction result in much more intuitive data exploration for the scientist. In line with this argument we also concentrated our efforts on generalizing the EpiTools toolbox enough to allow widespread adoption. The image-processing part (MATLAB) does not require specific data dimensionality or format (e.g. 3D, 2D, time) through use of the bioformats library, ensuring that the user can start from multiple entry points. Parameter choice, which is usually not retained between iterations and leads to difficult decision processes, is aided by an easy GUI. Here we allow the user to review and choose among several runs of the same function. Finally, we simplified the setup procedure. Indeed we noticed that advanced installation procedures, while obvious to the creators, are a major deterrent for

(F) Cells that gain an edge during a transition are of a higher polygon class, on average 2 hr after the transition, relative to cells that lost an edge. Blue and red lines indicate the mean.

(G) Transitions are associated with a change in cell area. On average, the area of cells that lose an edge decreases after the transition while it increases for the cells that gained an edge.

(H) Cells that do not undergo transitions have longer junctions than transitioning cells. Furthermore, rapidly transitioning cells have longer junctions than slowly or transiently transitioning cells.

(I) Rapidly transitioning junctions reach a greater length and are more stable than slowly or transiently transitioning junctions.

widespread adoption (e.g. in TTT, itk/vtk custom compilation, and EDGE3D). Thus, the setup procedure was simplified to allow a simple drag-and-drop procedure without compilation of additional libraries.

With the current version of EpiTools, we have primarily analyzed the epithelial growth dynamics of the *Drosophila* wing imaginal disc, and reproduced data in agreement with previous work, such as the polygonal packing patterns of the epithelia in different cell populations (Gibson et al., 2006, 2014). We also noticed that although most cells divide to bisect the long axis of the dividing mother cell, as previously reported in the wing disc (Gibson et al., 2011; Mao et al., 2011) and other systems (Hertwig, 1893; Morin and Bellaïche, 2011; Ragkousi and Gibson, 2014), there was also a significant population of cells that did not obey this rule. Understanding the nature of such divisions, and attempting to distinguish whether it is cell geometry (shape) (Minc et al., 2011; Wyatt et al., 2015), the sensing of tension anisotropy of the cell (Campinho et al., 2013; Fink et al., 2011; Mao et al., 2013), or the effect of neighboring cell topologies (Gibson et al., 2011), will be easier to pursue with the dynamic quantitative tools now available in EpiTools and its combination with force-inference methods such as CellFIT (Brodland et al., 2014).

We also analyzed the dynamic patterns of cell rearrangements (T1 transitions) and junctional fluctuations in the wing disc, and revealed that the junctions are more mobile and the tissue more fluid-like than previously thought. As there appears to be no clear spatial patterns and orientations to these T1 transitions, it is unclear whether they have a functional significance or whether they are just passive consequences of tissue homeostasis. The fact that it is consistently the cells that have a larger number of sides that "lose" an edge to the cells that have a smaller number of sides does suggest that T1 transitions may have a role in maintaining the conserved polygonal packing geometry observed in many epithelia (Gibson et al., 2006) and, perhaps, buffer heterogeneities induced by cell divisions (Figures 5E and 5E′). Without regulated T1 transitions, cells would either always adhere tightly to their original neighbors or intercalate too freely, neither of which would allow the necessary mechanical tensions and cell geometries to emerge in the tissue to pattern cell divisions and tissue growth (Legoff et al., 2013; Mao et al., 2013).

Purely based on tissue dynamics, it is of course unclear how these T1 transitions are regulated. If this process were driven purely by a "passive" force equilibration process, one would expect the longest junctions to be the most stable, which is true to a certain extent (Figure 7). However, the fastest (and most stable/irreversible) T1 transitions actually occur in junctions that are normally longer than the slow/transient T1 transitions, suggesting that there may be a more active mechanism at play in regulating these T1 transitions. Extensive studies of T1 transitions in the *Drosophila* embryo have shown that a cell-autonomous accumulation of non-muscle Myosin II at the shrinking junctions during the first phase of the transition is required for the increase in cortical tension and shortening of that junction (Bertet et al., 2004; Rauzi et al., 2008; Zallen and Wieschaus, 2004). However, extrinsic forces can also induce cell rearrangements (Aigouy et al., 2010; Sugimura and Ishihara, 2013). Future studies of cortical tension and Myosin II dynamics may therefore be needed to assess the regulatory mechanism and possible function of T1 transitions in the wing imaginal disc.

Limitations

We have designed EpiTools so that it supports most imaging file formats, but there are a few limitations. EpiTools Part 1 accepts 8- or 16-bit bioformat compatible images with two additional requirements: (1) information regarding one time point cannot be distributed across multiple files; (2) the used file extension has to be included in the user-settings file (for more information see Supplemental Experimental Procedures). The preferred format is single TIFF file for every time point. For EpiTools Part 2, skeleton files should be 8-bit binary images. Again, the preferred format is TIFF.

The major limitation of EpiTools is that in its present form our toolbox is not suited for volumetric 3D analysis. We accept that biological datasets are too heterogeneous to allow for a unique solution for data processing and analysis, hence different software is required (Table S1). As the data quality from volumetric 3D imaging improves, we aim to add full 3D volumetric analysis to our toolkit. The modular and open-source nature of EpiTools makes it an ideal platform to develop new features.

In summary, we have generated a series of accessible tools aimed at harnessing recent advances in optical microscopy to produce a quantitative description of epithelial tissue morphogenesis. We anticipate that these tools will greatly facilitate the study of tissue dynamics in development and disease.

EXPERIMENTAL PROCEDURES

License Information

To encourage the sharing of resources, EpiTools is published under an open-source (GPLv3) license, which can be downloaded from http://tiny.uzh.ch/mM.

Live Imaging

Wing discs were cultivated ex vivo and imaged as described by Zartman et al. (2013). However, the discs were not encapsulated in an alginate gel, as we have found that this step can be omitted without negatively affecting the imaging. A total of 3 E-cadherin:GFP-expressing wing discs (Huang et al., 2009) were imaged over 10 hr each, from around 100 hr after egg laying.

Segmentation Parameters

The parameters used for segmenting the wing disc time lapses and other images shown in Figure S3 are shown in Table 1.

Measurement of the Division Orientation

We define the division orientation as the angle between the longest axis of a mother cell before division and the new junction between the two daughter cells after division. To reliably measure the angle, we decided to average multiple temporal combinations such that individual frame differences would not affect our result. The longest elongation axis of a mother cell was retrieved using five time points from 72 min to 42 min before the division when the two daughter cells are first visible (the acquisition interval was 6 min). The reason to exclude the time points in the immediate vicinity of the division (i.e. 36 min to 6 min before) was to avoid including the apical rounding phase of mitosis whereby the increasingly circular cell shape makes the longest elongation estimation unreliable. The new junction was also measured in five time points after the division. Specifically for each frame the segment between the two centroids of the daughter cells was computed, and the angle of the new junction was computed as being perpendicular to this segment. The final average value for the division orientation is the mean of the 25 possible combinations.

SUPPLEMENTAL INFORMATION

Supplemental Information includes Supplemental Experimental Procedures, six figures, one table, and source code and can be found with this article online at http://dx.doi.org/10.1016/j.devcel.2015.12.012.

AUTHOR CONTRIBUTIONS

D.H. designed and implemented EpiTools Part 2, analyzed the data, and wrote the article. A.H. designed and implemented the image-processing algorithms for EpiTools Part 1, and wrote the article. S.R. performed the biological experiments, designed EpiTools Part 2, analyzed the data, and wrote the article. L.G. implemented EpiTools Part 1 GUI, analyzed the data, and wrote the article. A.L.T. designed and implemented the image-processing algorithms for EpiTools Part 1. N.T. and K.B. designed the research. Y.M. designed the research, analyzed the data, and wrote the article.

ACKNOWLEDGMENTS

We thank Rob Tetley (Mao lab) for providing the embryo image and John Davis (Nic Tapon lab) and Federica Mangione (Enrique Martin-Blanco lab) for the histoblast image. We thank Maria Duda and Rob Tetley from the Mao lab for providing invaluable feedback during the testing of EpiTools. We thank Alexandre Dufour for helpful discussions on the Icy plugins. We thank members of the Mao lab, Franck Pichaud and David Ish-Horowicz, for critical reading of the manuscript. Y.M. is funded by a Medical Research Council Fellowship MR/L009056/1. A.T. and N.T. are funded by Cancer Research UK. D.H., S.R., L.G., and K.B. are funded by the Kanton of Zürich and the Swiss National Science Foundation.

Received: June 7, 2015
Revised: November 10, 2015
Accepted: December 9, 2015
Published: January 11, 2016

REFERENCES

Aegerter-Wilmsen, T., Heimlicher, M.B., Smith, A.C., de Reuille, P.B., Smith, R.S., Aegerter, C.M., and Basler, K. (2012). Integrating force-sensing and signaling pathways in a model for the regulation of wing imaginal disc size. Development *139*, 3221–3231.

Aigouy, B., Farhadifar, R., Staple, D.B., Sagner, A., Röper, J.-C., Jülicher, F., and Eaton, S. (2010). Cell flow reorients the axis of planar polarity in the wing epithelium of *Drosophila*. Cell *142*, 773–786.

Aldaz, S., Escudero, L.M., and Freeman, M. (2010). Live imaging of *Drosophila* imaginal disc development. Proc. Natl. Acad. Sci. USA *107*, 14217–14222.

Barbier de Reuille, P., Routier-Kierzkowska, A.L., Kierzkowski, D., Bassel, G.W., Schupbach, T., Tauriello, G., Bajpai, N., Strauss, S., Weber, A., Kiss, A., et al. (2015). MorphoGraphX: a platform for quantifying morphogenesis in 4D. Elife *4*, 05864.

Bertet, C., Sulak, L., and Lecuit, T. (2004). Myosin-dependent junction remodelling controls planar cell intercalation and axis elongation. Nature *429*, 667–671.

Blanchard, G.B., Kabla, A.J., Schultz, N.L., Butler, L.C., Sanson, B., Gorfinkiel, N., Mahadevan, L., and Adams, R.J. (2009). Tissue tectonics: morphogenetic strain rates, cell shape change and intercalation. Nat. Methods *6*, 458–464.

Brandes, U., Eiglsperger, M., Herman, I., Himsolt, M., and Marshall, M.S. (2002). GraphML progress report structural layer proposal. In Graph drawing, P. Mutzel, M. Jünger, and S. Leipert, eds. (Springer), pp. 501–512.

Brodland, G.W., Veldhuis, J.H., Kim, S., Perrone, M., Mashburn, D., and Hutson, M.S. (2014). CellFIT: a cellular force-inference toolkit using curvilinear cell boundaries. PLoS One *9*, e99116.

Bryant, P.J. (1970). Cell lineage relationships in the imaginal wing disc of *Drosophila melanogaster*. Dev. Biol. *22*, 389–411.

Campinho, P., Behrndt, M., Ranft, J., Risler, T., Minc, N., and Heisenberg, C.-P. (2013). Tension-oriented cell divisions limit anisotropic tissue tension in epithelial spreading during zebrafish epiboly. Nat. Cell Biol. *15*, 1405–1414.

Cilla, R., Mechery, V., Hernandez De Madrid, B., Del Signore, S., Dotu, I., and Hatini, V. (2015). Segmentation and tracking of adherens junctions in 3D for the analysis of epithelial tissue morphogenesis. PLoS Comput. Biol. *11*, e1004124.

de Chaumont, F., Dallongeville, S., Chenouard, N., Hervé, N., Pop, S., Provoost, T., Meas-Yedid, V., Pankajakshan, P., Lecomte, T., Le Montagner, Y., et al. (2012). Icy: an open bioimage informatics platform for extended reproducible research. Nat. Methods *9*, 690–696.

D'Errico, J. (2006). Surface fitting using gridfit. http://ch.mathworks.com/matlabcentral/fileexchange/8998-surface-fitting-using-gridfit.

Escudero, L.M., Bischoff, M., and Freeman, M. (2007). Myosin II regulates complex cellular arrangement and epithelial architecture in *Drosophila*. Dev. Cell. *13*, 717–729.

Farhadifar, R., Röper, J.-C., Aigouy, B., Eaton, S., and Jülicher, F. (2007). The influence of cell mechanics, cell-cell interactions, and proliferation on epithelial packing. Curr. Biol. *17*, 2095–2104.

Fink, J., Carpi, N., Betz, T., Bétard, A., Chebah, M., Azioune, A., Bornens, M., Sykes, C., Fetler, L., Cuvelier, D., et al. (2011). External forces control mitotic spindle positioning. Nat. Cell Biol. *13*, 771–778.

Galland, R., Grenci, G., Aravind, A., Viasnoff, V., Studer, V., and Sibarita, J.B. (2015). 3D high- and super-resolution imaging using single-objective SPIM. Nat. Methods *12*, 641–644.

Garcia-Bellido, A., Ripoll, P., and Morata, G. (1973). Developmental compartmentalisation of the wing disk of *Drosophila*. Nat. New Biol. *245*, 251–253.

Gelbart, M.A., He, B., Martin, A.C., Thiberge, S.Y., Wieschaus, E.F., and Kaschube, M. (2012). Volume conservation principle involved in cell lengthening and nucleus movement during tissue morphogenesis. Proc. Natl. Acad. Sci. USA *109*, 19298–19303.

Gibson, M.C., Patel, A.B., Nagpal, R., and Perrimon, N. (2006). The emergence of geometric order in proliferating metazoan epithelia. Nature *442*, 1038–1041.

Gibson, W.T., Veldhuis, J.H., Rubinstein, B., Cartwright, H.N., Perrimon, N., Brodland, G.W., Nagpal, R., and Gibson, M.C. (2011). Control of the mitotic cleavage plane by local epithelial topology. Cell *144*, 427–438.

Gibson, W.T., Rubinstein, B.Y., Meyer, E.J., Veldhuis, J.H., Brodland, G.W., Nagpal, R., and Gibson, M.C. (2014). On the origins of the mitotic shift in proliferating cell layers. Theor. Biol. Med. Model *11*, 26.

Gunduz, C., Yener, B., and Gultekin, S.H. (2004). The cell graphs of cancer. Bioinformatics *20* (*Suppl 1*), i145–i151.

Handke, B., Szabad, J., Lidsky, P.V., Hafen, E., and Lehner, C.F. (2014). Towards long term cultivation of *Drosophila* wing imaginal discs in vitro. PLoS One *9*, e107333.

Hertwig, O. (1893). Über den Werth der ersten Furchungszellen für die Organbildung des Embryos. Experimentelle Studien am Frosch und Tritonei. Archiv für mikroskopische Anatomie *42*, 662–807.

Huang, J., Zhou, W., Dong, W., Watson, A.M., and Hong, Y. (2009). From the Cover: directed, efficient, and versatile modifications of the *Drosophila* genome by genomic engineering. Proc. Natl. Acad. Sci. USA *106*, 8284–8289.

Khan, Z., Wang, Y.-C., Wieschaus, E.F., and Kaschube, M. (2014). Quantitative 4D analyses of epithelial folding during *Drosophila* gastrulation. Development *141*, 2895–2900.

Kierzkowski, D., Nakayama, N., Routier-Kierzkowska, A.-L., Weber, A., Bayer, E., Schorderet, M., Reinhardt, D., Kuhlemeier, C., and Smith, R.S. (2012). Elastic domains regulate growth and organogenesis in the plant shoot apical meristem. Science *335*, 1096–1099.

Krzic, U., Gunther, S., Saunders, T.E., Streichan, S.J., and Hufnagel, L. (2012). Multiview light-sheet microscope for rapid in toto imaging. Nat. Methods *9*, 730–733.

Lecuit, T., and Le Goff, L. (2007). Orchestrating size and shape during morphogenesis. Nature *450*, 189–192.

Legoff, L., Rouault, H., and Lecuit, T. (2013). A global pattern of mechanical stress polarizes cell divisions and cell shape in the growing *Drosophila* wing disc. Development *140*, 4051–4059.

Lewis, F.T. (1928). The correlation between cell division and the shapes and sizes of prismatic cells in the epidermis of cucumis. Anat. Rec. *38*, 341–376.

Liu, M., Yadav, R.K., Roy-Chowdhury, A., and Reddy, G.V. (2010). Automated tracking of stem cell lineages of *Arabidopsis* shoot apex using local graph matching. Plant J. *62*, 135–147.

Mao, Y., Tournier, A.L., Bates, P.A., Gale, J.E., Tapon, N., and Thompson, B.J. (2011). Planar polarization of the atypical myosin Dachs orients cell divisions in *Drosophila*. Genes Dev. *25*, 131–136.

Mao, Y., Tournier, A.L., Hoppe, A., Kester, L., Thompson, B.J., and Tapon, N. (2013). Differential proliferation rates generate patterns of mechanical tension that orient tissue growth. EMBO J. *32*, 2790–2803.

Marinari, E., Mehonic, A., Curran, S., Gale, J., Duke, T., and Baum, B. (2012). Live-cell delamination counterbalances epithelial growth to limit tissue overcrowding. Nature *484*, 542–545.

Mashburn, D.N., Lynch, H.E., Ma, X., and Hutson, M.S. (2012). Enabling user-guided segmentation and tracking of surface-labeled cells in time-lapse image sets of living tissues. Cytometry *81*, 409–418.

Minc, N., Burgess, D., and Chang, F. (2011). Influence of cell geometry on division-plane positioning. Cell *144*, 414–426.

Morin, X., and Bellaïche, Y. (2011). Mitotic spindle orientation in asymmetric and symmetric cell divisions during animal development. Dev. Cell. *21*, 102–119.

Osterfield, M., Du, X., Schüpbach, T., Wieschaus, E., and Shvartsman, S.Y. (2013). Three-dimensional epithelial morphogenesis in the developing *Drosophila* egg. Dev. Cell. *24*, 400–410.

Ragkousi, K., and Gibson, M.C. (2014). Cell biology in development: cell division and the maintenance of epithelial order. J. Cell Biol. *207*, 181–188.

Rauzi, M., Verant, P., Lecuit, T., and Lenne, P.-F. (2008). Nature and anisotropy of cortical forces orienting *Drosophila* tissue morphogenesis. Nat. Cell Biol. *10*, 1401–1410.

Resino, J., Salama-Cohen, P., and García-Bellido, A. (2002). Determining the role of patterned cell proliferation in the shape and size of the *Drosophila* wing. Proc. Natl. Acad. Sci. USA *99*, 7502–7507.

Sage, D. (2012). MIJ: making interoperability between ImageJ and Matlab possible. http://imagejconf.tudor.lu/program/poster/makinginter.

Shraiman, B.I. (2005). Mechanical feedback as a possible regulator of tissue growth. Proc. Natl. Acad. Sci. USA *102*, 3318–3323.

Sommer, C., Strähle, C., Köthe, U., and Hamprecht, F.A. (2011). ilastik: interactive learning and segmentation toolkit. Eighth IEEE International Symposium on Biomedical Imaging (ISBI) Proceedings (IEEE), pp. 230–233.

Sugimura, K., and Ishihara, S. (2013). The mechanical anisotropy in a tissue promotes ordering in hexagonal cell packing. Development *140*, 4091–4101.

Sweeton, D., Parks, S., Costa, M., and Wieschaus, E. (1991). Gastrulation in *Drosophila*: the formation of the ventral furrow and posterior midgut invaginations. Development *112*, 775–789.

Thevenaz, P., Ruttimann, U.E., and Unser, M. (1998). A pyramid approach to subpixel registration based on intensity. IEEE Trans. Image Process. *7*, 27–41.

Wyatt, T.P.J., Harris, A.R., Lam, M., Cheng, Q., Bellis, J., Dimitracopoulos, A., Kabla, A.J., Charras, G.T., and Baum, B. (2015). Emergence of homeostatic epithelial packing and stress dissipation through divisions oriented along the long cell axis. Proc. Natl. Acad. Sci. USA *112*, 5726–5731.

Zallen, J.A., and Wieschaus, E. (2004). Patterned gene expression directs bipolar planar polarity in *Drosophila*. Dev. Cell. *6*, 343–355.

Zartman, J., Restrepo, S., and Basler, K. (2013). A high-throughput template for optimizing *Drosophila* organ culture with response-surface methods. Development *140*, 667–674.

Developmental Cell, Volume *36*

Supplemental Information

EpiTools: An Open-Source Image Analysis

Toolkit for Quantifying Epithelial Growth Dynamics

Davide Heller, Andreas Hoppe, Simon Restrepo, Lorenzo Gatti, Alexander L. Tournier, Nicolas Tapon, Konrad Basler, and Yanlan Mao

INVENTORY OF SUPPLEMENTAL INFORMATION

Supplemental Figures

Figure S1, related to Figure 3. EpiTools Complete workflow
Figure S2, related to Figure 4. EpiTools Icy CellOverlay examples
Figure S3, related to Figure 2. Different epithelia analyzed using EpiTools.
Figure S4, related to Figure 6. Ommatidial cell rearrangements in Drosophila eye imaginal disc
Figure S5, related to Figure 5. Cell delaminations in the Drosophila wing disc
Figure S6, related to Figure 7. Flowchart for the identification and classification of T1 transitions in the wing disc samples.

Supplemental Tables
Table S1, related to Figure 1. Comparison of existing image segmentation and tracking software

Supplemental Experimental Procedures
Source code
EpiTools Part 1a: Matlab and C++ modules
EpiTools Part 1b: EpiTools-Matlab-GUI
EpiTools plugins for Icy
Supported file formats
Quick Guides for EpiTools

Supplemental References

Figure S1

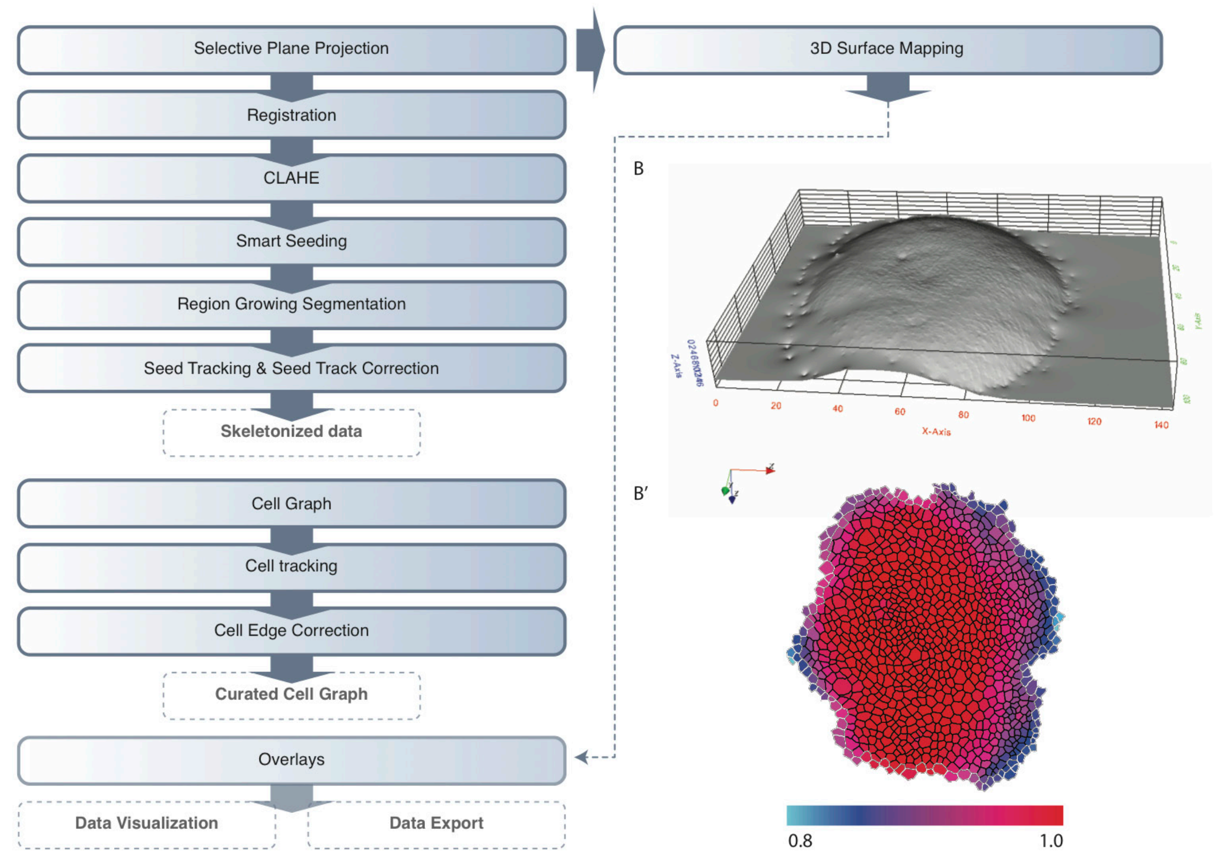
A
Selective Plane Projection
Registration
CLAHE
Smart Seeding
Region Growing Segmentation
Seed Tracking & Seed Track Correction
Skeletonized data
Cell Graph
Cell tracking
Cell Edge Correction
Curated Cell Graph
Overlays
Data Visualization
Data Export
3D Surface Mapping
B
0
20
40
60
80
100
120
140
X-Axis
Z-Axis
B′
0.8
1.0

Figure S2

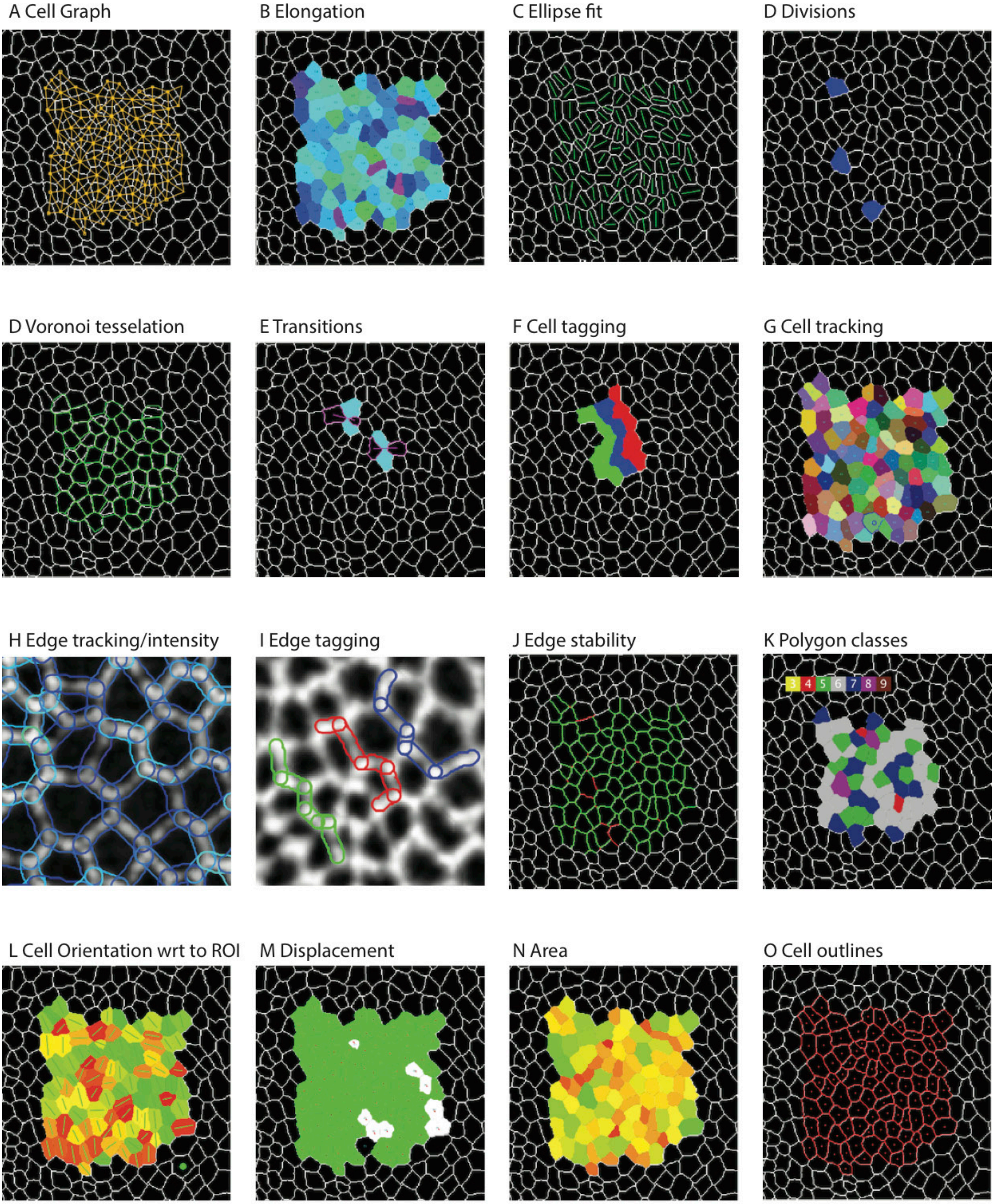

A Cell Graph
B Elongation
C Ellipse fit
D Divisions
D Voronoi tesselation
E Transitions
F Cell tagging
G Cell tracking
H Edge tracking/intensity
I Edge tagging
J Edge stability
K Polygon classes
3 4 5 6 7 8 9
L Cell Orientation wrt to ROI
M Displacement
N Area
O Cell outlines

Figure S3

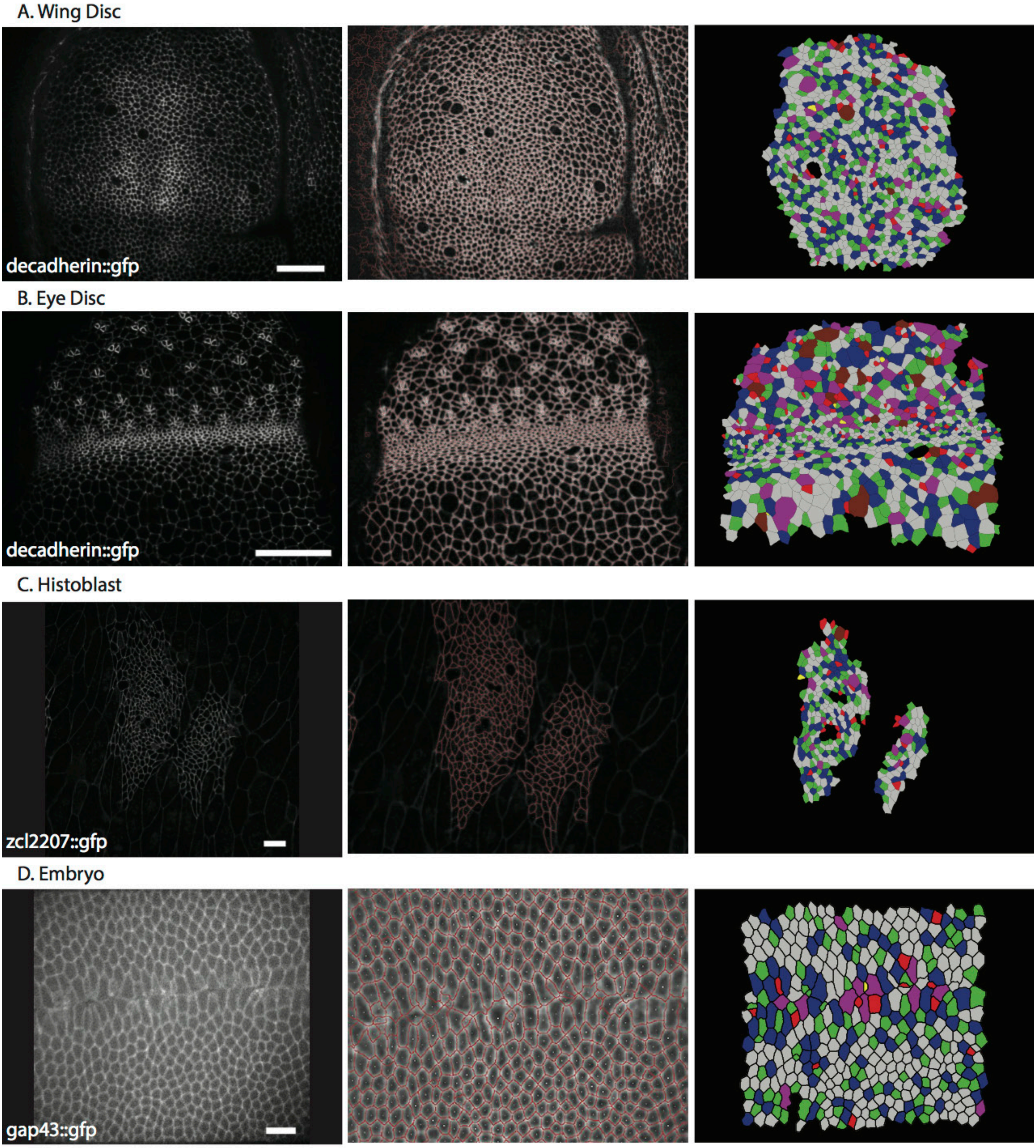

A. Wing Disc
decadherin::gfp
B. Eye Disc
decadherin::gfp
C. Histoblast
zcl2207::gfp
D. Embryo
gap43::gfp

Figure S4

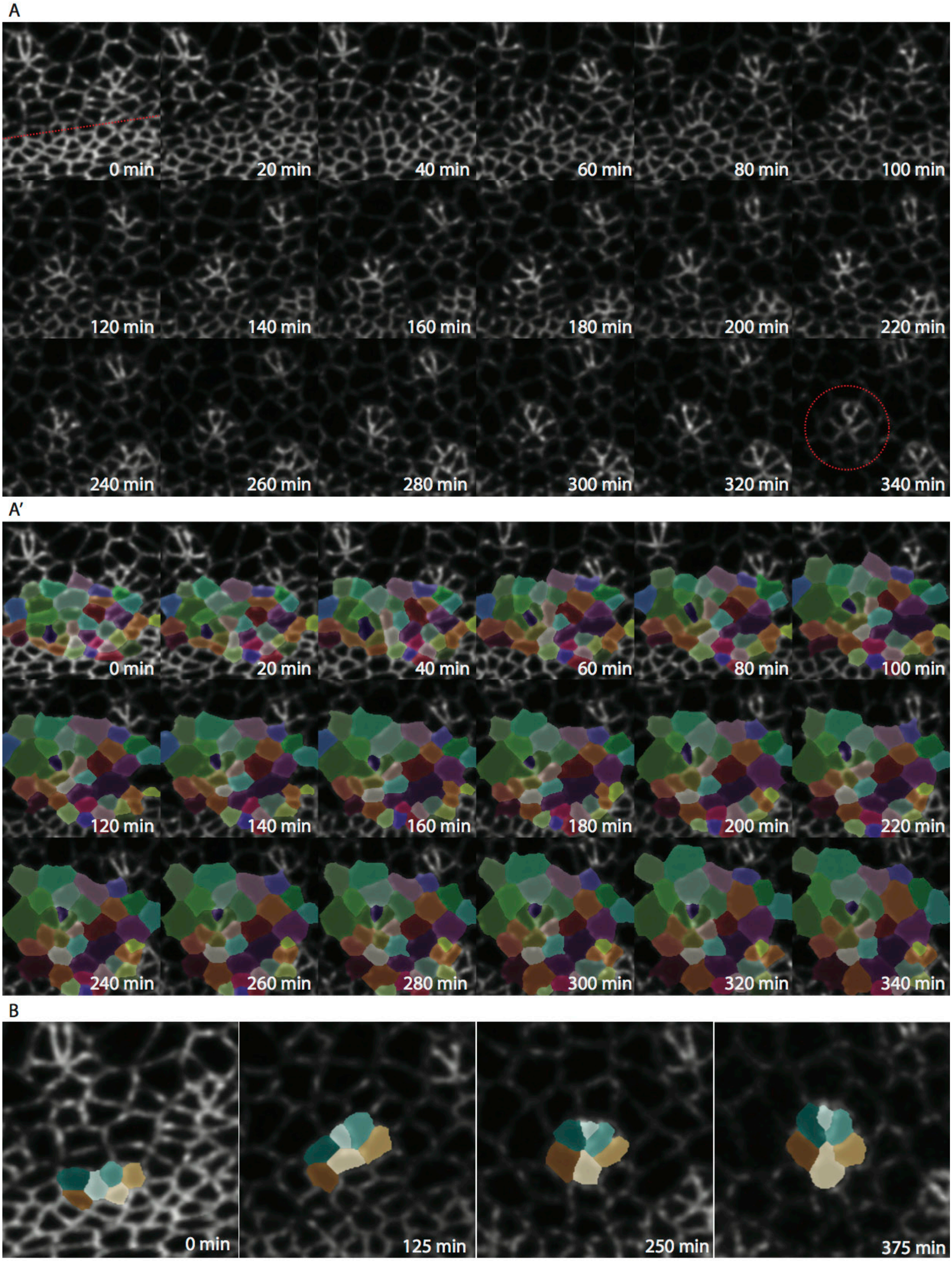
A
0 min
20 min
40 min
60 min
80 min
100 min
120 min
140 min
160 min
180 min
200 min
220 min
240 min
260 min
280 min
300 min
320 min
340 min
A′
0 min
20 min
40 min
60 min
80 min
100 min
120 min
140 min
160 min
180 min
200 min
220 min
240 min
260 min
280 min
300 min
320 min
340 min
B
0 min
125 min
250 min
375 min

Figure S5

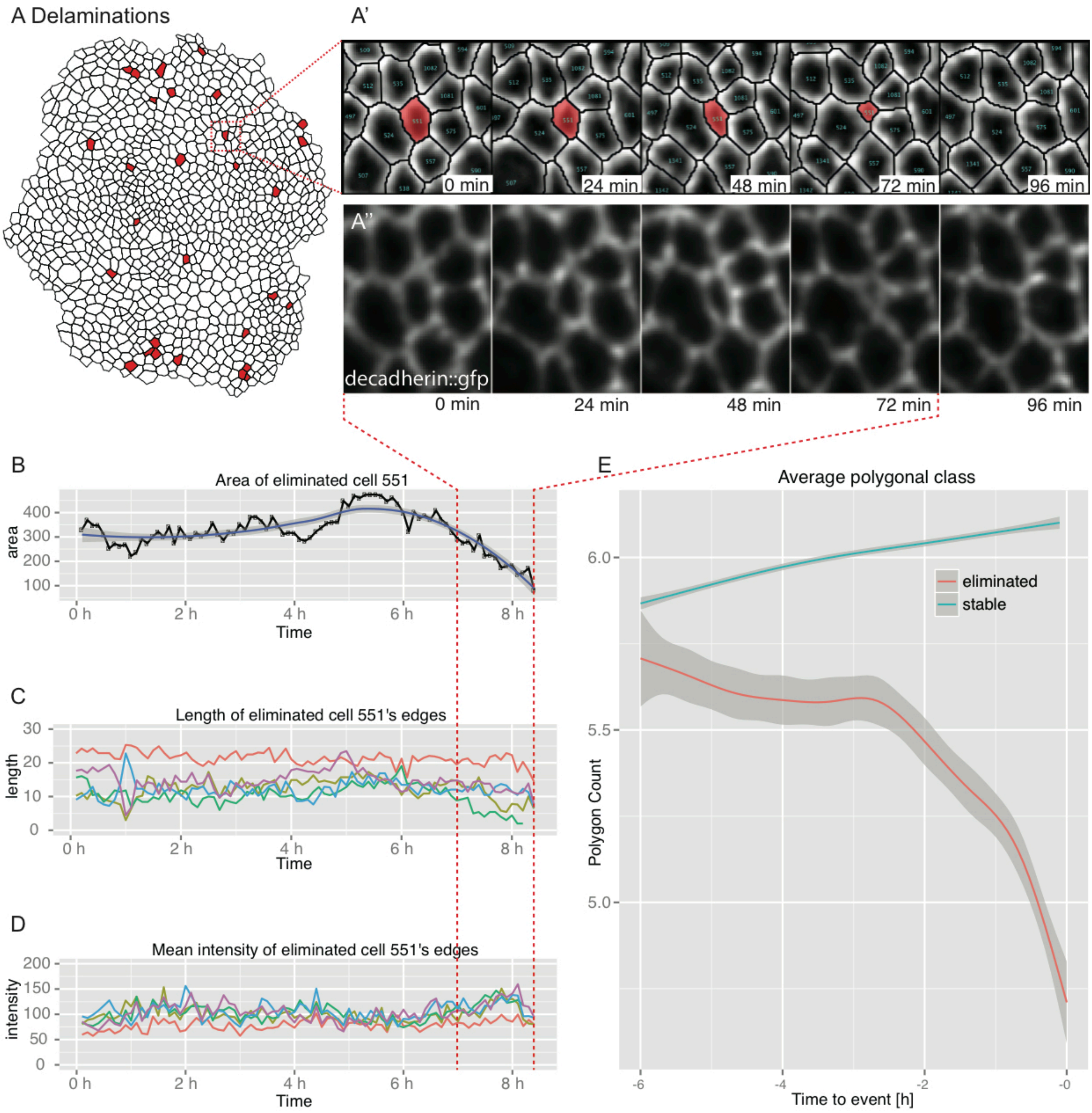
A Delaminations
A'
0 min
24 min
48 min
72 min
96 min
A''
decadherin::gfp
B
Area of eliminated cell 551
area
400
300
200
100
0 h
2 h
4 h
6 h
8 h
Time
C
Length of eliminated cell 551's edges
length
30
20
10
0
D
Mean intensity of eliminated cell 551's edges
intensity
200
150
100
50
0
E
Average polygonal class
Polygon Count
6.0
5.5
5.0
eliminated
stable
-6
-4
-2
-0
Time to event [h]

Figure S6

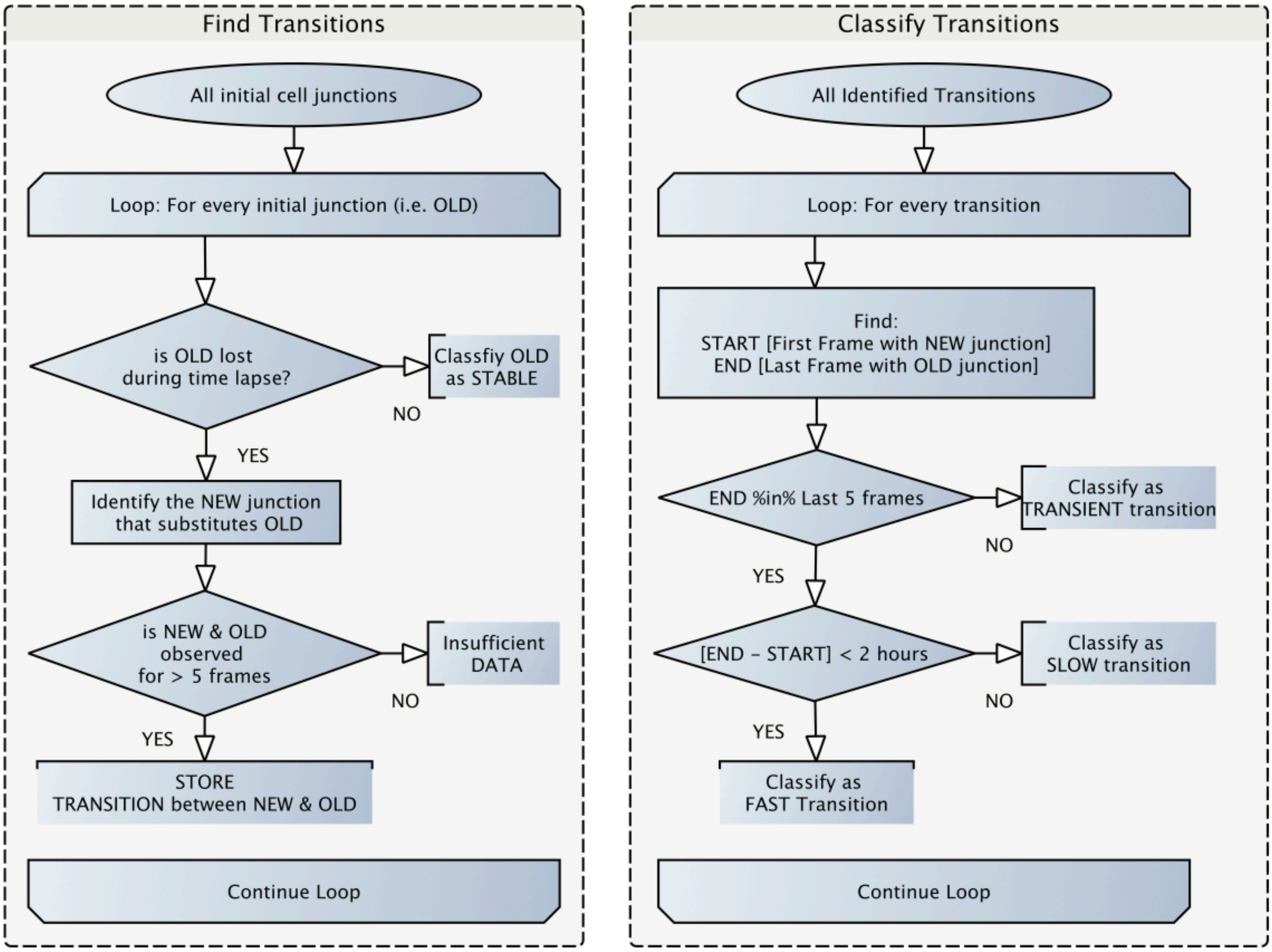
Find Transitions
All initial cell junctions
Loop: For every initial junction (i.e. OLD)
is OLD lost during time lapse?
NO
Classfiy OLD as STABLE
YES
Identify the NEW junction that substitutes OLD
is NEW & OLD observed for > 5 frames
NO
Insufficient DATA
YES
STORE TRANSITION between NEW & OLD
Continue Loop
Classify Transitions
All Identified Transitions
Loop: For every transition
Find: START [First Frame with NEW junction] END [Last Frame with OLD junction]
END %in% Last 5 frames
NO
Classify as TRANSIENT transition
YES
[END - START] < 2 hours
NO
Classify as SLOW transition
YES
Classify as FAST Transition
Continue Loop

Figure S1, related to Figure 3. EpiTools Complete workflow
(A) Flowchart describing the complete processing workflow for a 3D time lapse data set. First the Selective Plane Projection is applied to project individual time points on a 2D plane without interference from other membranes (e.g. the peripodial membrane in the imaginal wing discs). Second, the 2D image sequence is registered to correct for sample movement during the acquisition. Third, Contrast-Limited-Adaptive-Histogram-Equalization (CLAHE) is applied to even the intensity distribution across the image. Fourth, cells are localized through smart seed point identification to optimally initiate the segmentation step. Fifth, the segmentation procedure identifies the cell geometries through the region growing algorithm initiated from the seed locations. Six, the user can semi-automatically correct the segmentation result by placing or removing seed. The seeds are automatically connected through time to give feedback. If corrections were applied a Re-segmentation step (not shown) will integrate the modifications into the final segmentation. The results of the first part are the outlines of the cells, also called skeleton images.
The skeletonized time points are the input for the EpiTools plugins for the bioimaging platform Icy. First, the polygons representing each cell are identified and connected in space, i.e. a cell graph of neighborhood connectivity. Temporal linkage is added by graph based cell tracking, extending the previous data structure into a spatio-temporal graph. The user is still given possibility to correct the image skeletons by manually adding or removing cell edges through the CellEditor plugin. The curated Cell Graph is finally used to project informative overlays on the original image and/or derivatives, as well as exporting data for quantitative analysis.
(B) 3D Surface generated by the CellSurface plugin (part of the EpiTools plugins for Icy). EpiTools for Matlab saves a height map when the user applies the selective plane projection module. The map corresponds to the second, more detailed, surface estimation to locate the signal of interest. Files are saved within the analysis folder (/vtk). These exported files (.vtk) can be read by the CellSurface plugin, part of the EpiTools plugins for Icy. CellSurface generates a 3D Surface ROI leveraging on the 3D Mesh ROI plugin by Alexander Dufour (Dufour) and the VTK library http://www.vtk.org.
(B') Projection overlay, available through the CellOverlay plugin, visualizes as a color gradient the magnitude of the z-component from the surface normal of every cell. The overlay uses the estimated height map (see legend S1B) to assign z-coordinates to all coordinates of the cell's polygon. To compute the approximated 3D surface normal for every cell we use Newell's method (Ivan et al., 1974). The z-component of the surface normal is useful to estimate the area projection bias through the proportional dependence between 3D and projected 2D area (John and Alan, 1987). All values can be exported in an excel sheet through the layer options menu in Icy.

Figure S2, related to Figure 4. EpiTools Icy CellOverlay examples
Examples of the different types of data overlays available upon the release of EpiTools. More complete descriptions can be found on the EpiTools website.

Figure S3, related to Figure 2. Different epithelia analyzed using EpiTools.
Scale bar in all images are 20µm. (A) *Drosophila* wing imaginal disc labeled with Ecadherin, imaged with spinning disc microscopy. (B) *Drosophila* eye imaginal disc labeled with Ecadherin, imaged with spinning disc microscopy. (C) *Drosophila* histoblast nests labeled with ZCL2207 (ATPalpha subunit, septate junction marker), imaged with laser scanning confocal microscopy. (D) *Drosophila* embryo labeled with GAP43, imaged with spinning disc microscopy.

Figure S4, related to Figure 6. Ommatidial cell rearrangements in *Drosophila* eye imaginal disc
(A)Time-lapse of *ex vivo* imaging of ommatidial cell rearrangements during the development of the eye imaginal disc. After the morphogenetic furrow (red dashed line) passes, the ommatidial cells start differentiating into preclusters (inside the dashed red line).
(A') Epitools can be successfully employed to segment and track the differentiation of ommatidial cells.
(B) The tracking information can be used to follow cell rearrangements and trajectories during ommatidial differentiation.

Figure S5, related to Figure 5. Cell delaminations in the *Drosophila* wing disc
(A) Spatial distribution of live cell delaminations occurring over a 10 hour time period, *ex vivo*. A' Representative montage of a live cell delamination detected by Epitools. A'' The ecadherin:gfp signal remains constant throughout the process, a hallmark of live cell delamination.
(B) The area of cell 551 fluctuates randomly prior to the delamination.

(C) Epitools can track the evolution of the length of each edge of cell 551 prior to its delamination. There are no clear trends.
(D) The intensity of the ecadherin signal of each edge of cell 551 remains relatively constant until the delamination event.
(E) On average, delaminated cells see a decrease in their number of neighbors prior to being delaminated.

Figure S6, related to Figure 7. Flowchart for the identification and classification of T1 transitions in the wing disc samples.
The procedure is divided in two major parts. The first (Find Transitions) concerns the task of finding the transitions by analyzing the junctions of all cells. The second (Classify Transitions) classifies the identified transitions by analyzing the duration and persistence of the rearrangement.

<u>Find Transitions – Step 1</u>
Consider the set of all junctions in the first frame of the sample. For every edge the algorithm verifies whether the edge was lost at any point in time. If the edge is conserved throughout every time point it is classified as STABLE junction and not further analyzed. The remaining junctions are unstable and have thus at least 1 time point where they are not present. The algorithm identifies the edge that substituted the old edge by looking at the change in neighborhood relationships. If the change is preserved for more than 5 frames (threshold can be changed in the graphical user interface) the algorithm classifies the TRANSITION. A persistence with fewer than the set threshold is classified as INSUFFICENT to call a T1 transition.

<u>Classify transitions – Step 2</u>
Consider the set of all identified/found transitions by step 1. For every transition the algorithm identifies two variables: START, the first frame in which the new edge appeared, i.e. the edge that substituted the initial edge (old). END, the last frame in which the old edge is observed. Using these two variables we will classify the type of transition. If the frame END is one of the last 5 frames the transition is considered to be TRANSIENT. This decision is based on the idea that a stable transition should be observed for a sufficiently long time without reverting to the initial condition (old edge). The second condition is used to classify stable transitions according to how much time passed between the frame START and the frame END. The interpretation of this duration can be seen as the amount of time to stabilize the transition. We identified a threshold of 2 hours to define whether a transition should be defined FAST or SLOW.

Supplemental Tables

Table S1, related to Figure 1. Comparison of existing image segmentation and tracking software

Software	Language	First Tissue used	Dim	Open source	Setup	ImageJ Icy interface	Last update	Citation
Packing Analyzer	Java	DM Wing	2D	No	Easy	No	2012 /11	(Aigouy et al., 2010)
Seed-Water-Segmenter	Python	DM Amnio-serosa	2D	Yes	Medium	No	2015 /08	(Mashburn et al., 2012)
EDGE	Matlab Java	DM Embryo	3D	Yes	Medium	No	2012 /08	(Gelbart et al., 2012)
EDGE4D	C++	DM Embryo	3D	Yes	Advanced	No	2014 /03	(Khan et al., 2014)
TTT	C++	DM Notum	3D	Yes	Advanced	No	2015 (incom plete)	(Cilla et al., 2015)
Morpho-GraphX	C++ Cuda	AT Meristem	2.5D	Yes	Hardware required	No	2015 (contin uos)	(Kierzkowski et al., 2012)
Ilastik	Python	Machine Learning	2D/3 D	Yes	Easy	Cell Profiler	2015 (contin uos)	(Sommer et al., 2011)

Supplemental Experimental Procedures

Source Code

Source code for the current version of EpiTools is provided in SourceCode.zip. For updated versions please visit our website http://tiny.uzh.ch/dm and git repository https://bitbucket.org/davideheller/epitools/.

EpiTools Part 1a: Matlab and C++ modules

The image processing pipeline consists of a selective plane projection, image registration, automatic seed point generation, region growing segmentation from seed points (which conceptually represent cell centres), seed tracking and correction, followed by the final cell boundary segmentation. A detailed description of these steps can be found below.

Selective plane projection

Since many epithelial tissues grow as a curved surface, such as a dome in the case of the wing imaginal disc (Legoff et al., 2013), a simple maximum intensity projection along the z-axis of a confocal microscopy stack would capture any unwanted signals (such as the peripodial membrane in the wing disc) situated in a different focal plane (Fig. 2A). This is a typical problem with whole mount preparations. It was thus necessary to develop a projection method designed to follow the curvature of the tissue by fitting a surface to the epithelial topology. This was achieved through a two-stage projection approach. First a surface mesh was extracted from the image volume by fitting and interpolating scattered feature points (D'Errico, 2006), defined as voxels with strong image intensity. Although most feature points belonged to the desired layer's mesh, some of these points were 'outliers' and referred to signals in unwanted planes. Increasing the stiffness of the fitted surface would settle on the denser mesh of the desired layer, and thus remove most of the outliers. Only feature points with a strong intensity were used for this first step. In a second step, feature points that have a large deviation from the computed surface were removed. The surface fitting is repeated and can now be achieved with less stiffness and thus follow the curvature more accurately (Fig. 2A, green line). Finally, pixel intensities along the fitted surface were obtained which formed the projected image on which the subsequent image segmentation was performed. The 3D surface fit can be exported and used to correct subsequent geometric analyses, if necessary (Fig. S1).

Registration

Images are aligned (registered) at different time points using the image intensity distribution. Images were processed with a rigid transformation to find an optimal correlation between frames. The image registration step can also be performed through external software such as the StackReg plugin for ImageJ (Thevenaz et al., 1998) controlled via EpiTools using the MIJ interface (Sage, 2012).

Smart seeding

Using the selective plane projected image, the aim of this step is ideally to create a single seed point per cell from which to grow cellular regions (Fig. 2B). The use of seed points as a simple handle for each cell allows for very efficient adjustments. A single mouse click is enough to add, remove or fuse cellular regions. As long as the correct seeds are used, the final region-growing stage provides a sufficiently good segmentation result (Fig. 2C) and thus emphasizes the importance to generate accurate seed points during the initial stage (Fig. 2B). Seed points are often determined as the centroids of small homogeneous low signal regions. However, such an approach often leads to multiple seed points per cell (Fig. 2B, magenta arrows) as cells could contain several different homogeneous regions within its boundaries. Our seed point generation method was thus devised to include growing and merging of regions to reduce fragmentation: Homogeneous regions of a certain size below a rising signal intensity threshold (cell boundary signal) are identified and allocated to become new cellular regions with a unique identification (ID). Existing regions are grown by assimilating unallocated neighbouring pixels below a given intensity threshold. Each region is represented by a cluster of pixels with the same ID. While growing, neighbouring regions of small sizes and low intensities are merged to form larger regions. This approach ultimately provides images with, in most cases, just one seed point per cell (Fig. 2B, green arrows).

The initial seed finding could still produce multiple seed points especially in dividing cells due to the considerable increase in cell size in M phase. To reduce this duplication, an additional region-merging step was introduced. In this method, the intensities at the borders of adjacent regions were evaluated. Usually, the intensities of the borders of regions would be higher than the intensities in the centre of regions. In case of falsely identified borders, as often observed within large dividing cells, the border intensities of two adjacent regions would be similar or lower than the centre intensity of the region. Pixels are identified as similar or lower if their intensity is less than the intensity of the region's centre plus half its standard deviation. The percentage of such pixels within each border region is calculated and then used as a merging criterion. Finally, new seed points are calculated as centroid positions of merged regions (Fig. 2B).

Region growing segmentation
Once the seed points have been obtained, the cell region growing step is performed to provide accurate cell shape representations that precisely follow the contours of cell junctions (or membrane), as opposed to using linear approximations (Cilla et al., 2015). This is important because an accurate representation of the curvature of cell junctions is critical for understanding the mechanical properties of the cells (Brodland et al., 2014). The projection image is smoothed with a Gaussian kernel to reduce signal discontinuities at cell junctions. Cellular regions are grown from the seed points by assimilating neighbouring pixels below an increasing intensity threshold. The region growing is performed in parallel for each seed point and is guided by the local intensity flow, climbing up the intensity gradients that separate cells. This concurrent approach, whereby regions are grown at the same time in parallel, ensures that expanding regions meet at the ridge between cells where the cell junction is located. The growing is stopped locally once pixels from adjacent regions touch, which prevents spilling over or racing along the cell ridge resulting in discontinuities in the segmented cell boundaries. This combination of concurrent growing and local control results in an improved segmentation that more accurately follows the membrane contours, compared to the MATLAB watershed technique as shown in (Fig. 2C; (Meyer, 1994). This region growing approach can be re-applied after all seed corrections have been performed, at the final re-segmentation step, to provide an optimal segmentation.

Seed tracking and seed error correction
Seed tracking has been implemented primarily to facilitate the semi-automatic seed correction procedure. The tracking is performed on the cell seed positions obtained from the above segmentation steps. For each cellular position, a corresponding position is found in the vicinity in the previous frame (the vicinity parameter is expressed in pixel squared area around the seed). A missing seed point is identified by a broken track (Fig. 2D, magenta track) often due to segmentation errors, which are easily rectified and subsequently re-segmented. Each new track is allocated a track ID. The search space is initially small as the algorithm tries to find a corresponding position in the previous frame that has not moved far. The search space is increased at the next iteration to accommodate larger movements while excluding already allocated positions. The length of each track is recorded. The tracking has been combined with an innovative GUI to identify broken tracks and to add or delete seed points manually while continuing the tracking process. Cell division events can later be identified from broken tracks. A final re-segmentation can be applied on the corrected seed points to produce a final series of segmented frames, which can be exported as skeletons that accurately represent cell junction (or membrane) signals.

EpiTools Part 1b: EpiTools-Matlab-GUI

1. Component based software engineering

Component based software engineering. Component-based development EpiTools framework has been designed in compliance with component based software engineering (CBSE) standards. Therefore, we built a kernel of drivers which provide state communication layers, data retrieving methods, and dynamic result storage. Drivers are connected and assembled as modular stacks. Whether an element in the stack initiate a unilateral communication with a higher element in the stack configuration, an abstraction layer covers the element lower in the stack disposition. Layer abstraction increases flexibility in stack calls and data i/o. Analysis modules wrap multiple software components. Interaction between analysis modules and software framework is established via drivers which regulate, prioritise and manage connection to lower layers (i.e. data transportation between modules is realised via a spool list system which keeps track of the analysis modules successfully executed and the objects in output from the executed modules). Successful execution of an analysis module results both in objects creation (i.e. projected surface image files) and in tag exportation into the software instance

environment which is used as availability reference by successive analysis module execution. This system protects results retrieved from overwriting in case of multiple runs. Module execution is monitored by an executor driver which records both execution meta-data and parameter settings used for results retrieving. Parameter and meta-data storing enhances results reproducibility and error handling power.

2. Centralized computing

Client-Server Framework. Computational requests are elaborated through a first-in first-out (FIFO) en-queueing and de-queueing process. Every single command requested by the user or automatically generated by the software is first appended at the end of the execution list while submission to the process launcher will submit the first at the front of the execution list. This group of classes and methods composes the integrated queue & spool system which is responsible for the command execution, output object creation, tag exporting and environment releasing. Successfully executed analysis modules drive results objects (i.e. image files, text files or xml meta-data files) creation which is regulated by sandbox and storage protection drivers. These two subsystems link the physical location of created files with the program session in execution. File creation and tag exportation trigger a specific set of events which are listened by a class associated event manager. This events collecting system is linked to lower software layers through a communication channel initiated by the associated class which triggered the event (i.e. event-related procedure are required for synchronising and linking output objects with their availability status during EpiTools instance execution).

3. Extension interfaces

Input/Output drivers EpiTools framework interfaces other software platforms exporting output objects via standard and open file formats. This increases results portability and flexibility while it allows for integrating it in much bigger pipelines without intermediate file conversion steps. Importing and exporting processes are realised using OME Bio- Formats libraries (Linkert et al., 2010) linked to EpiTools through a Matlab wrapper provided by Open Microscopy Environment and custom adapted in our software implementation. User setting file, analysis descriptor files and meta-data files are written and read in clear-text XML format.

4. Graphical user interface

GUI Interface EpiTools comes with an elaborated GUI interfaces which enables the user to interact directly with the framework, modify the analysis execution and manage the import/export process of images, meta-data and results. Users without coding experience are then easily guided through the software through auto-explanatory forms with assisted and personalised help functions. EpiTools GUI is specifically designed to overcome definite processing requests - i.e. manual seed tracking or image cropping. Non-GUI functions benefit of EpiTools GUI interface since they gain interactivity and graphical display methods inherited from the generic graphical framework. Main forms are generated using Matlab GUI libraries which consists of code wrappers around Java Swing GUI libraries. Extensions of the built-in components have been realised via direct access to Java Swing libraries and JIDE classes (i.e. JTree, JTable, JTreeTable). Implementing the connection to these libraries required Matlab Java wrappers which have been adapted for a more fluid graphic user experience. EpiTools GUI interface is completely portable on all the operative systems where a Java Virtual Machine is deployable, albeit GUI components appearance may differs due to operative system rendering libraries.

EpiTools plugins for Icy

1. Graph creation: spatial and temporal linking

The graph generation algorithm included in CellGraph starts by analyzing the input skeletons to identify the individual polygons representing each cell. We use the java library jts (java topology suite (JTS, 2015) to extract and store the detailed polygonal shape of every cell. Through polygon intersection we discover spatial neighborhood relationships and subsequently store them in a graph structure using the jgraphT library (jGraphT, 2015). To add the temporal linkages we developed a graph-matching algorithm that connects cells from one frame to the next. First we compute a score between cell pairs in different frames according to the cell's polygon overlap and the distance between their centroids. Second, we determine the optimal match between cells with a stable marriage algorithm (Gale and Shapley, 1962). This algorithm determines the best reciprocal match based on the multiple candidates that every cell received from previous frames. Third, we analyze the unmatched cells by heuristics to identify divisions, eliminations or suggest a segmentation mistake. The completed data

structure is stored in memory (i.e. Icy's swimming pool) for subsequent use by one of the other plugins.

2. Exporting the data contained in the spatio-temporal Graph
As stated in the main text, we offer two main export methods. First, from every overlay excel sheets can be generated containing the visualized quantities. To do this it is sufficient that the user clicks on the corresponding overlay in the Icy Layer menu and selects 'Export data to excel' from the options panel below. Second, the use of the CellExport plugin. Here multiple options are available: (1) a larger and more exhaustive excel sheet that contains gathered information from many overlays; (2) an XML based graph format, called GraphML (Brandes et al., 2002), which stores the neighborhood relationships of the cells. GraphML files can be read by many scripting languages such as R or Python. An example analysis file can be downloaded from the project homepage (http://tiny.uzh.ch/dP); (3) a PDF export option is available to generate vector graphics from the overlay using. For this feature we use the gnujpdf library (Beard, 2001); (4) Skeleton export options for faster loading times using the Well-Known-Text format (WKT, https://en.wikipedia.org/wiki/Well-known_text) ; (5) Tracking (if applied) can be exported as csv reducing further loading times.

Supported file formats

1. EpiTools for Matlab – input file formats

We support 8 or 16 bit grey-scale images and use the OME bioformats library for Matlab (Linkert et al., 2010) to import files. While the latter guarantees access to almost all commonly employed microscopy image formats, we currently pose two further requirements: (1) individual files have to contain all information of a time point, i.e. it is not possible to combine multiple files into a single time point. We require this to efficiently concatenate multiple time points; (2) we have limited the possible image file extensions to the following subset to allow fast file inspection: czi, zvi, cxd, ome, ome.tiff, mrc, tif, tiff, lif, lei, ipl, raw, ics, ids, bmp, png, pic, mvd2. It is possible to extend the selection upon request. Preferred image format: Individual TIFF files for every time point. For a simple image format conversion we recommend icy and imageJ, both programs offer very intuitive reformatting options.

2. EpiTools plugins for icy – skeleton file format

Skeleton files are assumed to be 8-bit binary representations of membrane signal where higher values represent membrane signal and lower values background. To ensure 8-connectivity, the imagej function "skeletonize" is applied to every input image. Preferred image format: 8-bit Tiff.

Quick Guides for EpiTools

Disclaimer: This is a static version of the guides present on our website, please consider visiting the website for an up-to-date version which also features images and videos. Link: http://tiny.uzh.ch/dm

Quick-Guide to the EpiTools application for Matlab

EpiTools comes in a single software package for Matlab. We recommend Matlab version (2014a) since we developed and tested EpiTools on this version.

How to install it and receive our latest patches or updates

From the home page of EpiTools you can easily download the latest available version of EpiTools.

1. Extract the package
2. From the directory generated by exploding the package, double click on the launcher file correspondent to your operative system. Done!

In case the launcher does not start (or fails with "EpiTools_mac: Permission denied"), follow these instructions from a new console windows (in OSX, open a new Terminal session) to allow the program launcher to be executed:

1. *cd [download & extraction location]*
2. *chmod +x EpiTools_mac*

How to create a new analysis

Any new analysis generated from EpiTools interface is the result of a guided process where the user fills all the required information via the GUI interface provided.

1. From the main EpiTools interface, click on *File>New Analysis*.
2. Specify the path where the analysis file will be saved.
3. Specify the path where the images have been stored (e.g. use our example files included)
4. Give a name to the analysis you are currently building. (version and department can be useful if you plan to share the analysis)
5. Now turn to the table sub-window, which should list the available images in the selected folder (for the example this will a single file) and
    a. Include the images you would like to analyze by selecting the tick box in the column titled "include?"
    b. To modify more cells at the same time: select the first cell, hold caps, select the last cell, write the new value, press enter
6. Finalize the analysis creation by clicking "Confirm and Proceed"

A side bar should now appear on the left part of the screen. The latter displays all the actions executed so far and is useful for revisiting the analysis (context-menus are available on each node)

Run modules

1. Select a module to run from *Action* menu
2. In case the module requires any additional settings, you will be prompted with a windows where you will be able to specified the required settings.

On analysis module completion, you will be able to visualize the results (if the module generates them) and you will see a new node in the *analysis workflow* tree.

Simple example analysis with included sample (8_bit_sample)

Create a new analysis file for the sample data set. Walk through the following set to produce the skeleton files to be used in EpiTools plugins for ICY. Detailed descriptions of all the modules and parameters are available on our website.

1. Select the *projection module* from the Action menu
    a. This creates a 2D projection from a Z-stack by selectively choosing from which plane to extract each pixel based on a surface estimation.
    b. Run it with default parameters
2. Select the *registration module* from the Action menu
    a. This corrects movement during the acquisition of time series by aligning successive frames to the first.
    b. Choose "StackReg" and Run
3. Select the *Clahe module* from the Action menu
    a. This applies the matlab command adapthisteq® to reduce contrast differences throughout the image
    b. Run it with the default parameters
4. Select the Segmentation module
    a. This detects the individual cells in the image and finds the boundaries with a seed based region growing algorithm.
    b. Run it with the default parameters
5. Select the Skeleton module
    a. This transforms the segmentation output into binary skeletons for every frame. A skeleton depicts the boundaries between cells with a white 1 pixel line (255) over black background (0). File format is PNG.
    b. Run it with default parameters

Done! You can now find the skeleton files in the analysis folder in your chosen "save location"

Additional features

- Correct the segmentation results through our assisted *Tracking Correction module* which allows you modify, add or eliminate seeds from which the Segmentation module discovers cell regions. Re-apply the segmentation on your modifications with the *Re-Segmentation module*
- Test different parameter settings for the same modules by taking advantage of the comparative mode (3rd icon from the right in the menu bar)
- Connect Matlab to icy for best visualization capabilities (2nd icon from the right; this requires the Matlab communicator plugin by Y Montagner to be installed in icy)

Quick-Guide to the EpiTools plugins for ICY

Welcome to the EpiTool plugins for ICY. This collection of plugins for the bioimaging platform icy allows to transform skeleton images into interactive overlays to explore and analyze your data.

Warning: if your icy background appears black instead of grey, the plugins will most likely be affected an unsolved memory leak. Most reported cases appear in co-occurrence with Apple Retina® screens. Temporary Fix: Use an external monitor with the macbook lid closed

Installation: Download & Move the files in the right place

1. Download the package cellGraph_beta.zip from our website (here)
2. Extract the package
3. Place the plugin ***folder davhelle*** into the icy plugin folder (e.g. programs/icy/plugins)
4. Place the workspace file ***EpiTools.xml*** into the icy workspace folder (e.g. programs/icy/workspace)
5. The ***folder test*** does not require any particular location

To update just replace the cellGraph_v#.#.#.jar in the plugin folder davhelle
Release information about latest cellgraph versions: CHANGELOG.TXT
Enable the EpiTools Workspace in Icy

In order to add the **EpiTools toolbar** to your icy installation

1. Enter the preferences (icy logo > preferences)
2. Go to *local workspace* menu
3. Enable the EpiTools workspace by checking the tickbox

Confirm by clicking *Ok*, you will be asked to restart icy to apply the changes.

Install the required plugins for icy

In order to run the EpiTools plugins, only one additional icy plugin is required, EzPlug. This is a plugin which facilitates the building of graphical user interfaces and is used by all EpiTools Plugins. To install it simply write in the icy search field the following plugin name [network connection required]:

- EzPlug SDK (most likely already installed)
- 3D Mesh ROI (only for the CellSurface plugin)

Test if the installation was successful

To test the installation:

1. Run the **TestLoader** Plugin in the EpiTools Bar/or through the search bar
2. You will be asked to locate the **folder test** from the download package
3. Shortly after a new viewer should be visualized (image below)

Generating overlays using CellOverlay

After installing the plugins and loading the test file we are now ready to generate overlays. In fact two overlay are already present if we look at the ***Layer*** menu in the icy menu on the right. By clicking on the eye icon on the side of each overlay you can toggle the presence.

Create a new overlay

Let's add a new overlay to the test viewer (click again on TestLoader if you closed it). Here we will add a graph view overlay which visualizes the neighborhood connectivity of every cell.

1. Open the CellOverlay plugin from the toolbar
2. Select *GRAPH VIEW* from the Overlay list
3. Generate the overlay by clicking *Add Overlay* on the plugin bottom

A new overlay is now present on the image and a new entry in the Layer menu called GraphEdges.

Adjusting a gradient color overlay

When the overlay displays a gradient through a color scheme like in the Area example the user can adjust the scheme through the 4 parameters in the OptionPanel in the Layer menu (see image below). The *Gradient Maximum* and *Gradient Minimum* parameters set the extremes of the gradient (everything above and below has constant color). The *Gradient Scale* is a multiplier between 0 and 1 to scale the color gradient into a particular range of colors (see HSV color scheme for more information about the color space). The *Gradient Shift* is complementary to the scale, shifting the scaled color gradient into the desired region by adding a constant factor between 0 and 1.

As simple exercise let’s add the Area gradient and change colors from [Blue<>Magenta] to [Red<> Blue].

1. Select CELL_AREA from the Overlay list
2. Add the overlay
3. Navigate to the Layer menu and click on the CellArea layer to select it
4. You should now see the Options panel right at the bottom of the layer list
5. Change Scale from 0.5 to 0.7 and Shift from

Export data from an overlay

While we offer a more complete export functionality with the separate CellExport, we can generate an excel sheet (XLS) from every generated overlay. This option can be reached from the layer option menu (see above) where you can also adapt the opacity or delete the overlay. For example to exprot the area of cells from the CELL_AREA overlay do the following:

1. Click on the layer name to make the export option appear
2. Click on the export button and choose where to save the spreadsheet
3. The spreadsheets contains a separate sheet for every frame in the movie

Import your own data with the CellGraph plugin

While the CellOverlay plugin generates the Overlays, it requires a graph structure in the shared plugin memory (also called *swimming pool* in icy). Learn here how to transform your skeleton images into a graph structure using the CellGraph plugin.

Current restrictions:
- Only one graph at the time is allowed in memory.
- Time series require the following pattern: [name]_000.[ext]
- Individual skeleton files, i.e. multidimensional tiffs are not yet supported

1. Choose your Input files

Most importantly CellGraph needs files from which to extract the graph structure:

1. If your files are images/bitmaps the default *File type* 'SKELETON' is correct
   Alternative files are WKT & VKT, both text based
2. Choose your input file

If you want to load a time series select the *First time point*
Please notice the file name convention mentioned in the warning above
Set the number of *Time points to load*, to start it is recommended to try with 1 or 2

2. Review the secondary input options [optional]

In order to correctly analyze the input files CellGraph can be fine tuned for specific file types. By default CellGraph assumes to receive files which were directly exported from the EpiTools App for Matlab.

1. Click the *Packing Analyzer files* if you used the software Packing Analyzer v.2-8 by (Aigouy et al. (2010)) for segmentation
    a. In this scenario use the image as first file. Cell_Graph will use the /handCorrection.png as skeleton
2. Choose *Cut on border line* to exclude the most outer cell layer from the analysis. This is advisable in case of poor performance at the segmentation boundaries
3. Choose *Remove very small cells* to exclude cells below an area threshold automatically, e.g. avoid small segmentation artifacts to be recognized as cells

3. Choose if to track your skeletons

Tracking is only possible if a time series is given as input. The standard tracking *Algorithm* is currently *STABLE_MARRIAGE* (Try the *HUNGARIAN* type for a more robust algorithm to movement but also time consuming!)

Depending on the time series frequency and the segmentation quality it can be helpful to change the default paramenters of the tracking algorithm:

1. Change the *Propagation limit* to amplify(+)/reduce(-) the number of previous frames that participate in the assignment of the track of a cell.
    a. This is useful when the sample is subject to strong motion, a shorter limit will prove more effective
2. Change the *Cut N border lines in 1st frame* to track only a core region of the sample. This is useful if the border region segmentation is poor.

4. Choose the Destination

The extracted graph structures are always used together with a background image. This image can be any image compatible (x, y,t) with the skeleton images, i.e. the original raw image / projected_image / second channel ecc

1. Choose *Image to overay* from you active Sequences
    a. The plugin requires an open image!
2. Deselect *Use ICY-SwimmingPool* only to avoid overriding another structure in memory. This will associate a single tracking view to the selected image.
3. Select *Remove Previous Overlays* if the destination image already overlays which are not needed anymore

6. Run the plugin

To generate the graph click on the triangle button on the lower-left of the plugin.

- During the Execution the progress can be followed both in the status bar as well as in *Output* tab. After execution the first overlay will be displayed on the active sequence.

Generated Result

A. In case of a *Single frame* the found cell polygons with the centroids will be visualized (i.e. the *CELL OUTLINE* overlay)

B. In case of a *Tracked time series* cell the automatically generated overlay will be the *TRACKING* one. Every cell is outlined by an individual color preserved through time and a filled with one of the following colors in case of a tracking event:
   a. [red] cell missing in previous frame
   b. [yellow] cell missing in next frame
   c. [green] cell missing in previous & next
   d. [blue] cell dividing in next frame
   e. [magenta] brother cell missing
   f. [cyan] cell eliminated in next frame
   g. [gray] brother cell was eliminated

Now you can add any overlay by executing the *CellOverlay* plugin, export data with the *CellExport* plugin or Modify the skeletons with *CellEditor*. You can find dedicated tutorials to each of these plugins on our website.

Supplemental References

Beard, E. (2001). gnujpdf, sourceforge.net/projects/gnujpdf/.
Brandes, U., Eiglsperger, M., Herman, I., Himsolt, M., and Marshall, M.S. (2002). GraphML Progress Report Structural Layer Proposal. In Graph Drawing, P. Mutzel, M. Jünger, and S. Leipert, eds. (Springer Berlin Heidelberg), pp. 501-512.
Brodland, G.W., Veldhuis, J.H., Kim, S., Perrone, M., Mashburn, D., and Hutson, M.S. (2014). CellFIT: a cellular force-inference toolkit using curvilinear cell boundaries. In PLoS ONE, pp. e99116.
Cilla, R., Mechery, V., Hernandez De Madrid, B., Del Signore, S., Dotu, I., and Hatini, V. (2015). Segmentation and Tracking of Adherens Junctions in 3D for the Analysis of Epithelial Tissue Morphogenesis. In PLoS Comput Biol, pp. e1004124.
D'Errico, J. (2006). http://ch.mathworks.com/matlabcentral/fileexchange/8998-surface-fitting-using-gridfit.
Dufour, A. http://icy.bioimageanalysis.org/plugin/3D_Mesh_ROI.
Gale, D., and Shapley, L.S. (1962). College Admissions and the Stability of Marriage. The American Mathematical Monthly *69*, 9-15.
Ivan, E.S., Robert, F.S., and Robert, A.S. (1974). A Characterization of Ten Hidden-Surface Algorithms. ACM Comput Surv *6*, 1-55.
jGraphT (2015). a free Java graph library, jgrapht.org.
John, M.S., and Alan, H.B. (1987). Ray tracing complex models containing surface tessellations. In Proceedings of the 14th annual conference on Computer graphics and interactive techniques (ACM).
JTS (2015). JTS Topology Suite sourceforge.net/projects/jts-topo-suite/.
Legoff, L., Rouault, H., and Lecuit, T. (2013). A global pattern of mechanical stress polarizes cell divisions and cell shape in the growing Drosophila wing disc. In Development, pp. 4051-4059.
Linkert, M., Rueden, C.T., Allan, C., Burel, J.-M., Moore, W., Patterson, A., Loranger, B., Moore, J., Neves, C., MacDonald, D., *et al.* (2010). Metadata matters: access to image data in the real world. The Journal of cell biology *189*, 777-782.
Meyer, F. (1994). Topographic distance and watershed lines. Signal Processing *38*, 113-125.
Sage, D. (2012). MIJ: Making Interoperability Between ImageJ and Matlab Possible http://imagejconf.tudor.lu/program/poster/makinginter.
Thevenaz, P., Ruttimann, U.E., and Unser, M. (1998). A pyramid approach to subpixel registration based on intensity. IEEE transactions on image processing : a publication of the IEEE Signal Processing Society *7*, 27-41.
