# Supplementary material for: EpiTools: An Open-Source Image Analysis Toolkit for Quantifying Epithelial Growth Dynamics
Source: Dev Cell. 2016 Jan 11;36(1):103–16. doi: 10.1016/j.devcel.2015.12.012 (PMC4712040; doi:10.1016/j.devcel.2015.12.012)
Supplement: Data S1. Source Code [file mmc2.zip › sourcecode/Quickguide.pdf]

Quick Guides for EpiTools

Disclaimer: This is a static version of the guides present on our website, please consider visiting the website for an up-to-date version which also features images and videos. Link: http://tiny.uzh.ch/dm

Guide written by: EpiTools Team
Email contact: davide.heller@imls.uzh.ch or y.mao@ucl.ac.uk

Table of Contents

Quick Guides for EpiTools 1
Quick-Guide to the EpiTools application for Matlab 1
How to install it and receive our latest patches or updates 1
How to create a new analysis 1
Run modules 2
Simple example analysis with included sample (8_bit_sample) 2
Quick-Guide to the EpiTools plugins for ICY 3
Installation: Download & Move the files in the right place 3
Test if the installation was successful 3
Import your own data with the CellGraph plugin 4
Supported file formats 6
EpiTools for Matlab – input file formats 6
EpiTools plugins for icy – skeleton file format 6

Quick-Guide to the EpiTools application for Matlab

EpiTools comes in a single software package for Matlab. We recommend Matlab version (2014a) since we developed and tested EpiTools on this version.

How to install it and receive our latest patches or updates

From the home page of EpiTools you can easily download the latest available version of EpiTools.

1. Extract the package
2. From the directory generated by exploding the package, double click on the launcher file correspondent to your operative system. Done!

In case the launcher does not start (or fails with "EpiTools_mac: Permission denied"), follow these instructions from a new console windows (in OSX, open a new Terminal session) to allow the program launcher to be executed:

1. *cd [download & extraction location]*
2. *chmod +x EpiTools_mac*

How to create a new analysis

Any new analysis generated from EpiTools interface is the result of a guided process where the user fills all the required information via the GUI interface provided.

1. From the main EpiTools interface, click on *File>New Analysis*.
2. Specify the path where the analysis file will be saved.
3. Specify the path where the images have been stored (e.g. use our example files included)

4. Give a name to the analysis you are currently building. (version and department can be useful if you plan to share the analysis)
5. Now turn to the table sub-window, which should list the available images in the selected folder (for the example this will a single file) and
    a. Include the images you would like to analyze by selecting the tick box in the column titled “include?”
    b. To modify more cells at the same time: select the first cell, hold caps, select the last cell, write the new value, press enter
6. Finalize the analysis creation by clicking “Confirm and Proceed”

A side bar should now appear on the left part of the screen. The latter displays all the actions executed so far and is useful for revisiting the analysis (context-menus are available on each node)

Run modules

1. Select a module to run from *Action* menu
2. In case the module requires any additional settings, you will be prompted with a windows where you will be able to specified the required settings.

On analysis module completion, you will be able to visualize the results (if the module generates them) and you will see a new node in the *analysis workflow* tree.

Simple example analysis with included sample (8_bit_sample)

Create a new analysis file for the sample data set. Walk through the following set to produce the skeleton files to be used in EpiTools plugins for ICY. Detailed descriptions of all the modules and parameters are available on our website.

1. Select the *projection module* from the Action menu
    a. This creates a 2D projection from a Z-stack by selectively choosing from which plane to extract each pixel based on a surface estimation.
    b. Run it with default parameters
2. Select the *registration module* from the Action menu
    a. This corrects movement during the acquisition of time series by aligning successive frames to the first.
    b. Choose “StackReg” and Run
3. Select the *Clahe module* from the Action menu
    a. This applies the matlab command adapthisteq® to reduce contrast differences throughout the image
    b. Run it with the default parameters
4. Select the Segmentation module
    a. This detects the individual cells in the image and finds the boundaries with a seed based region growing algorithm.
    b. Run it with the default parameters
5. Select the Skeleton module
    a. This transforms the segmentation output into binary skeletons for every frame. A skeleton depicts the boundaries between cells with a white 1 pixel line (255) over black background (0). File format is PNG.
    b. Run it with default parameters

Done! You can now find the skeleton files in the analysis folder in your chosen “save location”

Additional features

- Correct the segmentation results through our assisted *Tracking Correction module* which allows you modify, add or eliminate seeds from which the Segmentation module discovers

cell regions. Re-apply the segmentation on your modifications with the *Re-Segmentation module*

- Test different parameter settings for the same modules by taking advantage of the comparative mode (3rd icon from the right in the menu bar)
- Connect Matlab to icy for best visualization capabilities (2nd icon from the right; this requires the Matlab communicator plugin by Y Montagner to be installed in icy)

Quick-Guide to the EpiTools plugins for ICY

Welcome to the EpiTool plugins for ICY. This collection of plugins for the bioimaging platform icy allows to transform skeleton images into interactive overlays to explore and analyze your data.

Warning: if your icy background appears black instead of grey, the plugins will most likely be affected an unsolved memory leak. Most reported cases appear in co-occurrence with Apple Retina® screens. Temporary Fix: Use an external monitor with the macbook lid closed

Installation: Download & Move the files in the right place

1. Download the package cellGraph_beta.zip from our website (here)
2. Extract the package
3. Place the plugin ***folder davhelle*** into the icy plugin folder (e.g. programs/icy/plugins)
4. Place the workspace file ***EpiTools.xml*** into the icy workspace folder (e.g. programs/icy/workspace)
5. The ***folder test*** does not require any particular location

To update just replace the cellGraph_v#.#.#.jar in the plugin folder davhelle
Release information about latest cellgraph versions: CHANGELOG.TXT
Enable the EpiTools Workspace in Icy

In order to add the **EpiTools toolbar** to your icy installation

1. Enter the preferences (icy logo > preferences)
2. Go to *local workspace* menu
3. Enable the EpiTools workspace by checking the tickbox

Confirm by clicking *Ok*, you will be asked to restart icy to apply the changes.

Install the required plugins for icy

In order to run the EpiTools plugins, only one additional icy plugin is required, EzPlug. This is a plugin which facilitates the building of graphical user interfaces and is used by all EpiTools Plugins. To install it simply write in the icy search field the following plugin name [network connection required]:

- EzPlug SDK (most likely already installed)
- 3D Mesh ROI (only for the CellSurface plugin)

Test if the installation was successful

To test the installation:

1. Run the **TestLoader** Plugin in the EpiTools Bar/or through the search bar
2. You will be asked to locate the **folder test** from the download package
3. Shortly after a new viewer should be visualized (image below)

Generating overlays using CellOverlay

After installing the plugins and loading the test file we are now ready to generate overlays. In fact two overlay are already present if we look at the ***Layer*** menu in the icy menu on the right. By clicking on the eye icon on the side of each overlay you can toggle the presence.

Create a new overlay

Let's add a new overlay to the test viewer (click again on TestLoader if you closed it). Here we will add a graph view overlay which visualizes the neighborhood connectivity of every cell.

1. Open the CellOverlay plugin from the toolbar
2. Select *GRAPH VIEW* from the Overlay list
3. Generate the overlay by clicking *Add Overlay* on the plugin bottom

A new overlay is now present on the image and a new entry in the Layer menu called GraphEdges.

Adjusting a gradient color overlay

When the overlay displays a gradient through a color scheme like in the Area example the user can adjust the scheme through the 4 parameters in the OptionPanel in the Layer menu (see image below). The *Gradient Maximum* and *Gradient Minimum* parameters set the extremes of the gradient (everything above and below has constant color). The *Gradient Scale* is a multiplier between 0 and 1 to scale the color gradient into a particular range of colors (see HSV color scheme for more information about the color space). The *Gradient Shift* is complementary to the scale, shifting the scaled color gradient into the desired region by adding a constant factor between 0 and 1.

As simple exercise let’s add the Area gradient and change colors from [Blue<>Magenta] to [Red<> Blue].

1. Select CELL_AREA from the Overlay list
2. Add the overlay
3. Navigate to the Layer menu and click on the CellArea layer to select it
4. You should now see the Options panel right at the bottom of the layer list
5. Change Scale from 0.5 to 0.7 and Shift from

Export data from an overlay

While we offer a more complete export functionality with the separate CellExport, we can generate an excel sheet (XLS) from every generated overlay. This option can be reached from the layer option menu (see above) where you can also adapt the opacity or delete the overlay. For example to exprot the area of cells from the CELL_AREA overlay do the following:

1. Click on the layer name to make the export option appear
2. Click on the export button and choose where to save the spreadsheet
3. The spreadsheets contains a separate sheet for every frame in the movie

Import your own data with the CellGraph plugin

While the CellOverlay plugin generates the Overlays, it requires a graph structure in the shared plugin memory (also called *swimming pool* in icy). Learn here how to transform your skeleton images into a graph structure using the CellGraph plugin.

Current restrictions:
- Only one graph at the time is allowed in memory.
- Time series require the following pattern: [name]_000.[ext]
- Individual skeleton files, i.e. multidimensional tiffs are not yet supported

1. Choose your Input files

Most importantly CellGraph needs files from which to extract the graph structure:

1. If your files are images/bitmaps the default *File type* 'SKELETON' is correct
   Alternative files are WKT & VKT, both text based
2. Choose your input file
   If you want to load a time series select the *First time point*
   Please notice the file name convention mentioned in the warning above

Set the number of *Time points to load*, to start it is recommended to try with 1 or 2

2. Review the secondary input options [optional]

In order to correctly analyze the input files CellGraph can be fine tuned for specific file types. By default CellGraph assumes to receive files which were directly exported from the EpiTools App for Matlab.

1. Click the *Packing Analyzer files* if you used the software Packing Analyzer v.2-8 by (Aigouy et al. (2010)) for segmentation
    a. In this scenario use the image as first file. Cell_Graph will use the /handCorrection.png as skeleton
2. Choose *Cut on border line* to exclude the most outer cell layer from the analysis. This is advisable in case of poor performance at the segmentation boundaries
3. Choose *Remove very small cells* to exclude cells below an area threshold automatically, e.g. avoid small segmentation artifacts to be recognized as cells

3. Choose if to track your skeletons

Tracking is only possible if a time series is given as input. The standard tracking *Algorithm* is currently *STABLE_MARRIAGE* (Try the *HUNGARIAN* type for a more robust algorithm to movement but also time consuming!)

Depending on the time series frequency and the segmentation quality it can be helpful to change the default paramenters of the tracking algorithm:

1. Change the *Propagation limit* to amplify(+)/reduce(-) the number of previous frames that participate in the assignment of the track of a cell.
    a. This is useful when the sample is subject to strong motion, a shorter limit will prove more effective
2. Change the *Cut N border lines in 1st frame* to track only a core region of the sample. This is useful if the border region segmentation is poor.

4. Choose the Destination

The extracted graph structures are always used together with a background image. This image can be any image compatible (x, y,t) with the skeleton images, i.e. the original raw image / projected_image / second channel ecc

1. Choose *Image to overay* from you active Sequences
    a. The plugin requires an open image!
2. Deselect *Use ICY-SwimmingPool* only to avoid overriding another structure in memory. This will associate a single tracking view to the selected image.
3. Select *Remove Previous Overlays* if the destination image already overlays which are not needed anymore

6. Run the plugin

To generate the graph click on the triangle button on the lower-left of the plugin.

- During the Execution the progress can be followed both in the status bar as well as in *Output* tab. After execution the first overlay will be displayed on the active sequence.

Generated Result

A. In case of a *Single frame* the found cell polygons with the centroids will be visualized (i.e. the *CELL OUTLINE* overlay)

B. In case of a *Tracked time series* cell the automatically generated overlay will be the *TRACKING* one. Every cell is outlined by an individual color preserved through time and a filled with one of the following colors in case of a tracking event:
    a. [red] cell missing in previous frame
    b. [yellow] cell missing in next frame

c. [green] cell missing in previous & next
d. [blue] cell dividing in next frame
e. [magenta] brother cell missing
f. [cyan] cell eliminated in next frame
g. [gray] brother cell was eliminated

Now you can add any overlay by executing the *CellOverlay* plugin, export data with the *CellExport* plugin or Modify the skeletons with *CellEditor*. You can find dedicated tutorials to each of these plugins on our website.

Supported file formats

EpiTools for Matlab – input file formats

We support 8 or 16 bit grey-scale images and use the OME bioformats library for Matlab (Linkert et al., 2010) to import files. While the latter guarantees access to almost all commonly employed microscopy image formats, we currently pose two further requirements: (1) individual files have to contain all information of a time point, i.e. it is not possible to combine multiple files into a single time point. We require this to efficiently concatenate multiple time points; (2) we have limited the possible image file extensions to the following subset to allow fast file inspection: czi, zvi, cxd, ome, ome.tiff, mrc, tif, tiff, lif, lei, ipl, raw, ics, ids, bmp, png, pic, mvd2. It is possible to extend the selection upon request. Preferred image format: Individual TIFF files for every time point. For a simple image format conversion we recommend icy and imageJ, both programs offer very intuitive reformatting options.

EpiTools plugins for icy – skeleton file format

Skeleton files are assumed to be 8-bit binary representations of membrane signal where higher values represent membrane signal and lower values background. To ensure 8-connectivity, the imagej function "skeletonize" is applied to every input image. Preferred image format: 8-bit Tiff.
